# Safety and feasibility of ultrasound-triggered targeted drug delivery of doxorubicin from thermosensitive liposomes in liver tumours (TARDOX): a single-centre, open-label, phase 1 trial

**DOI:** 10.1016/S1470-2045(18)30332-2

**Published:** 2018-08

**Authors:** Paul C Lyon, Michael D Gray, Christophoros Mannaris, Lisa K Folkes, Michael Stratford, Leticia Campo, Daniel Y F Chung, Shaun Scott, Mark Anderson, Robert Goldin, Robert Carlisle, Feng Wu, Mark R Middleton, Fergus V Gleeson, Constantin C Coussios

**Affiliations:** a Nuffield Department of Surgical Sciences, John Radcliffe Hospital, Oxford University Hospitals NHS Foundation Trust, Oxford, UK; b Nuffield Department of Anaesthetics, John Radcliffe Hospital, Oxford University Hospitals NHS Foundation Trust, Oxford, UK; c Department of Radiology, Churchill Hospital, Oxford University Hospitals NHS Foundation Trust, Oxford, UK; d Department of Oncology, Churchill Hospital, Oxford University Hospitals NHS Foundation Trust, Oxford, UK; e Institute of Biomedical Engineering, University of Oxford, Oxford, UK; f CRUK/MRC Oxford Institute for Radiation Oncology, Department of Oncology, University of Oxford, Oxford, UK; g Centre for Pathology, Faculty of Medicine, Imperial College London, London, UK

## Abstract

**Background:**

Previous preclinical research has shown that extracorporeal devices can be used to enhance the delivery and distribution of systemically administered anticancer drugs, resulting in increased intratumoural concentrations. We aimed to assess the safety and feasibility of targeted release and enhanced delivery of doxorubicin to solid tumours from thermosensitive liposomes triggered by mild hyperthermia, induced non-invasively by focused ultrasound.

**Methods:**

We did an open-label, single-centre, phase 1 trial in a single UK hospital. Adult patients (aged ≥18 years) with unresectable and non-ablatable primary or secondary liver tumours of any histological subtype were considered for the study. Patients received a single intravenous infusion (50 mg/m^2^) of lyso-thermosensitive liposomal doxorubicin (LTLD), followed by extracorporeal focused ultrasound exposure of a single target liver tumour. The trial had two parts: in part I, patients had a real-time thermometry device implanted intratumourally, whereas patients in part II proceeded without thermometry and we used a patient-specific model to predict optimal exposure parameters. We assessed tumour biopsies obtained before and after focused ultrasound exposure for doxorubicin concentration and distribution. The primary endpoint was at least a doubling of total intratumoural doxorubicin concentration in at least half of the patients treated, on an intention-to-treat basis. This study is registered with ClinicalTrials.gov, number NCT02181075, and is now closed to recruitment.

**Findings:**

Between March 13, 2015, and March 27, 2017, ten patients were enrolled in the study (six patients in part I and four in part II), and received a dose of LTLD followed by focused ultrasound exposure. The treatment resulted in an average increase of 3·7 times in intratumoural biopsy doxorubicin concentrations, from an estimate of 2·34 μg/g (SD 0·93) immediately after drug infusion to 8·56 μg/g (5·69) after focused ultrasound. Increases of two to ten times were observed in seven (70%) of ten patients, satisfying the primary endpoint. Serious adverse events registered were expected grade 4 transient neutropenia in five patients and prolonged hospital stay due to unexpected grade 1 confusion in one patient. Grade 3–4 adverse events recorded were neutropenia (grade 3 in one patient and grade 4 in five patients), and grade 3 anaemia in one patient. No treatment-related deaths occurred.

**Interpretation:**

The combined treatment of LTLD and non-invasive focused ultrasound hyperthermia in this study seemed to be clinically feasible, safe, and able to enhance intratumoural drug delivery, providing targeted chemo-ablative response in human liver tumours that were refractory to standard chemotherapy.

**Funding:**

Oxford Biomedical Research Centre, National Institute for Health Research.

## Introduction

A major challenge of systemic anticancer therapy is achieving the delivery of a therapeutic dose to the tumour without exceeding the maximum tolerated dose and causing toxicity in healthy tissues.^1^ Several device-based approaches are under investigation to address this challenge, including the use of focused ultrasound,^2^ magnetic field, laser, radio-frequency, and microwave techniques for activation of stimuli-responsive drug delivery systems.^3^

Liposomes are biocompatible phospholipid nano-particles that can enhance the pharmacokinetics of chemotherapeutic drugs and therefore are attractive drug delivery systems.^4^ A considerable body of preclinical research has shown that hyperthermia-triggered release of a therapeutic drug encapsulated in thermosensitive liposomal carriers can greatly enhance the intratumoural concentration, distribution, and ultimate therapeutic efficacy of a given systemic dose.^5^, ^6^, ^7^, ^8^ However, these effects have not yet been proven clinically. Focused ultrasound is the only non-invasive approach capable of generating highly targeted mild hyperthermia at depth within the body and is thus an attractive method for triggered drug release.

**Research in context**

**Evidence before this study**

Achievement of targeted and efficacious drug delivery while avoiding off-target toxicity represents a universal challenge in oncology, especially for the treatment of metastatic cancer. Nanotechnology vehicles that passively accumulate or selectively release anticancer agents in solid tumours have shown substantial promise in preclinical studies but have not yet gained widespread adoption because of the absence of demonstrable clinical benefit. Lyso-thermosensitive liposomal doxorubicin (LTLD) is a thermally active liposomal drug delivery system capable of selectively releasing its cargo (doxorubicin) under conditions of mild hyperthermia. Previous clinical studies with LTLD have used it solely in combination with minimally invasive radiofrequency ablation to improve treatment of tumour margins. However, preclinical studies showed that, by using focused ultrasound to induce mild hyperthermia non-invasively after systemic administration of LTLD, the intratumoural delivery and distribution for a given systemic dose of doxorubicin can be greatly enhanced, potentially increasing therapeutic efficacy. The feasibility and applicability of these effects have not yet been demonstrated clinically. Because of the small number and heterogeneous nature of preclinical studies and the absence of clinical studies, we did not do a formal meta-analysis of existing literature in ultrasound-mediated targeted drug delivery.

**Added value of this study**

To our knowledge, this clinical study is the first to investigate the safety and feasibility of extracorporeally triggered drug release in oncology and to quantify the potential benefits of this approach in terms of the drug dose, distribution, and cellular delivery, in addition to radiologically assessing the observed therapeutic response induced by a single systemically administered course of chemotherapy. Our study showed the safety and feasibility of selectively maintaining non-ablative hyperthermia in tumours with volume up to 100 cm^3^ in the liver, by use of a clinically approved extracorporeal focused ultrasound device. Additionally, our study showed quantitative measures of the pharmacokinetics and intratumoural drug accumulation derived from the use of liposomal carriers in several tumour subtypes, both before and after ultrasound exposure, providing much needed clinical data about the value of passive versus active methods of accumulating therapeutic agents in tumours.

**Implications of all the available evidence**

Our study showed the potential to attain a chemo-ablative response in otherwise refractory tumours when using a thermosensitive liposomal drug carrier in combination with non-invasive focused ultrasound. Building on decades of promising preclinical research, in both therapeutic ultrasound and drug delivery systems, our study highlights the clinical potential of device-based drug delivery approaches in general, and ultrasound in particular, to achieve several-fold enhancements in the delivery and distribution of existing and future therapeutic agents to solid tumours, with potentially transformative implications for their therapeutic effectiveness at a given systemic dose.

Unlike non-thermosensitive liposomal doxorubicin (NTLD), lyso-thermosensitive liposomal doxorubicin (LTLD; Celsion Corporation, Lawrenceville, NJ, USA) releases its drug payload at temperatures above 39·5°C.^6^ Liposomal formulations enhance plasma pharmacokinetics of doxorubicin, resulting in a plasma half-life in humans of about 10 h for NTLD^9^ and 1 h for LTLD,^10^—much longer than that of free doxorubicin, which has a half-life of only minutes.^10^

The thermosensitive property of LTLD has been used clinically, mainly in conjunction with minimally invasive radiofrequency ablation, which is used to thermally ablate the core of the tumour while LTLD is circulating systemically, with the intention of improving therapy at the tumour margins.^11^ LTLD was evaluated as part of the HEAT trial,^12^ a pivotal phase 3 study of the use of radiofrequency ablation for the treatment of hepatocellular carcinoma. The primary endpoint of the HEAT trial was not met; in the intention-to-treat analysis, the progression-free survival hazard ratio (HR) of radiofrequency ablation plus LTLD versus radiofrequency ablation alone was 0·96 (95% CI 0·79–1·18; p=0·71) and the overall survival HR was 0·95 (0·76–1·20; p=0·67). However, in a subgroup of 285 patients with a solitary hepatocellular carcinoma lesion who received a radiofrequency ablation dwell time of at least 45 min, the overall survival HR was 0·63 (0·41–0·96; p<0·05) in favour of combination therapy. The positive findings in this subgroup of the HEAT study led to the ongoing OPTIMA study (NCT02112656), which is a randomised, double-blind, dummy-controlled, phase 3 clinical study of LTLD used in combination with standardised radiofrequency ablation for 45 min or longer for solitary hepatocellular carcinoma. These radiofrequency ablation studies have shown an acceptable safety profile, but targeted LTLD release using extracorporeal focused ultrasound, to our knowledge, has never been assessed in humans. Therefore, we did a phase 1 trial to assess the safety and feasibility of targeted release and enhanced delivery of doxorubicin from thermosensitive liposomes triggered by mild hyperthermia, induced non-invasively by focused ultrasound.

## Methods

### Study design and participants

The TARDOX study was a phase 1, single-centre, open-label study, done at Churchill Hospital (Oxford, UK). The Health Research Authority National Research Ethics Service, the Oxford University Hospitals Research and Development Department, and the UK's Medicines and Healthcare products Regulatory Agency granted ethics and regulatory approvals. The full study protocol is available in the appendix and a detailed account of the protocol design is available elsewhere.^13^

Patients who were considered for this study were aged 18 years or older and had pathologically confirmed incurable solid primary or secondary (metastatic) liver tumours, of any histological subtype, who had progressed or were stable on conventional chemotherapy. For inclusion, patients were required to have at least one liver tumour (≥1 cm in size) amenable to ultrasound-guided intervention, a life expectancy of 3 months or longer, a left ventricular ejection fraction of 50% or greater, a WHO performance status of 1 or lower, and adequate haematological and biochemical indices. Patients who had received radiotherapy to the target region in the preceding 12 months or a lifetime dose of doxorubicin greater than 450 mg/m^2^ were excluded, as were patients who were pregnant or had HIV-positive status, haemochromatosis, uncontrolled diabetes, ongoing infection, advanced liver disease, or serious illness—including congestive heart failure, myocardial infarction, or stroke—within the previous 6 months. Detailed inclusion and exclusion criteria are provided in the full study protocol (appendix p 28) and the protocol design.^13^ Patients were recruited from the early phase trials clinic at the Churchill Hospital. All patients gave written consent for study participation.

### Procedures

Participants were assigned to one of two parts of the study, delineated by the presence (part I) or absence (part II) of a clinically approved thermometry device temporarily implanted percutaneously in the target tumour during the intervention (appendix p 4). In part I, real-time thermometry data were acquired during focused ultrasound exposure by the implanted thermometry device (thermistor or thermocouple). Intratumoural drug concentration was estimated by use of tumour biopsies taken before drug infusion (pre-LTLD), after infusion (post-LTLD), and after ultrasound exposure (post-LTLD plus focused ultrasound), through the same co-axial needle used to implant the thermometry device. In addition to assessing safety, feasibility, and efficacy of ultrasound-triggered targeted drug delivery, part I was also designed to capture thermometry data to help predict ultrasound parameters for part II of the study.

Part II of the study was opened subsequently and was designed to assess the feasibility and efficacy of drug delivery without invasive thermometry, to better reflect how this treatment might be ultimately implemented non-invasively in routine clinical practice. Focused ultrasound treatment parameters were defined by a predictive model to scale the ultrasound power and duty cycle as a function of the treatment depth, to yield a temperature in the range of 39·5–43°C on the intended treatment volume. In part II of the study, after drug delivery, only the post-LTLD plus focused ultrasound biopsy was taken. Clinical data from both parts of the study were used in endpoint analysis, which included quantification of the delivered intratumoural dose of doxorubicin from tumour biopsies.

For all study participants, before full study consent, ultrasound planning sessions were done to assess the feasibility of targeting liver lesions with ultrasound, resulting in the selection of a single target tumour for each patient for intervention. On the day before treatment, baseline scans (perfusion CT and dynamic contrast-enhanced MRI of the liver, and ^18^F-fluorodeoxyglucose PET-CT scans were done and the required pre-medications (steroids) were given. Treatment consisted of one cycle of LTLD administered during a single interventional procedure. The treatment was received under a general anaesthetic (with the use of high-frequency jet ventilation to reduce respiratory motion of the liver) and involved a single 30-min intravenous infusion of LTLD (50 mg/m^2^) followed by targeted hyperthermia of the selected liver tumour by focused ultrasound. In the focused ultrasound treatment, a CE-marked extracorporeal high-intensity focused ultrasound device, certified for oncological treatment (model JC200 Focused Ultrasound Tumor Therapeutic System, Chongqing Haifu, Chongqing, China), operating at a frequency of 0·96 MHz, was used to induce highly targeted mild hyperthermia (≥39·5°C) within the selected liver tumours. For all part I and part II interventions, the integrated diagnostic B-mode ultrasound probe of the device was used for image guidance through intercostal or subcostal windows. Extensive preclinical validation of the clinical device for its application in volumetric hyperthermia, using the same thermometry devices that were used in this study, was done before patient intervention (Lyon PC, unpublished). Patients were discharged from hospital the following morning, after a clinical review and MRI scan. The intervention is further detailed in the appendix and protocol design.^13^

Clinical reviews and routine blood tests were done at 2 weeks and 4 weeks post-intervention, coinciding with repeat radiological imaging (CT and MRI scans of the liver and ^18^F-fluorodeoxyglucose PET-CT scan). During the 30-day follow-up period, adverse events were recorded using Common Terminology Criteria for Adverse Events, version 4.0 (CTCAE v4.0). After the trial period, patients either resumed standard of care (typically supportive treatments without further systemic chemotherapy) or were recruited for further early-phase trials. Patients could be removed from the study according to their wishes or clinician decision, if toxic effects or adverse events were deemed unacceptable, if there were substantial protocol deviations or non-compliance, or if there were new exclusion criteria, such as pregnancy.

Detailed sample analysis methods are outlined in the appendix (pp 5, 6). In both parts of the study, peripheral blood samples were obtained pre-LTLD, post-LTLD, and post-LTLD plus focused ultrasound for pharmacokinetic analysis, corresponding with part I biopsy timepoints. Plasma aliquots and weighed biopsy samples were stored at −80°C until subsequent analysis by high-performance liquid chromatography (HPLC), with fluorescence detection at 480 nm excitation and 560 nm emission, to assess the total doxorubicin concentration within each biopsy and plasma sample, whether liposomal or free. We used daunorubicin as an internal standard to improve accuracy. Because of the solvent drug-extraction technique used, it was not possible to independently measure liposomal and free (bioavailable) concentrations of doxorubicin in tissue. Therefore, only total tissue concentrations are reported. Plasma aliquots obtained at the same timepoints were also analysed on the same day by a dequenching assay, in an attempt to estimate the degree of encapsulation of doxorubicin in plasma using direct fluorometry (appendix p 12). In part II of the study, biopsies were only done after LTLD plus focused ultrasound treatment. Therefore, for part II of the study, the mean drug concentration of the part I post-LTLD treatment biopsies was used as a comparator to evaluate the number of patients with at least a two-times increase in intratumoural biopsy drug concentration.

The focused ultrasound-targeted tumours were analysed for response by PCL or other members of the radiology team with Choi criteria, Response Evaluation Criteria in Solid Tumors (RECIST), and PET Response Criteria in Solid Tumors (PERCIST), each modified for a single target, by CT, MRI, and PET-CT imaging.^14^, ^15^, ^16^ Additionally, target tumours were also analysed for total lesion glycolysis by PET-CT scan^17^ (appendix p 6). Non-target liver tumours that had received a drug dose but no focused ultrasound exposure were also assessed in isolation in the same manner, with the same scan sequences, to provide time-matched radiological controls.

### Outcomes

The primary objective of this study was to assess the feasibility of targeted release of doxorubicin from LTLD by use of mild hyperthermia generated non-invasively by focused ultrasound. The primary endpoint was defined as at least a doubling of the intratumoural biopsy doxorubicin concentrations, or final concentrations greater than 10 μg/g, after focused ultrasound-mediated hyperthermia, in at least half of patients treated.

Secondary objectives were related to assessment of the safety and optimisation of the ultrasound exposure parameters for targeted liver hyperthermia and drug release. Therefore, secondary endpoints were defined as the achievement of mild hyperthermia as monitored by the implanted thermometry device for patients in part I; persistence of cell viability staining after focused ultrasound exposure, to indicate absence of instantaneous thermal ablation; and adverse events occurring in the 30 days after the intervention relating to either LTLD or focused ultrasound procedure, including clinically significant bone marrow suppression and liver toxicity.

Prespecified exploratory (tertiary) objectives investigated alternative methods of quantifying doxorubicin release (as opposed to estimating intratumoural concentrations) and the therapeutic effect of the intervention on the target tumours. Therefore, tertiary endpoints included positive fluorescence in tumour biopsies (which would be indicative of bioavailable doxorubicin), and radiological evidence of response in target tumour volumes up to 30 days after the intervention, according to RECIST and Choi response evaluation criteria based on MRI and CT scans, and SUV_max_ using PET-CT, with each criteria modified for a single target.

### Statistical analysis

This study tested the hypothesis that higher intratumoural drug concentrations could be achieved when combining LTLD with focused ultrasound-induced hyperthermia for targeted tumour delivery, compared with passive accumulation alone. We chose the sample size of up to 28 patients on the basis of ethical considerations and available funding. We did not do a formal sample size power calculation. All outcome measures were assessed on an intention-to-treat basis, regardless of whether or not satisfactory hyperthermia was achieved. All evaluable patients from parts I and II of the trial were included in the endpoint analyses. Patients were excluded from the primary outcome measure if validated HPLC analysis of tumour tissue was not available. This study did not require statistical analysis; a doubling of total intratumoural doxorubicin concentrations (post-LTLD *vs* post-LTLD plus focused ultrasound) in at least half of the evaluable participants was required to meet the primary endpoint. After treatment of the first four patients in part I of the study, the trial management group did an interim analysis of the secondary endpoint relating to optimal focused ultrasound exposure parameters, in addition to the remaining secondary endpoints pertaining to safety. Because hyperthermia higher than the drug release threshold had been reliably attained in each target liver tumour, and no safety concerns were raised, part II of the trial was opened in parallel to part I. From this point on, allocation to either part was determined on the basis of feasibility (anatomical location of the potential target tumours) and study team and patient preference, as detailed in the full study protocol. All other analyses were done at the end of the study.

This study is registered with ClinicalTrials.gov, number NCT02181075, and Eudra-CT, number 2014–000514–61.

### Role of the funding source

The funders and sponsor of the study had no role in study design, data collection, data analysis, data interpretation, or writing of the report. PCL, RC, MRM, FVG, and CCC had access to all the raw data. LKF and MS had access to HPLC data. MDG, CM, DYFC, SS, MA, and FW had access to thermometry and radiology data. LC and RG had access to microscopy data. The corresponding author had full access to all the data in the study and had final responsibility for the decision to submit for publication.

## Results

**Figure 1:**
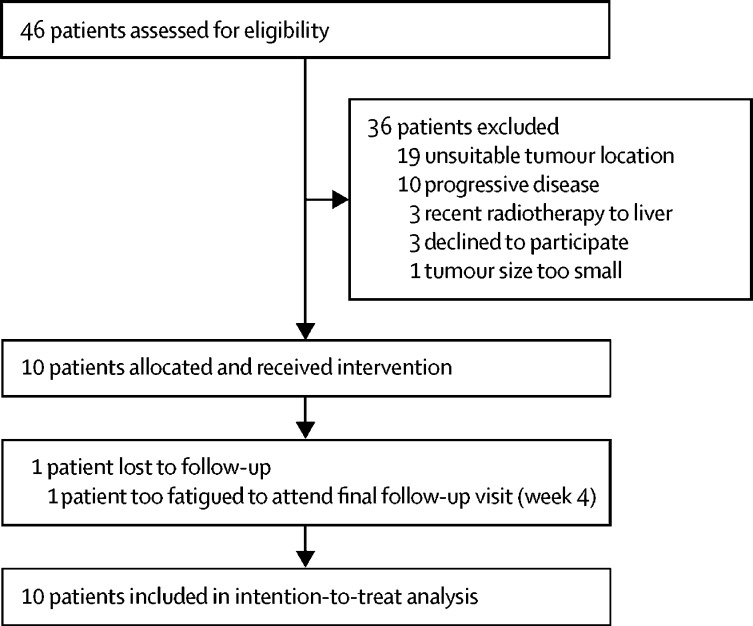
Trial profile

Of 46 patients who were screened and assessed for eligibility, ten patients satisfied the inclusion criteria and were enrolled between March 13, 2015, and March 27, 2017. The remaining 36 patients did not proceed with the study: 33 were ultimately ineligible and the remaining three patients declined to participate (figure 1). Of the excluded patients, the majority (19 [53%] of 36) were excluded on the basis of anatomical location of the tumour, before part II was open to recruitment, which offered much more flexibility in tumour location. All ten enrolled patients received the intervention, with a median follow-up duration of 29·5 days (IQR 29–30 days, table 1). Various liver malignancies were targeted for therapy, including colorectal, breast, and lung metastases and primary hepatocellular carcinoma (table 1). Six patients were recruited to part I and four patients to part II of the study. Patients are identified in this report by study part (I or II) and index—for example, II.03 indicates the third patient treated with the part II protocol. There were no treatment-related deaths during the study.

**Table 1:** Patient characteristics

	**Patient I.01**	**Patient I.02**	**Patient I.03**	**Patient I.04**	**Patient I.05**	**Patient I.06**	**Patient II.01**	**Patient II.02**	**Patient II.03**	**Patient II.04**
Age	50	68	66	64	49	65	53	63	53	65
Sex	Male	Female	Male	Female	Male	Male	Male	Female	Female	Male
BMI, kg/m^2^	22·6	22·9	25·0	25·7	26·3	25·9	37·3	25·2	27·0	27·0
WHO performance status	0	0	0	0	1	0	1	0	1	0
ASA grade	2	2	2	3	2	2	3	3	3	3
Enrolment date	March 13, 2015	May 5, 2015	Sept 18, 2015	Oct 28, 2015	March 4, 2016	March 27, 2017	June 15, 2016	July 18, 2016	Nov 7, 2016	Jan 9, 2017
Treatment date	March 24, 2015	May 6, 2015	Sept 23, 2015	Nov 4, 2015	March 9, 2016	March 29, 2017	June 22, 2016	July 20, 2016	Nov 9, 2016	Jan 11, 2017
Last follow-up date	April 22, 2015	June 8, 2015	Oct 22, 2015	Dec 4, 2015	April 8, 2016	April 27, 2017	July 25, 2016	Aug 19, 2016	Nov 30, 2016	Feb 9, 2017
Diagnosis	Diffuse multi-lobular hepatocellular carcinoma	Metastatic ductal breast carcinoma (ER+, PR+)	Metastatic colorectal adenocarcinoma (*KRAS*+)	Metastatic colorectal adenocarcinoma (*RAS* mutant)	Metastatic colorectal adenocarcinoma (*KRAS*+)	Metastatic colorectal adenocarcinoma (*KRAS*+)	Metastatic squamous cell lung cancer	Metastatic colorectal carcinoma (Dukes C1, G2 T4 N2 Mx) (*KRAS*+, *NRAS*+, *BRAF* wildtype)	Metastatic colorectal adenocarcinoma (*KRAS*+)	Metastatic colorectal adenocarcinoma (*KRAS*+)
Previous treatments (chronological order)	Nemorubicin + cisplatin, pemetrexed + cisplatin + PARP inhibitor (A4991014 early-phase study)	Breast resection, radiotherapy, arimidex, exemestane, tamoxifen, faslodex, capecitabine, SIRT, denosumab, megace, vinorelbine	Bowel resection, irinotecan, modified de Gramont chemotherapy and bevacizumab, liver resection and RFA, CAPOX, AZD0424 (early-phase study), BAY17437 (early-phase study)	Bevacizumab + 5-FU, irinotecan + 5-FU, BAY1238097 – BETI-CID-7 (early-phase study), surgery for bowel obstruction	FOLFOX and FOLFIRI chemotherapy	FOLFOX chemotherapy + cetuximab, bowel resection, FOLFIRI chemotherapy, trifluridine-tipiracil	Radiotherapy + cisplatin + etoposide, paclitaxel + AZD2014 (TAX-TORC early-phase study)	Bowel resection, oxaliplatin + capecitabine, FOLFIRI chemotherapy + cetuximab, mytomycin C + capecitabine, further palliative laparotomy for Krukenburg tumour	Oxaliplatin + 5-FU, liver resection, irinotecan + capectiabine, oxaliplatin	Bowel resection, oxaliplatin + capecitabine (SCOT trial), irinotecan + 5-FU, oxaliplatin + 5-FU
Comorbidities	Tetralogy of Fallot surgery as child, well controlled asthma	..	Hypertension, pulmonary embolism, atrial fibrillation, GORD, hypercholesterolaemia	Oophrectomy (cyst), osteoporosis, cataract, GORD, abdominal wall hernia	Hypertension	Coeliac disease, deep vein thrombosis	NIDD, gout, hypertension, hypercholesterolaemia	Ablated Wolff-Parkinson-White syndrome, hypertension, NIDD, endometrial polyps, deep vein thrombosis	Pulmonary embolus, deep vein thrombosis, superior vena cava filter	Depression, hypertension, transient ischaemic attack

Both partial and complete previous chemotherapy treatment programmes are included. BMI=body-mass index. ASA=American Society of Anesthesiologists. ER+=oestrogen receptor positive. PR+=progesterone receptor positive. *KRAS*+=*KRAS* mutation positive. *NRAS+*=*NRAS* mutation positive. PARP=poly (ADP-ribose) polymerase. RFA=radiofrequency ablation. CAPOX=oxaliplatin and capecitabine chemotherapy. 5-FU=fluorouracil. FOLFOX=leucovorin, fluorouracil, and oxaliplatin. FOLFIRI=leucovorin, fluorouracil, and irinotecan. GORD=gastro-oesophageal reflux disease. NIDD=non-insulin-dependent diabetes.

Part I interventions were of long duration (mean anaesthetic time 369·2 min [SD 38·4]) because of time spent simultaneously localising the target tumour with diagnostic ultrasound by two different intercostal approaches and optimising the focused ultrasound parameters with thermometry feedback before drug infusion.^13^ The procedurally simpler part II interventions, which did not require the insertion of an intratumoural thermometry device and real-time optimisation of focused ultrasound parameters, were expectedly of shorter duration (mean anaesthetic time 213·8 min [SD 21·0]) (appendix p 3). For focused ultrasound, we used ultrasound powers in the range of 50–140 watts (corresponding to estimated in-situ peak rarefactional pressures in the range of 5·0–8·2 MPa) and duty cycles between 30% and 100%, depending on tumour location (appendix p 2).

In part I of the study (n=6 patients), the mean intratumoural biopsy doxorubicin concentration post-LTLD plus focused ultrasound was 7·74 μg/g (SD 4·09), representing an increase of 3·3 times compared with the mean post-LTLD concentration of 2·34 μg/g (0·93; table 2, appendix pp 7, 8). In all cases, doxorubicin was not detected in tumour samples taken before drug infusion, while higher doxorubicin concentrations were detected in samples taken post-LTLD plus focused ultrasound, compared with those of post-LTLD samples (table 2).

**Table 2:** High-performance liquid chromatography (HPLC) biopsy results after analysis of chromatograms

	**Biopsy mass, mg (minutes after end of infusion)**			**Total intratumoural biopsy doxorubicin concentration (μg/g), normalised for mass**			**Times by which doxorubicin concentration increased post-LTLD + FUS *vs* post-LTLD alone**
	Pre-LTLD	Post-LTLD	Post-LTLD + FUS	Pre-LTLD	Post-LTLD	Post-LTLD+FUS	
I.01	2·78	2·57 (2)	6·64 (56)	0·0	2·56	5·32	2·1
I.02	2·19	2·81 (0)	4·85 (81)	0·0	1·78*	13·20†	7·4
I.03	3·11	2·10 (5)	3·94 (93)	0·0	4·09	7·89	1·9
I.04	1·59	2·31 (3)	2·77 (74)	0·0	1·59	2·09	1·3
I.05	0·84	2·73 (1)	1·30 (90)	0·0	2·23	11·50	5·2
I.06	1·76	1·40 (4)	3·97 (87)	0·0	1·79*	6·41	3·6
II.01	*..*	*..*	2·45 (96)	*..*	*..*	6·65	2·8
II.02	*..*	*..*	2·42 (114)	*..*	*..*	6·84	2·9
II.03	*..*	*..*	2·49 (89)	*..*	*..*	3·89	1·7
II.04	*..*	*..*	2·91 (90)	*..*	*..*	21·80	9·3
Mean	2·05	2·32 (2·5)	3·37 (87·0)	0·0	2·34	8·56	3·7
SD	0·83	0·52 (1·9)	1·53 (15·0)	0·0	0·93	5·69	2·7

Mean total concentration of intratumoural biopsy doxorubicin after lyso-thermosensitive liposomal doxorubicin (LTLD) treatment in six patients in part I was used as a comparator for part II.

* Responses (ratio of doxorubicin to internal standard) were significantly lower than the response seen for the plasma lower limit of quantification (LLOQ; 0·1 μg/mL for I.02 and 0·05 μg/mL for I.06); the values shown are upper-bound estimates; assuming that the response for these samples would be the same as that for the LLOQ, the amounts are calculated for the mass of each specific biopsy analysed. Because the values are upper-bound estimates of the possible concentration, the part I post-LTLD mean comparator is probably an overestimate of the true concentration.

† Estimate only, because the internal standard was inadvertently omitted from this sample during processing.

**Figure 2:**
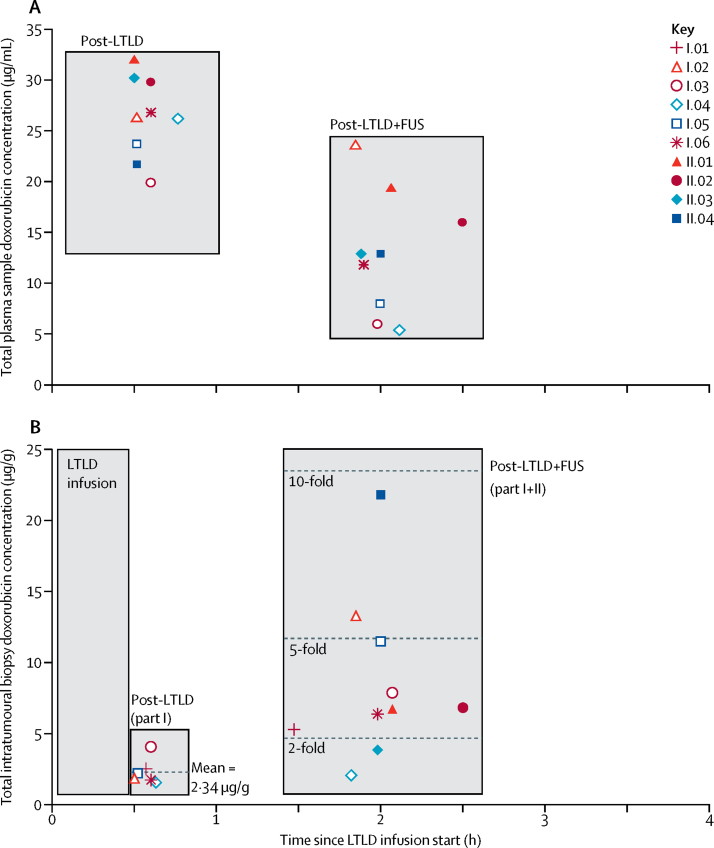
Total doxorubicin concentration in plasma and tumour samples analysed by high-performance liquid chromatography (HPLC) (A) Lyso-thermosensitive liposomal doxorubicin (LTLD) plasma pharmacokinetic data by HPLC. Data for patient I.01 are omitted from the plot, because concentrations were much greater than the top standard, resulting in a ten-fold dilution step for plasma analysis subsequently being introduced to the assay. (B) Intratumoural pharmacokinetic data by HPLC. The post-LTLD values for patient I.02 and I.06, and the post-LTLD plus focused ultrasound (FUS) values for patient I.06 are worst-case estimates.

Overall, the mean doxorubicin concentrations in post-LTLD plus focused ultrasound intratumoural biopsy samples from all ten study participants was 8·56 μg/g (SD 5·69)—an increase of 3·7 times compared with the mean intratumoural biopsy concentrations in part I post-LTLD samples (table 2, figure 2). The highest intratumoural drug delivery was detected in a colorectal metastatic target tumour, in a patient treated in part II without thermometry monitoring, in which the intratumoural doxorubicin concentration, based on biopsy estimate, reached 21·8 μg/g, suggesting an increase of nearly 10 times.

Of part I patients, at least a doubling in intratumoural doxorubicin concentration was seen post-LTLD plus focused ultrasound compared with post-LTLD alone in all patients apart from patients I.03 and I.04 (figure 2), with patient I.03 also being the only one in whom sustained hyperthermia of 39·5°C or higher was not recorded (table 3). Of the part II patients, patient II.03 was the only patient under the two-times increase limit required to satisfy the primary endpoint (figure 2). Given that seven (70%) of ten patients showed at least a doubling increase in intratumoural biopsy doxorubicin concentrations after focused ultrasound exposure, with concentrations from biopsies obtained in part II compared with the mean concentration post-LTLD alone from biopsies obtained in part I, our study met its primary endpoint (table 2). This analysis included three estimated datapoints, all of which made the primary endpoint harder to attain. The post-LTLD intratumoural doxorubicin concentrations for patients I.02 and I.06 were upper-bound estimates that were required because of low quantification concentrations (further detailed in table 2). The post-LTLD plus focused ultrasound intratumoural doxorubicin concentration estimate for patient I.06 was required because of the inadvertent omission of the internal standard during processing. HPLC data were recalculated for samples from patients I.01 and I.02 with standard curves created without the use of internal standard area for biopsy and plasma samples. This alternative quantification method yielded accuracy and precision of quality controls within 15% for both methods of analysis. Plasma concentrations calculated with this method were similar for both methods of quantification; however, the calculated biopsy concentrations were lower when the internal standard was excluded in calculations compared with when it was included. This difference was mainly because of lower recovery of the anthracyclines in tissue samples when compared with plasma samples. Therefore, the estimated post-LTLD plus focused ultrasound value for intratumoural doxorubicin in patient I.02 is likely to represent an underestimate of the true concentration.

**Table 3:** Summary of statistics for the post-drug thermometry analysis for all part I patients, for both raw and polynomial fitted data

	**Real-time trace duration (min:s)**	**Analysis of unprocessed real-time thermometry data**			**Thermometry analysis of polynomial fitted real-time thermometry data**			
		Temperature 39·5–47°C (min:s)	Temperature >47°C (min:s)	Mean temperature, °C (SD)	Maximum temperature, °C	Minimum temperature, °C	Mean temperature, °C (SD)	CEM_43_(min:s)
I.01	33:13	23:19	00:00	39·8 (0·77)	40·4	37·1	39·7 (0·81)	00:28
I.02	74:34	27:52	00:00	39·4 (1·66)	41·4	35·9	39·3 (1·30)	01:42
I.03	73:54	00:06	00:00	38·9 (0·37)	39·2	38·0	38·9 (0·36)	00:16
I.04	65:59	63:14	00:00	41·5 (0·95)	42·6	37·4	41·5 (1·07)	13:05
I.05	79:52	55:49	00:00	40·1 (1·25)	41·1	37·4	40·0 (0·82)	02:07
I.06	64:11	35:57	02:00	40·7 (2·58)	41·4	39·1	40·6 (0·47)	02:52

Cumulative equivalent in minutes at 43°C (CEM_43_) has been calculated on the fitted data to better represent the average bulk temperature of the heated tumour volume and reduce thermistor artifact effects, which were especially prominent in patient I.06. Thermometry captured before or after focused ultrasound exposure has been excluded from the analysis. Polynomial fit was done with least squares fit of fourth order in MatLab.

**Figure 3:**
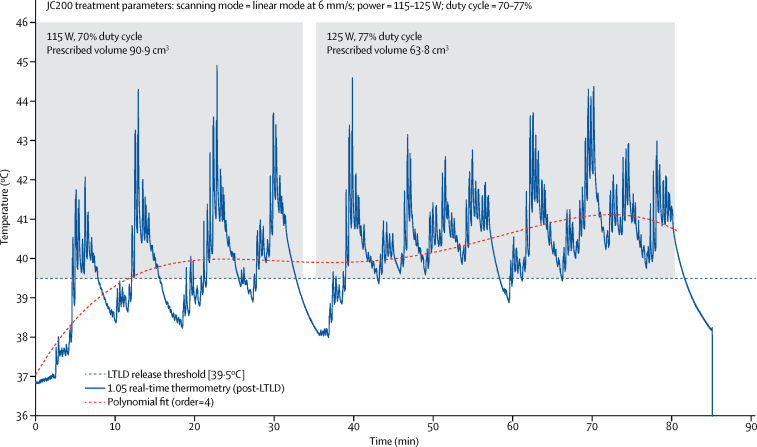
Illustrative controlled hyperthermia by focused ultrasound Real-time thermometry data (trace) captured after infusion of lyso-thermosensitive liposomal doxorubicin (LTLD) and during focused ultrasound exposure in moving beam (linear) mode for patient I.05. This trace was acquired at a 10 ms resolution by use of a calibrated Medtronic thermocouple, with custom LabView data-acquisition setup. Shaded regions represent the period when focused ultrasound was being applied. From approximately 30 s to 33 min, a 90·9 cm^3^ prescribed target tumour volume was exposed to focused ultrasound at 115 W (8·7 MPa peak rarefactional in situ pressure) at 70% duty cycle in linear mode. Although the release threshold was reached within 5 min of focused ultrasound exposure, heating in the first 30 min was deemed slightly suboptimal because of prolonged cooling periods between treatment cycles. Subsequently, by removing the outermost slices from the prescribed treatment volume, resulting in a smaller 68·3 cm^3^ tumour volume, and increasing power to 125 W (9·0 MPa derated) and duty cycle to 77%, optimal hyperthermia was achieved for 35–80 min. Once focused ultrasound stopped, the tumour was allowed to cool before the thermocouple was removed from the patient at 85 min, and a tumour biopsy sample was subsequently taken. The dotted curve is a fourth order polynomial fit, which is probably more representative of the bulk temperature in the prescribed tumour volume than the rapidly fluctuating point temperature recorded by the sensitive region of the intratumoural thermometry device (trace).

Six patients from part I, with prescribed tumour volume range of 10·5–73·4 cm^3^ (mean 49·6 cm^3^ [SD 26·3]), and four from part II, with prescribed tumour volume range of 30·7–53·9 cm^3^ (43·8 cm^3^ [10·3]), were exposed to focused ultrasound (overall mean tumour volume 47·3 cm^3^ [20·7]; appendix p 2). Sustained and controlled hyperthermia (>39·5°C) was achieved in five (83%) of six part I patients for a mean of 40·8 min (SD 15·7). In these six patients, mean intratumoural temperatures of 38·9–41·5°C (overall mean 40·1°C, SD 0·9) were recorded between 33 min and 79 min (mean 65·3 min, 16·7; table 3, figure 3, appendix pp 9–11). In the one patient (I.03), in whom hyperthermia of 39·5°C or higher was achieved momentarily but not sustained (appendix p 10), gas introduced around the tip of the co-axial needle on introduction of the thermometry device (as visualised on the ultrasound-guidance B-mode imaging system) might have resulted in underrepresentation of the temperature in the target tumour volume. In one patient (I.06), spikes of intratumoural hyperthermia higher than 47°C for 2 min cumulatively were recorded (appendix p 11). These spikes were probably due to the thermocouple artifact caused by the incidental coincidence of the focused ultrasound beam with the thermocouple shaft, because these temperatures did not translate into bulk heating.^18^ For the remaining patients in part I (patients I.01 to I.05), real-time temperature measures did not exceed 46°C at any stage. Cumulative equivalent minutes at 43°C thermal dose analysis (table 3) is discussed in the appendix p 11.

Changes consistent with instantaneous tumour ablation, as is typically seen in high-intensity focused ultrasound thermal ablation,^19^ were absent on the diagnostic ultrasound guidance system (B-mode imaging) for all interventions. This absence of thermal ablation was further supported by the day 1 MRI findings, cell viability studies, and the predictive model (Gray MD, unpublished data). Some patients had an absence of MRI changes on day 1, followed by subsequent reductions in perfusion and PET-CT scans showing new photopenia at 2 weeks (patients I.04, I.06, and II.02), whereas others (patients I.01, I.05, and II.04) had reduced perfusion shown on day 1 MRI (appendix pp 20–27).

Specific cytokeratin-8 staining of viable tumour cells after drug and ultrasound exposure was seen in eight (89%) of nine patients whose biopsies yielded tissue analysable by microscopy, further supporting the absence of instantaneous thermal ablation due to direct effects of focused ultrasound (appendix pp 16–18).^20^ Specific tumour cell staining was not seen in the remaining analysed sample (of patient II.01), which was thought to be because of a non-cytokeratin-8 expressing non-small (squamous) cell lung metastasis, rather than ablative damage.

All patients were discharged the day after the intervention except patient II.04, who had a prolonged hospital stay because of fluctuating grade 1 confusion, which was classified as a serious adverse event, but deemed probably not related to the drug or intervention. Serious adverse events were otherwise restricted to self-resolving grade 4 neutropenia in five (50%) of ten patients, an expected adverse event of LTLD (table 4).^11^ Worsening of pre-existing liver function derangements (grade 3 or lower) was seen in all patients, except patient I.01, within the 30-day follow-up period (appendix p 3), in keeping with the known adverse event profile of LTLD.^21^ However, it was not possible to distinguish a drug-related cause of liver dysfunction from progressive liver malignancy with certainty in this patient cohort.

**Table 4:** All adverse events stratified by definite or probable causality

	**Grade 1-2**	**Grade 3**	**Grade 4**
**Definitely or probably related to LTLD: haematological toxic effects**			
Neutropenia or neutrophils decreased	0	1 (10%)	5 (50%)*
Anaemia	0	1 (10%)	0
Urinary tract infection	1 (10%)	0	0
**Definitely or probably related to LTLD: non-haematological toxic effects**			
Alopecia	8 (80%)	0	0
Candida infection	1 (10%)	0	0
Fatigue or lethargy	4 (40%)	0	0
Nausea	2 (20%)	0	0
Vomiting	2 (20%)	0	0
**Definitely or probably related to the procedure**			
Abdominal pain	2 (20%)	0	0
Back pain	1 (10%)	0	0
Decreased appetite	1 (10%)	0	0
Dysphonia	1 (10%)	0	0
Erythema	1 (10%)	0	0
Fatigue	1 (10%)	0	0
Hepatic pain	1 (10%)	0	0
Musculoskeletal chest pain	2 (20%)	0	0
Musculoskeletal pain or discomfort	6 (60%)	0	0
Nausea	1 (10%)	0	0
Pain in extremity	2 (20%)	0	0
Skin discolouration	1 (10%)	0	0
**Definitely or probably unrelated or possibly related to LTLD or procedure (indeterminate cause)**			
Abdominal pain	1 (10%)	0	0
Confusion state	1 (10%)*	0	0
Constipation	1 (10%)	0	0
Decreased appetite	2 (20%)	0	0
Fatigue	2 (20%)	0	0
Joint swelling	1 (10%)	0	0
Malaise	1 (10%)	0	0
Nausea	2 (20%)	0	0
Peripheral swelling	1 (10%)	0	0
Respiratory tract infection	1 (10%)	0	0
Vomiting	1 (10%)	0	0

No grade 5 adverse events occurred. Liver function tests pre-treatment and post-treatment are detailed in appendix p 3. LTLD=lyso-thermosensitive liposomal doxorubicin.

* Serious adverse events.

Non-invasive ultrasound-mediated hyperthermia was found to be safe, causing no skin burns, off-target tissue damage, or other clinically significant adverse events, either with or without real-time thermometry (table 4).

At the 2-week and 4-week follow-up visits, CT, MRI, and PET-CT scans were done. Patient I.02 was excluded from radiological analysis because of failed venous access, rendering post-treatment scans effectively non-contrast. For the remaining nine patients, targeted tumour volumes (exposed to both LTLD and focused ultrasound) were assessed with RECIST, Choi, and PERCIST criteria modified for a single target tumour and compared with control liver tumours (exposed to LTLD alone). A partial response according to Choi criteria was seen in the target tumour alone at 2 weeks and 4 weeks in patients I.01, I.04, I.06, and II.04, with patient II.04 also showing a partial response according to PERCIST criteria. In patients I.05 and II.02, a partial response according to Choi criteria was seen in both the target tumour and at least one control tumour, at 2 weeks and 4 weeks. All other target tumours (four [40%] of ten) and control tumours (13 [81%] of 16) assessed showed either stable or progressive disease according to RECIST, Choi, or PERCIST criteria at 2 weeks or 4 weeks (appendix pp 20–25). This targeted effect is well demonstrated in several patients: patient I.01, where the PET-CT showed new photopenia in the target tumour, whereas a similarly sized control tumour, incidentally captured in the same axial slice as the treated tumour, showed little or no response (appendix p 20); similarly, patients I.04, I.06, and II.04 only showed new photopenia in the region of the target tumour treated with ultrasound. Total lesion glycolysis of the target tumours was calculated at baseline and at 2 weeks (appendix p 6). The four patients with a partial response according to Choi criteria showed 36·4%, 22·6%, 18·2%, and 46·3% reductions in total lesion glycolysis, despite only having partial tumour coverage with focused ultrasound (appendix p 26).

After counterstaining of cell nuclei with 4',6-diamidino-2-phenylindole (DAPI), tumour biopsies were examined microscopically for evidence of intratumoural LTLD release within 2 months of sampling (appendix p 6). Tumour samples from patient I.01 were too auto-fluorescent for the doxorubicin signal to be distinguished from background noise in the paraffin-embedded samples, while the post-LTLD plus focused ultrasound tissue sample from patient I.02 was contaminated with blood. Confocal fluorescence microscopy showed colocalisation of doxorubicin with the DAPI nuclear stain in seven (88%) of the remaining eight post-LTLD plus focused ultrasound tumour samples (patients I.04–06 and II.01–04; appendix pp 14–17). The presence of nuclear doxorubicin showed bioavailability of intratumoural doxorubicin and thus liposomal release, because doxorubicin must be in its free form to diffuse into the tumour cells.^22^ In the post-LTLD controls, minimal nuclear or extravascular doxorubicin was seen.

Plasma samples from each patient were assayed for total doxorubicin concentration by HPLC. The results for the first patient (I.01) were above the higher end of the standard curve; therefore, subsequent plasma samples of the remaining nine patients were diluted before analysis. For these patients, mean total plasma doxorubicin concentrations decreased from 26·3 μg/mL (SD 4·0) post-LTLD to 12·9 μg/mL (6·0) post-LTLD plus focused ultrasound. Results of the quenched and dequenched fluorometric plasma analysis are available in the appendix (pp 12, 13).

## Discussion

To our knowledge, our study was the first to attempt non-invasive ultrasound-mediated targeted hyperthermia for triggered drug release and enhanced drug delivery in a clinical setting. Apart from the existing risks associated with general anaesthesia, the overall intervention posed no additional safety concerns to the patient other than those typically associated with chemotherapy alone. Mean temperatures needed for drug release were safely maintained in clinically relevant tumour volumes by extracorporeal focused ultrasound, for about 1 h, without subsequent radiological or histological evidence of thermal ablation. This approach resulted in a substantial increase in the total intratumoural biopsy concentration of doxorubicin, compared with that achieved by passive accumulation alone, with seven of ten patients having at least a doubling and an increase of up to ten times in intratumoural doxorubicin concentrations. This increase occurred concurrently with the presence of nuclear doxorubicin, showing liposomal release after focused ultrasound exposure, and localised radiological responses in the target tumour regions exposed to focused ultrasound in several patients. Overall, these findings suggest that target drug delivery to solid tumours triggered non-invasively by therapeutic ultrasound is clinically safe, feasible, and potentially effective.

The non-thermosensitive liposomal formulation of doxorubicin, NTLD, is hypothesised to exert its therapeutic effect predominantly by the enhanced permeability and retention effect.^23^ By contrast, the LTLD formulation that we used in this trial is understood to act by rapid diffusion of doxorubicin into the tumour interstitium under conditions of mild hyperthermia, with only a small proportion of doxorubicin entering the tumour by enhanced permeability and retention. These intended delivery mechanisms are reflected in the circulation profiles of the two formulations, with NTLD having a half-life of more than 10 h while LTLD has a shorter half-life of about 1 h.^9^, ^10^, ^24^, ^25^

Mild hyperthermia has been shown to enhance the perfusion and increase the permeability of tumour vasculature.^26^ Because focused ultrasound exposure begins while blood concentrations of LTLD are at their peak^11^, ^13^ (figure 2) and at a timepoint before any substantial enhanced permeability and retention-assisted accumulation could have occurred, it is our understanding that the majority of the intratumoural doxorubicin is released from circulating LTLD in the tumour microvasculature, when the tumour reaches the liposomal transition temperature (39·5°C). Free (bio-available) circulating doxorubicin might then rapidly diffuse into the adjacent or downstream perivascular space, and on to tumour cells at therapeutic concentrations and penetration depths due to a steep concentration gradient, surpassing what could be achieved through passive circulation alone.

The post-LTLD intratumoural biopsy concentrations of doxorubicin (after infusion) were similar to those seen in preclinical tumour models of LTLD.^5^ Differences in the measured total doxorubicin concentrations before focused ultrasound exposure were very small across patients. In keeping with preclinical studies,^5^, ^7^ this result strongly suggests that there is little opportunity for passive accumulation of LTLD in the first 2 h after administration, but there could still be differences in the leakiness of tumour vascular pores across the different tumour subtypes treated.^27^ The much greater differences in total doxorubicin concentration observed after focused ultrasound exposure are probably due to a combination of factors, including the differences in tumour leakiness across subtypes, tumour heterogeneity, the percentage of the ultrasonically treated volume that was adequately vascularised, and the total duration of focused ultrasound-mediated hyperthermia relative to the patient-specific pharmacokinetics of LTLD. Despite the use of a different analytical technique, pharmacokinetic profiles for total doxorubicin were similar to previously published clinical data,^11^ in which the same dose of LTLD was combined with radiofrequency ablation of liver tumours.

Magnetic resonance spectroscopy, particularly chemical exchange saturation transfer, was explored as a method of quantifying free doxorubicin preclinically, using phantom and small animal studies (unpublished). We had initially intended to do similar analyses clinically during the day 1 MRI sequences of our trial. However, the signal-to-noise ratio of this approach was ultimately deemed too poor for clinical translation, and this research avenue was not pursued.

The absence of MRI changes on day 1, followed by subsequent reductions in perfusion and PET-CT scans showing new photopenia at 2 weeks in some patients (I.04, I.06, and II.02), suggests a delayed chemo-ablative response rather than direct instantaneous thermal ablation (appendix pp 19–27). Reduced perfusion shown on day 1 MRI in other patients (patients I.01, I.05, and II.04) might be due to temporary vasoconstriction, tumour thrombosis, or a possible early tumour response.

The direct cytotoxic effects of doxorubicin have long been thought to be enhanced under hyperthermic conditions, a hypothesis based on increased cellular uptake and mechanisms relating to inhibition of nucleic acid synthesis and DNA repair.^28^ However, extensive preclinical work before this study showed that addition of ultrasound-mediated hyperthermia to free doxorubicin, at modest thermal doses used in our study, appears to have little effect on intratumoural accumulation and therapeutic response.^5^, ^7^ This finding suggests that the enhancement in delivery observed in this study is probably related to the sudden increase in concentration triggered by the release of doxorubicin from thermosensitive liposomes in the tumour microvasculature, rather than to any hyperthermia-mediated chemosensitisation alone.

In this early study, it remains unclear whether ultrasound-mediated cavitation is involved in the mechanism of delivery, but preclinical data strongly suggest that the microstreaming and shockwaves created by cavitation can be a benefit to the delivery of cancer drugs.^29^, ^30^

Although our phase 1 study was, to the best of our knowledge, the first to translate focused ultrasound-triggered targeted drug delivery to a clinical oncology setting, the study design had inherent limitations. First, the prescribed tumour treatment volumes were constrained by the presence of ribs in the acoustic field (which restricted the real-time B-mode treatment monitoring and guidance and reduced the focused ultrasound beam intensity) and by the focused ultrasound system itself (with restricted scanning capabilities and a nominally small, fixed ultrasonic focus), such that, typically, only partial sonication of larger tumours could be achieved, usually including tumour borders closest to the ultrasound source. Custom focused ultrasound devices designed for volumetric hyperthermia—for example, devices using less tightly focused transducers or multi-element systems with beam steering^8^—might allow rapid induction of large volume hyperthermia, facilitating the treatment of larger tumour volumes with this strategy.

Second, tumours are highly heterogeneous and show great microregional variations, not least in the amount of vascularity and necrosis.^31^ In this study design, tumour biopsies were intended to provide an estimate of the mean intratumoural drug concentration in the focused ultrasound-exposed tumour volume. However, the total intratumoural drug concentration reported served as a point measurement that was not necessarily reflective of the mean concentration throughout the tumour. This design limitation is mitigated, to some extent, by subsequent radiological analysis of the targeted tumours, most crucially by PET-CT scan, a technique that can discriminate metabolically active from necrotic or chemo-ablated tumours.

Third, the study design did not include time-matched control biopsies from liver tumours receiving LTLD alone, because doing more than three sequential tissue biopsies in more than one location in any one patient was neither ethical nor practical.^13^ Preclinical studies of LTLD showed that, in great part because of its short half-life (1 h compared with more than 10 h for NTLD), the additional intratumoural accumulation beyond 30 min after administration is not substantial, and falls well short of the doubling increase in intratumoural concentration that constituted the primary endpoint of our study.^6^, ^8^ Radiological follow-up enabled indirect assessment of negligible longer-term passive accumulation of LTLD.

Fourth, although the HPLC tumour results were generated by a validated method, worst-case estimations were done in three cases. However, since these estimates were worst-case estimates, we are confident that the primary endpoint is not compromised.

A fifth limitation was that the introduction of any instrumentation into the target tumour risked contamination of tumour samples with blood products. Haemotoxylin and eosin staining was done on a section of the same cores used for HPLC assessment. The post-LTLD sample for patient I.04 and the post-LTLD plus focused ultrasound sample for patient I.02 were both shown to contain blood contamination on haemotoxylin and eosin staining. Therefore, although we do not know the exact tissue constituents that were homogenised for HPLC, these individual pharmacokinetic tumour results should be interpreted with caution. Nevertheless, exclusion of these two results from the overall analysis does not alter the outcome of the study, because seven of eight patients still had an increase greater than doubling in drug concentrations and the study still met its primary endpoint.

Lastly, a magnetic resonance spectroscopy-guided focused ultrasound system would have provided valuable spatial thermography and coregistration with the follow-up MRI, facilitating both treatment and response assessment. However, the ultrasound-guided focused ultrasound device used made practical issues surrounding the use of high-frequency jet ventilation anaesthesia and the biopsy procedure less challenging because of the absence of a magnetic field.

By design, part II of the study proceeded without invasive thermometry, and this was shown to not adversely affect the efficacy of the intervention; indeed, the mean post-LTLD plus focused ultrasound intratumoural concentration of doxorubin in part II was 9·80 ug/g, compared with 7·74 ug/g in part I.

Whether delivering small molecules, antibodies, or viruses, maximising the dose delivered to a tumour while minimising off-target toxicity represents a universal challenge in oncology.^32^ A major limitation of systemically administered liposomal agents is their low therapeutic index: the dose required to produce a successful antitumour effect is toxic to normal tissues. The antitumour activity of traditional liposomal agents is hindered by a failure to effectively release their cargo and an inability to penetrate beyond the perivascular space. Device-targeted drug delivery has undergone decades of preclinical development but could be nearing clinical adoption as a generic tool for overcoming the challenges of delivering existing and emerging therapeutics to solid tumours. MRI-based or ultrasound-based treatment monitoring strategies—or detailed predictive treatment planning, as was used in this study—are likely to facilitate clinical adoption for non-invasive targeting. Preclinical MRI studies^33^, ^34^ have combined three-dimensional thermography with sophisticated closed-loop feedback algorithms at high temporal resolutions to reduce microregional temperature fluctuations, and a clinical study involving the use of magnetic resonance-guided high intensity focused ultrasound with LTLD is underway for treatment of paediatric solid tumours (NCT02536183). By using an ultrasound-guided focused ultrasound device without thermography, our study explored the economically attractive and more easily scalable alternative than MR-guided techniques for attaining large-volume bulk hyperthermia with predictive models, with less emphasis on maintaining tight and homogeneous temperature control.

Overall, this prospective study shows for the first time in a clinical setting the safety, feasibility, and potential for therapeutic benefit of ultrasound-triggered release of LTLD in otherwise chemorefractory tumours. The small sample size of ten patients reflects that this trial was a proof-of-concept study, for which participation was predominately altruistic, involving only a single cycle of chemotherapy and targeting of a single tumour. The tumours targeted on this study were typically large, of diverse histological type, and were refractory to systemic chemotherapy. A demonstrable radiological response in the targeted tumour volume alone is encouraging, given that doxorubicin has been previously shown to have reduced therapeutic value in many of these tumour subtypes.^21^, ^35^ Despite its limitations, our study shows for the first time in a clinical setting that it is feasible to safely trigger and enhance intratumoural delivery of a chemotherapeutic agent to a precise anatomical location at depth, by using focused ultrasound applied non-invasively. Further preclinical and clinical research in focused ultrasound-mediated drug delivery might be warranted, with the intention of reducing toxicity and improving therapeutic outcomes in a broad range of solid tumours, potentially across multiple drug classes.
