# Supplementary material for: Safety and feasibility of ultrasound-triggered targeted drug delivery of doxorubicin from thermosensitive liposomes in liver tumours (TARDOX): a single-centre, open-label, phase 1 trial
Source: Lancet Oncol. 2018 Aug;19(8):1027–39. doi: 10.1016/S1470-2045(18)30332-2 (PMC6073884; doi:10.1016/S1470-2045(18)30332-2)
Supplement: Supplementary appendix [file mmc1.pdf]

THE LANCET
Oncology

Supplementary appendix

This appendix formed part of the original submission and has been peer reviewed. We post it as supplied by the authors.

Supplement to: Lyon P C, Gray M D, Mannaris C, et al. Safety and feasibility of ultrasound-triggered targeted drug delivery of doxorubicin from thermosensitive liposomes in liver tumours (TARDOX): a single-centre, open-label, phase 1 trial. *Lancet Oncol* 2018; published online July 9. http://dx.doi.org/10.1016/S1470-2045(18)30332-2.

TARDOX WEB APPENDIX: A Phase I trial investigating the safety and feasibility of ultrasound-triggered targeted drug delivery in liver tumours

SUPPLEMENTARY CLINICAL INFORMATION 2

TUMOUR CHARACTERISTICS & FOCUSED ULTRASOUND EXPOSURE PARAMETERS 2
ANAESTHETIC & LTLD INFUSION TIMES 3
ADVERSE EVENT DATA: LIVER FUNCTION TESTS 3

DETAILED METHODS 4

PROTOCOL SUMMARY 4
INTERVENTION 4
SAMPLE HANDLING 5
QUANTIFICATION OF DOXORUBICIN BY HPLC WITH FLUORESCENCE DETECTION 5
EVALUATION OF ENCAPSULATED PLASMA DOXORUBICIN BY FLUOROMETRY 5
MICROSCOPIC ANALYSIS 6
CALCULATION OF TOTAL LESION GLYCOLYSIS 6

ADDITIONAL CLINICAL RESULTS: LABORATORY 6

HPLC ESTIMATIONS FOR BIOPSY SAMPLES 6
HPLC CHROMATOGRAMS FOR BIOPSY SAMPLES 7
PART I REAL-TIME THERMOMETRY 9
CEM43 THERMAL DOSE CALCULATION 11
HPLC ANALYSIS OF PLASMA SAMPLES 12
EVALUATION OF ENCAPSULATED PLASMA DOXORUBICIN BY FLUOROMETRY 12
DIRECT FLUOROMETRIC ANALYSIS OF UNHEATED AND HEATED PLASMA SAMPLES 12
PLASMA PHARMACOKINETICS BY DEQUENCHING ASSAY 13
MICROSCOPIC DRUG DELIVERY ANALYSIS FOR I.04 14
MICROSCOPIC DRUG DELIVERY ANALYSIS FOR I.05 15
MICROSCOPIC DRUG DELIVERY AND CELL VIABILITY ANALYSIS FOR I.06 16
MICROSCOPIC DRUG DELIVERY AND CELL VIABILITY ANALYSIS FOR II.04 17
MICROSCOPIC CELL VIABILITY ANALYSIS FOR I.05 18

ADDITIONAL CLINICAL RESULTS: RADIOLOGY 19

RADIOLOGY FIGURES 19
PET-CT GLYCOLYTIC VOLUME ANALYSIS 26
DAY 1 MRI PERFUSION ANALYSIS 26
CORRELATION OF MRI PERFUSION WITH PET-CT 27

REFERENCES 28

FULL STUDY PROTOCOL 28

Supplementary Clinical Information

Tumour Characteristics & Focused Ultrasound Exposure Parameters

Patient ID	Liver Segment	Patient Position During FUS Exposure	Approximate Tumour Metrics by Baseline MRI				Prescribed Target Volume (cm^3)	Estimated % of Tumour Volume Treated	Mean Acoustic Powers (W)	Estimated Peak Rarefactional In Situ Pressure (MPa)	Duty Cycle (%)
			Tumour Size (mm)	Depth from Skin to Superficial Tumour Border in FUS Direction (mm)	Depth from Skin to Deepest Tumour Border in FUS Direction (mm)	Estimated Tumour Volume (cm^3)					
I.01	VI	Supine	35x30x25	35	58	13·7	10·5	76·6	50	6·7	100
I.02	V/VI	Supine	62x58x47	48	124	88·4	25·0[1]	28·3	70	5·6	100
I.03	V/VI	Supine	70x70x58	80	133	148·8	73·2	49·2	100	5·0	100
I.04	V/VI	Supine	62x48x47	38	87	72·2	73·4	100·0	140	7.7	30-50
I.05	VI	Supine	66x48x40	36	79	66·4	90·9, 63·8	100·0, 96·1	115, 125	7·2, 7·5	70, 77
I.06	VI	RLD + 30° tilt to supine	43x38x31	25	58	26·5	51·5[2]	100.0	116	8·2	43
II.01	VI	RLD	37x33x29	52	90	18·5	40·7[3]	100.0	123	7·9	55
II.02	V	Supine + 30° tilt to RLD	98x70x58	26	56	43.0	49·9	100.0	114	8·0	42
II.03	VIII	RLD	88x76x64	56	86	224·1	30·7	7.3	140	7·9	44
II.04	VIII	RLD	55x52x38	27	67	56·9	53·9	94.7	110	8·0	40

Supplementary Table 1: Characteristics of the ultrasound-targeted liver tumour with FUS exposure parameters, for each patient receiving intervention. Estimated tumour volume was calculated using the ellipsoid volume rule. RLD = Right lateral decubitus. The prescribed volume reported by the therapeutic FUS device refers to the volume bounded by user-defined coordinates, used to generate FUS exposure plans. Note that where multiple treatment exposure cycles were used to sustain hyperthermia, the actual treated volume will naturally be multiples of the prescribed volume. The peak rarefactional in situ pressures refer to the maximal negative tissue pressures experienced at the centre of the target volume, corrected for attenuation of the soft tissues in the incident path (in situ pressures de-rated at 0·6 dB/cm/MHz), as estimated by a non-linear model. For patient I.05, the prescribed volume was reduced during the FUS exposure, see Figure 4 for annotated thermometry trace.

1 The prescribed volume for I.02 is an estimate, as it was not recorded at treatment time and to our knowledge, cannot be obtained retrospectively from the operating software.

2 The prescribed volume is considerably larger than the estimated volume of the target tumour. This is because the target tumour was beginning to coalesce with an adjacent tumour at the time of treatment, and consequently had indistinct borders on ultrasound, and so part of the adjoining tumour was also included in the treatment volume (refer to PET-CT, Supplementary Figure 10(b)).

3 The prescribed volume is larger than the estimated volume of the target tumour. This is because in this patient, a margin of normal liver was included in the treated volume in an attempt to ensure full coverage of the target tumour in this patient with oligometastatic liver disease (refer to PET-CT, Supplementary Figure 10(b)).

Anaesthetic & LTLD Infusion Times

	Patient ID	Anaesthetic Time Before Drug Infusion (minutes)	Drug Infusion Time (minutes)	Anaesthetic Time After Drug Infusion (minutes)	Total Anaesthetic Time (minutes)
**Part I**	**I.01**	332	32	65	429
	**I.02**	251	30	96	377
	**I.03**	239	24	92	355
	**I.04**	193	35	82	310
	**I.05**	232	30	110	372
	**I.06**	241	32	99	372
**Part II**	**II.01**	63	28	114	205
	**II.02**	150	24	119	245
	**II.03**	76	30	94	200
	**II.04**	75	30	100	205
**All Cases**	**Mean**	185·2	29·5	97·1	307·0
	**SD**	90·9	3·4	15·8	86·1
**Part I**	**Mean**	248·0	30·5	90·7	369·2
	**SD**	45·8	3·7	15·5	38·4
**Part II**	**Mean**	91·0	28·0	106·8	213·8
	**SD**	39·8	2·8	11·7	21·0

Supplementary Table 2: Summary of anaesthetic and LTLD infusion timings. Part II cases were considerable shorter due to the simplified procedural steps required for intervention.

Adverse Event Data: Liver Function Tests

Patient ID	Baseline LFTs (within 1-week pre-treatment)					Follow-up LFTs (within 30 days post-treatment)				
	Bili	ALT	AST	ALP	Alb	Highest Bili	Highest ALT	Highest AST	Highest ALP	Lowest Alb
I.01	9	18	23	115	35	13	28	26	127	30
I.02	14	32	96	404	38	16	111	148	590	32
I.03	23	11	14	90	33	21	25	74	209	30
I.04	6	36	43	231	36	5	70	61	410	29
I.05	18	46	45	383	28	25	61	108	790	23
I.06	4	68	66	523	33	10	116	272	616	30
II.01	6	73	65	123	31	11	109	93	123	30
II.02	7	50	31	163	32	3	80	57	209	31
II.03	8	63	57	253	28	7	52	41	298	24
II.04	6	17	24	221	32	7	17	22	278	32

Supplementary Table 3: Liver function tests (LFTs) pre- and post-treatment. Bili=Bilirubin (normal: 0-21 μmol/L), ALT=Alanine aminotransferase (normal range: 10-45 IU/L), AST=Aspartate aminotransferase (normal range: 15-42 IU/L), ALP=Alkaline phosphatase (normal range: 30-130 IU/L), Alb=Albumin (normal range: 32-50 g/L).

Detailed Methods

Protocol Summary

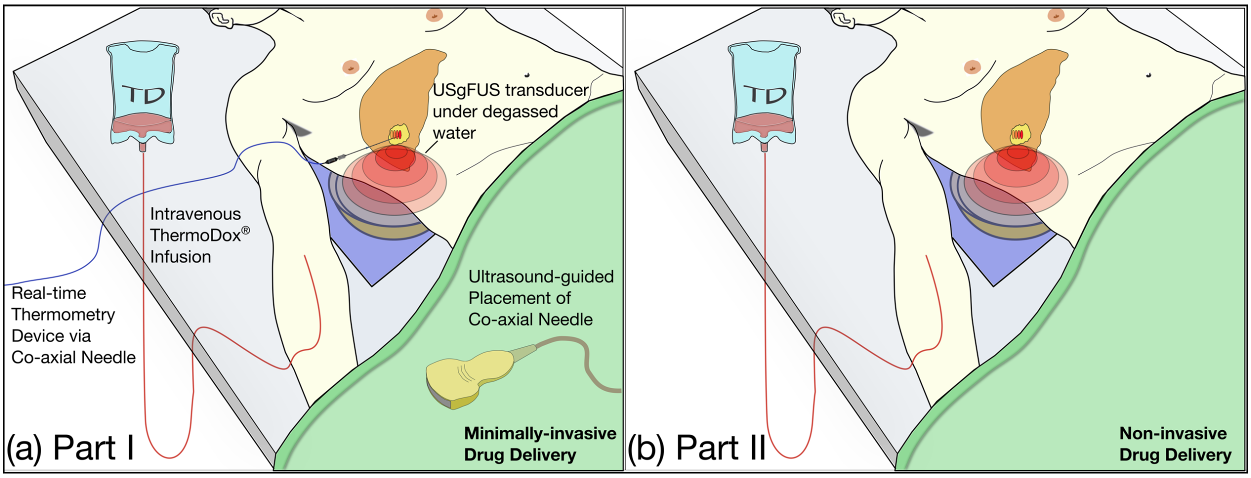

Supplementary Figure 1: Protocol summary. Schematics of the study interventions, highlighting the key differences between Part I (a) and Part II (b). The patient laid over the degassed water bath of the FUS device (JC200), containing an ultrasound-guided focused ultrasound (USgFUS) therapeutic transducer, such that the focus of the transducer was aligned with the target tumour through the relevant intercostal space. (a) Part I of the study involved insertion of a co-axial needle into the target liver tumour under ultrasound guidance. Patient positioning was modified to facilitate an additional intercostal ultrasound approach to the target tumour using a hand-held diagnostic probe, to allow for co-axial needle insertion above the level of the integrated water bath through which the therapeutic transducer transmits. This needle was used to take core biopsies of the tumour before and immediately after LTLD infusion, and finally after FUS-mediated delivery. In addition, the co-axial needle was used to pass a clinically approved thermometry device during FUS exposure, for real-time thermometry. (b) Part II did not utilise a thermometry device, and FUS is applied to the target tumour following LTLD infusion. Targeted drug delivery was thus performed completely non-invasively. Two serial core biopsies of the target tumour were taken following the FUS exposure. Part II afforded more flexibility in patient positioning, and the supine position shown is illustrative only.

Intervention

On the day prior to treatment, baseline scans (perfusion CT and dynamic contrast-enhanced MRI of the liver, ^{18}F-FDG PET-CT) were performed and the required pre-medications (steroids) were given (see clinical protocol, Appendix).

On the day of intervention, patients were positioned typically supine or right lateral decubitus on the clinical FUS device to enable visualization of the target tumour using the on-board diagnostic ultrasound probe through cooled, degassed water, between an intercostal space (Supplementary Figure 1). Treatment was performed under general anaesthesia using high-frequency jet ventilation to minimize liver movement.[1]

LTLD was infused over a 30-minute period into a peripheral vein, at the maximum tolerated dose of 50 mg/m^2.[2] The target tumour volume, of characteristic dimensions 3-5 cm and typically smaller than the tumour itself, was segmented into treatment planes using the real-time B-mode ultrasound imaging from a probe concentrically located within the annular FUS source. The motorized positioning system of the FUS device was then used to translate the FUS source focus (and integrated B-mode probe) through each plane automatically, using either single shot (dot mode) or continuous (linear mode) sonication. FUS was delivered to alternating non-adjacent planes for approximately 30-60 minutes in real-time, in order to both induce and sustain hyperthermia throughout the target volume. B-mode images were available throughout the treatment to check for signs of hyperechoic changes – an indicator of unwanted bubble formation which would effectively indicate tissue boiling due to cavitation.

During intervention, venous blood samples were taken for pharmacokinetic analysis at three time points: pre- and post-infusion of LTLD, and following FUS. For Part I patients, core biopsies of the target liver tumour volume were taken synchronously with these three blood samples. For Part II patients, the target tumour volume was only

biopsied under ultrasound guidance after FUS, through a newly inserted co-axial needle (such that the drug delivery phase was performed completely non-invasively). Tissue biopsies were obtained using an 18-gauge needle, each of an anticipated mass of 4-5 mg.[3] Following the final post-LTLD+FUS biopsy, the anaesthetic was reversed, and patients were monitored on the recovery unit before being stepped down to the oncology ward.

Sample Handling

Tumour cores were immediately divided into two parts with a dedicated scalpel; one was placed in a pre-weighed tube on ice, protected from light. Within 3 hours of collection the tube was re-weighed and then frozen at -80 °C for subsequent drug quantification by high performance liquid chromatography (HPLC). The remaining biopsy part was formalin-fixed the same day and subsequently paraffin-embedded for haematoxylin and eosin (H&E) and confocal fluorescence microscopy for both drug delivery studies and cell viability. Venous samples were collected in serum separation tubes, kept on ice whilst protected from light, and plasma was spun off within 3 hours of draw. An aliquot of each plasma sample was frozen at -80 °C for subsequent analysis by HPLC and the remainder was used for same day heated (dequenched) and unheated (quenched) fluorometric analysis (see Supplementary Material for methodology).

Quantification of Doxorubicin by HPLC with Fluorescence Detection

A validated assay was developed, using daunorubicin as an internal standard (IS), for the analysis of doxorubicin in plasma with a lower limit of quantification (LLOQ) of 0·05 µg/mL in plasma (0·1 µg/mL for I.01 and I.02). A calibration curve in plasma was established to reflect the expected concentrations of doxorubicin in the tissue samples; the concentrations measured (µg of drug per g of tissue) were dependent upon the total mass of tissue which was extracted (expected to be no greater than 5 mg for a section of an 18-guage tumour biopsy).[3] Liver tumour biopsy samples and plasma samples collected from patients who received the intervention were analysed alongside the same plasma calibration curve in the same analytical run. For this reason, plasma samples were diluted 10-fold in control plasma to enable concentrations to fall within the span of the calibration curve (0·05-5·00 µg/mL). Diluted plasma (50 µL) was mixed with IS and both anthracyclines were extracted using liquid-liquid extraction with acetone and zinc sulphate solution with high dose recovery, similar to that used in previous pre-clinical studies.[4,5]

For tumour samples, after addition of 50 µL deionised water and IS to a pre-weighed vial containing the tumour tissue (making the total biopsy volume), and tissue homogenization by use of a dismembrator (Mikro-dismembrator U, Braun Biotech Int.) biopsies were processed in the same way as for plasma. Biopsy weight and a dilution factor of 50 were used by the HPLC software (Empower 2) to calculate the concentration of doxorubicin in µg/g tissue.

The ratio of the area under the curve for doxorubicin relative to the IS was used to generate a calibration curve for determination of sample concentrations. Chromatography utilized a Waters (Watford, UK) 2695 separations Module and Waters 474 fluorescence detector at excitation wavelength of 480 nm and emission of 560 nm. Separation was achieved using a Phenomenex C6-Phenyl guard and analytical column (3 µm, 3 x 150 mm) at 35 °C with a flow rate of 0·5 mL/min with 10 mM formic acid (aqueous phase) and acetonitrile (organic phase) using an elution gradient (I.03-I.06 and II.01-II.04) increasing from 12-40 % of organic phase in 11 min, then 40-80% organic phase in 1 min with a 2 min hold then returning to starting conditions at 14 min. For I.01 and I.02, chromatography consisted of an elution gradient of 22-40% of organic phase in 6 min, returning to starting conditions after 0·1 min. Chromatography was altered to remove the potential of highly retained components co-eluting with doxorubicin and IS peaks.

Evaluation of Encapsulated Plasma Doxorubicin by Fluorometry

An independent validated assay was used to evaluate the quenched equivalent and total dequenched doxorubicin concentrations. Unheated and heated aliquots of the same plasma samples, obtained from venous draw, were assayed on the same day using a fluorimeter (POLARstar OPTIMA plate reader). Aliquots of each sample were prepared by diluting plasma 1:1 in mixed pool human plasma (MPHP, SeraLabs, K2 EDTA Anticoagulant, Ref: PK2-123-H) to a volume of 600 µL. Release was achieved by immersing sealed sample tubes in a water bath at 42 ˚C for 15 minutes, whilst the unheated samples were kept at room temperature, protecting from light in both cases. Fluorometry of 200 µL duplicate aliquots of each combination were performed at excitation of 485 nm and emission of 590 nm against a standard curve, which was prepared in mixed pool human plasma from stored stocks of doxorubicin. Unlike the HPLC method, this assay does not attempt to recover the component of circulating doxorubicin bound to plasma proteins pre- or post-heating, and protein-bound doxorubicin demonstrates quenched

fluorescence.[6] However, this effect is somewhat mitigated against by preparing the standards and quality controls in MPHP.

Microscopic Analysis

Microscopic analysis of formalin-fixed paraffin-embedded samples was performed to evaluate (i) tumour morphology using Haemotoxylin and Eosin staining (H&E) histochemistry, (ii) cell viability and vessel distribution using cytokeratin-8 (CK8) and cluster of differentiation 31 (CD-31) immunofluorescence respectively, and, (iii) doxorubicin distribution using confocal microscopy with spectral unmixing to distinguish the fluorescence signature of doxorubicin from auto-fluorescence.7

For CK8 immunofluorescence staining, anti-cytokeratin (CAM 5.2, BD Scientific, Ref: 345779) was used as the primary antibody and goat anti-mouse IgG2a, Alexa Fluor® 488 conjugate (ThermoFisher Scientific, Ref: A-21131) was used as the secondary. For CD-31, monoclonal mouse anti-human CD31 (endothelial) clone JC70A IgG1-Ab (isotype 1) (DAKO, Ref: M0823) was used as the primary antibody and donkey anti-mouse IgG, Alexa Fluor® 488 (Thermo Fisher, Ref: A-21202) was used as the secondary antibody.

For doxorubicin delivery studies, doxorubicin signal was profiled on a Zeiss 710 confocal microscope using formalin-fixed paraffin embedded sections of freshly harvested ex vivo mouse liver exposed to doxorubicin and counterstained with DAPI, and the resulting spectra correlated well with published data for nuclear-bound doxorubicin. Due to wide variation in background tumour auto-fluorescence between different tumour types encountered on the study, optimised confocal laser intensity parameters were used on a tumour-by-tumour basis to avoid saturation from autofluorescence and provide clean spectral unmixing of the doxorubicin signal. Part I samples had matched pre-LTLD and post-LTLD tissue controls, obtained from the same tumour on the same day. These samples were analysed such that samples for any one patient were scanned using the same laser parameters during the same confocal microscopy session.

Calculation of Total Lesion Glycolysis

Total lesion glycolysis 8 (TLG) of the target tumour was calculated at baseline and at two weeks where the tumour burden in the liver was low enough to enable the targeted tumour volume to be defined distinctly from other adjacent tumours on PET-CT. The baseline 3D target tumour volume was automatically generated by the software using the specified SUV_{max} percentage (manually bounded in three planes by hand). Following scan registration in the region of the target liver tumour, the 3D tumour volume was co-registered to the two-week follow-up scan such that the metabolic target volume (MTV) remained the same, to enable TLG to be calculated pre- and post-intervention. Analysis was performed using Hermes Hybrid Viewer V2.5, Hermes Medical Solutions AB, Stockholm, Sweden.

Additional Clinical Results: Laboratory

HPLC Estimations for Biopsy Samples

Whilst the HPLC tumour results were generated by a validated method, estimations were required in three cases as detailed below. In each case, the estimations performed were worst case thus not compromising the primary endpoint.

a. Two post-LTLD tumour samples registered significantly below the response for the lower limit of quantification (LLOQ) of the plasma calibration curve by HPLC (I.02 and I.04, Table 2). Thus, the response for the LLOQ on the day of the analysis of the biopsy was used to estimate what the concentration of doxorubicin would be for that mass of tissue if the response was at the LLOQ. As the response for the sample was significantly lower than that seen for the LLOQ this estimate is an upper-bound, having the impact of potentially under-estimating the drug delivery fold-increase.

b. In I.02 the internal standard (IS) was inadvertently omitted in the post-FUS+LTLS sample, and thus this HPLC value was therefore calculated from the standard curve directly using the doxorubicin peak area only, not doxorubicin peak area as a function of IS area. This is understood to be a lower-bound (Table 2). Unintended omission of the internal standard had the unexpected advantage of demonstrating the absence of any interfering analyte eluting at the same time as the internal standard in a clinical sample.

HPLC Chromatograms for Biopsy Samples

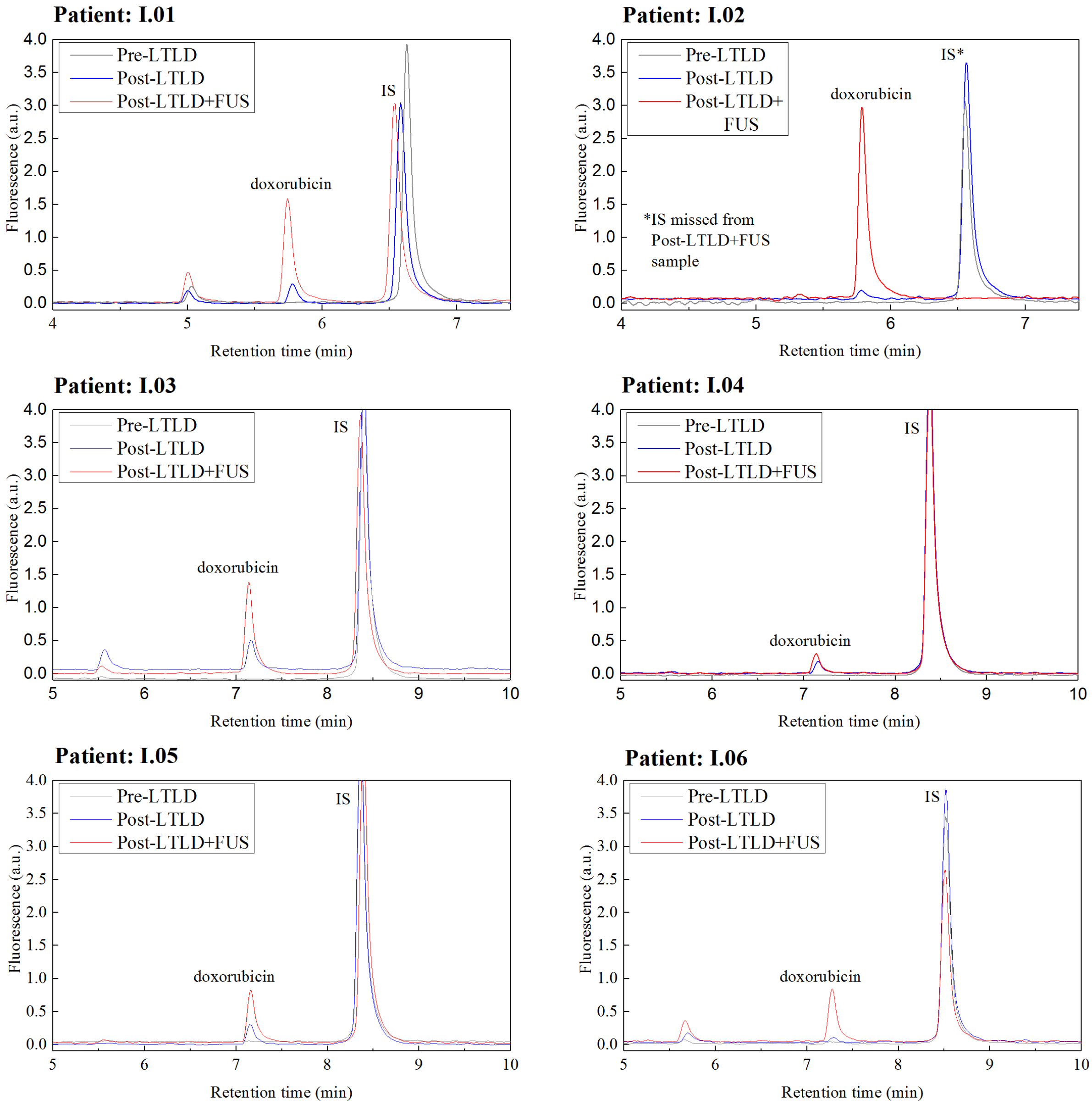

Supplementary Figure 2 (a): Chromatograms obtained from analysis of Part I biopsy samples before LTLD (Pre-LTLD), after LTLD infusion for 30 minutes (Post-LTLD) and following focused ultrasound (FUS)-mediated hyperthermia (Post-LTLD+FUS), using HPLC with fluorescence detection. Daunorubicin was added to each sample as an internal standard (IS) and the ratio of the area under the curve for doxorubicin : daunorubicin was used to calculate the doxorubicin concentrations per gram of tissue. Note that I.01 and I.02 were analysed with different chromatography to the remaining samples (see Detailed Methods) hence elution times are earlier.

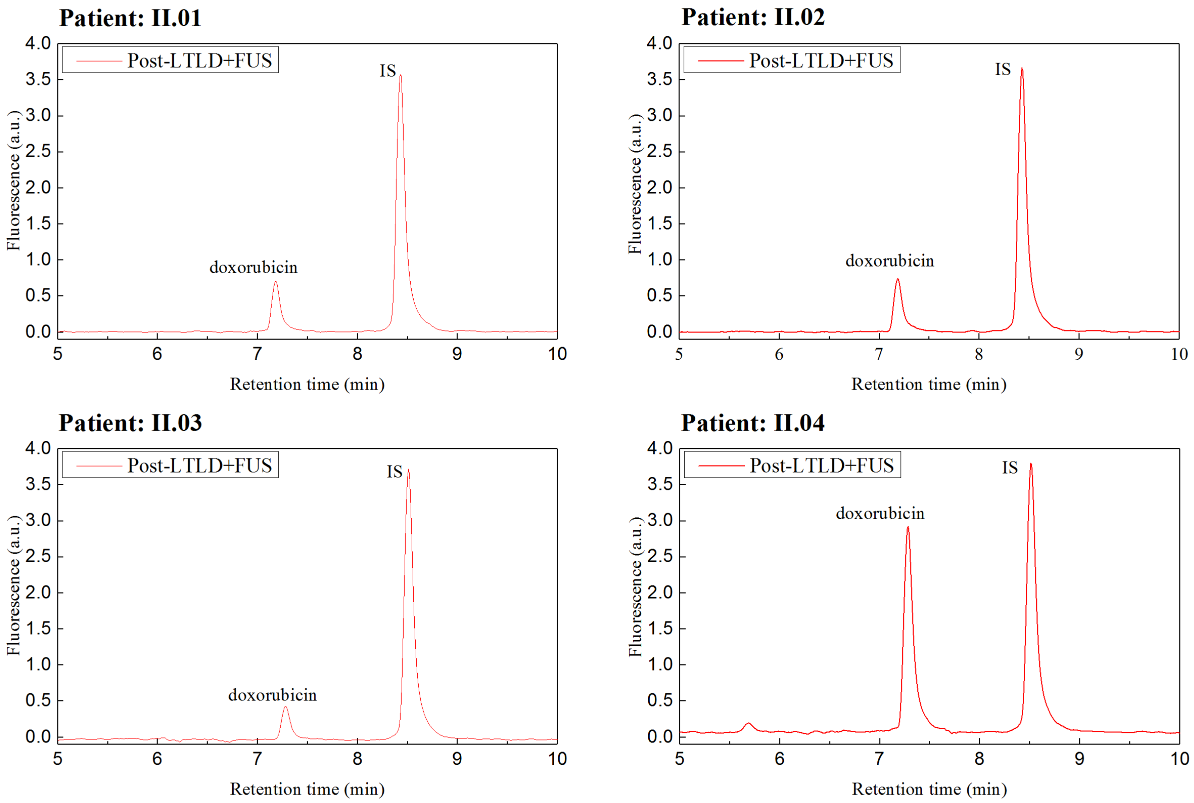

Supplementary Figure 2 (b): Chromatograms obtained from analysis of biopsy Part II samples obtained in the same manner as for Part I samples.

Part I Real-Time Thermometry

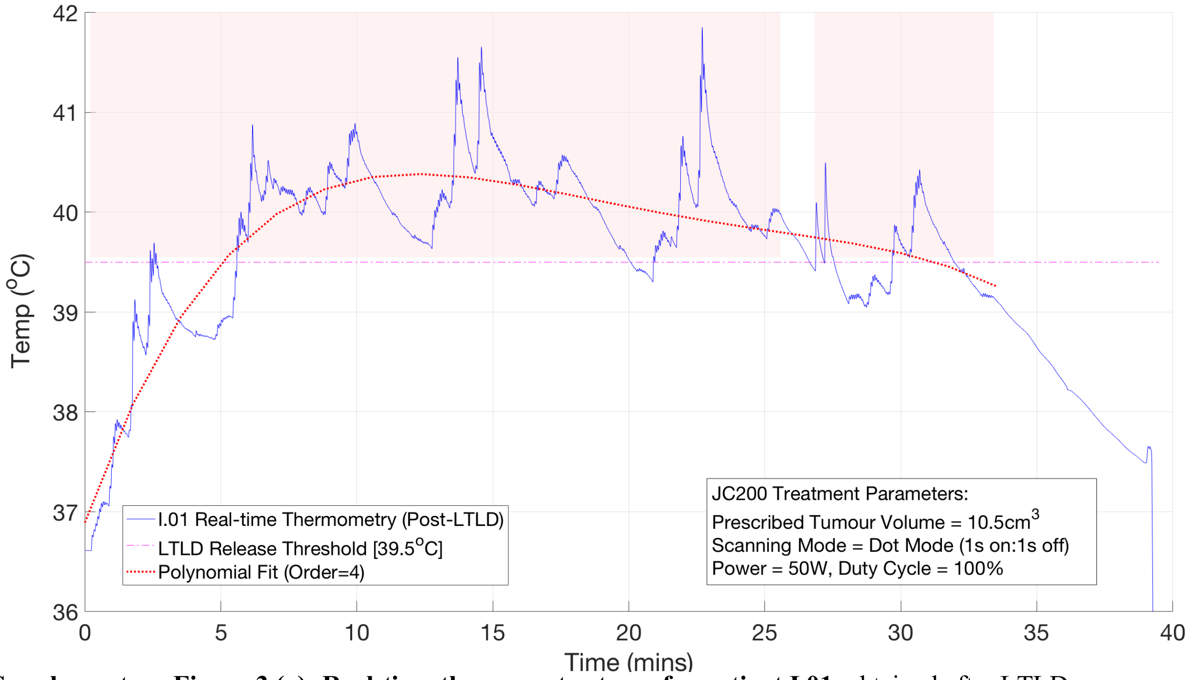

Supplementary Figure 3 (a): Real-time thermometry trace for patient I.01, obtained after LTLD administration during focused-ultrasound exposure (see full figure legend below).

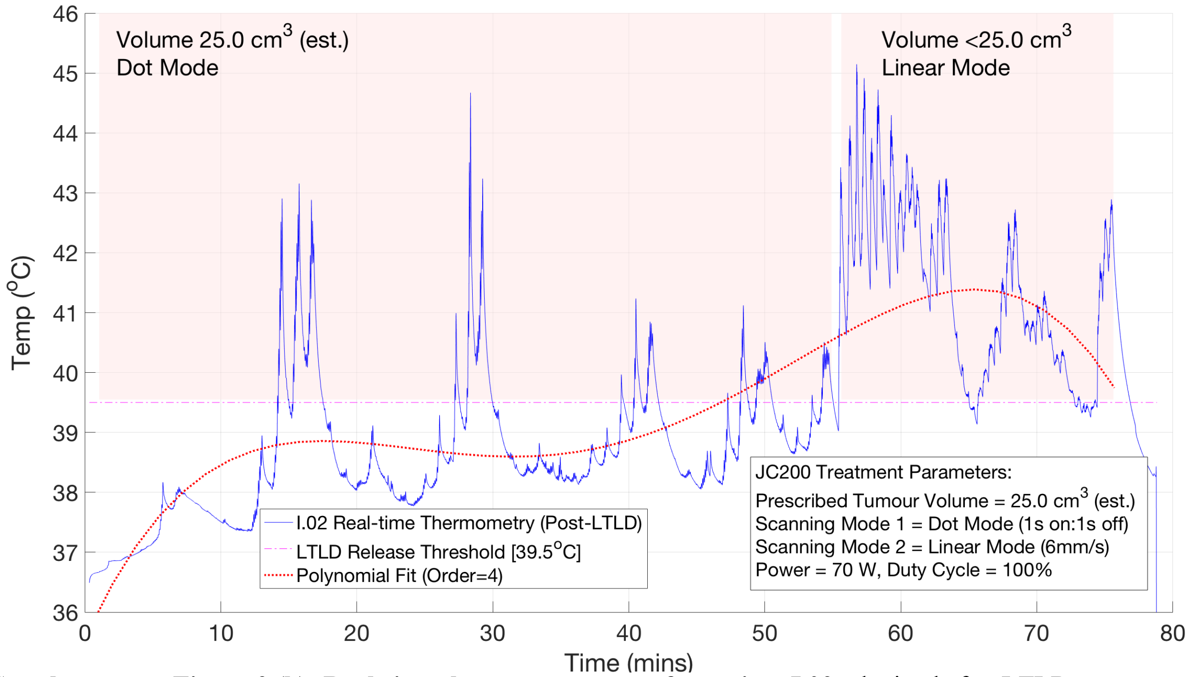

Supplementary Figure 3 (b): Real-time thermometry trace for patient I.02, obtained after LTLD administration during focused-ultrasound exposure (see full figure legend below).

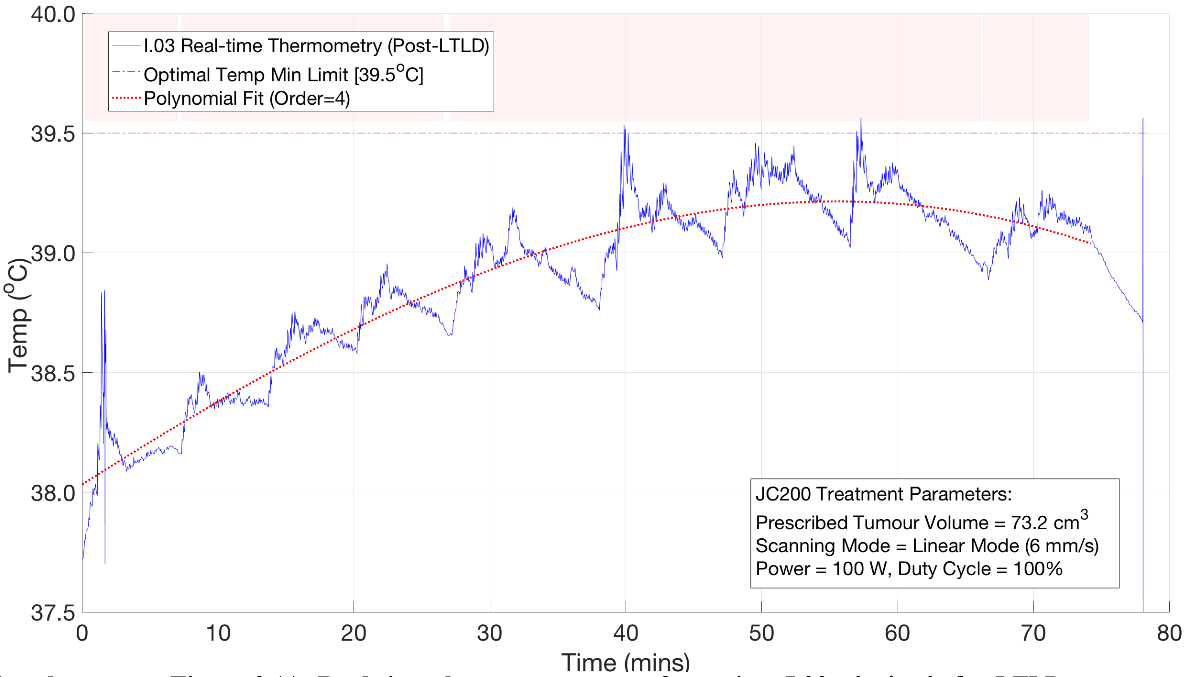

Supplementary Figure 3 (c): Real-time thermometry trace for patient I.03, obtained after LTLD administration during focused-ultrasound exposure (see full figure legend below).

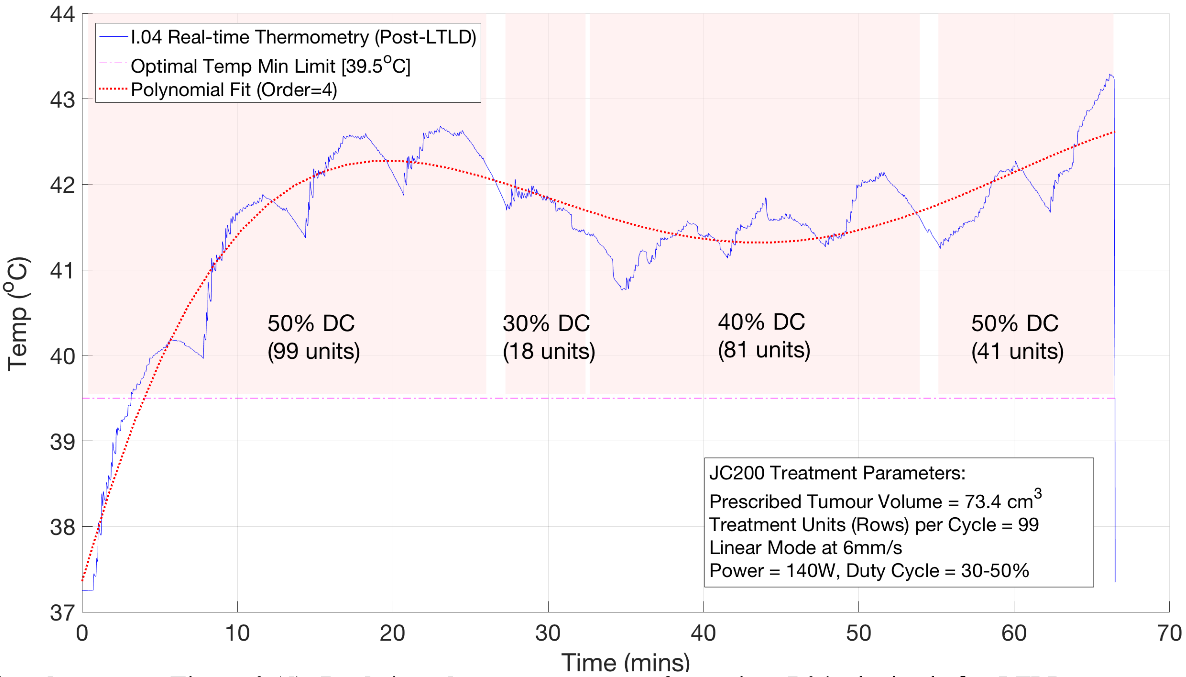

Supplementary Figure 3 (d): Real-time thermometry trace for patient I.04, obtained after LTLD administration during focused-ultrasound exposure (see full figure legend below).

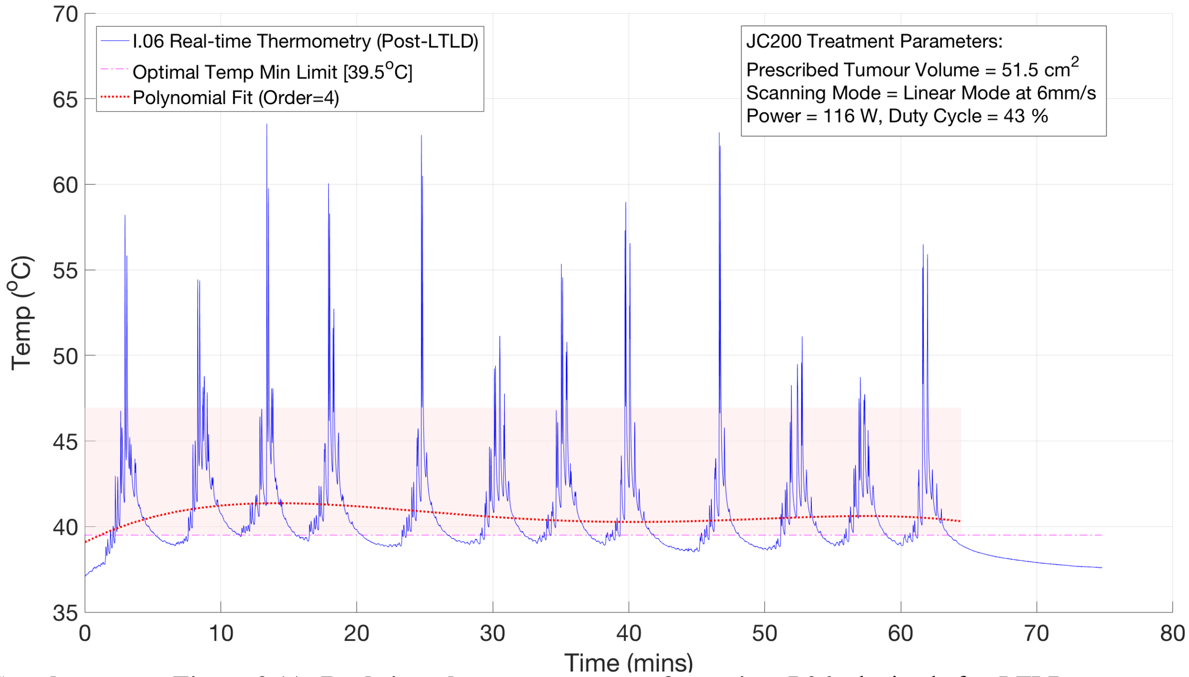

Supplementary Figure 3 (e): Real-time thermometry trace for patient I.06, obtained after LTLD administration during focused-ultrasound exposure (see full figure legend below).

Supplementary Figure 3 (a-e): Illustrative controlled hyperthermia by focused ultrasound for Patients I.01, I.02, I.03, I.04 and I.06. Real-time thermometry data acquired post-LTLD infusion at a 10-millisecond resolution using a custom LabView data-acquisition setup and calibrated thermometry devices inserted through an 18-guage co-axial needle into the target tumour. Calibrated thermometry devices were inserted into the target tumour through a 20 cm 18-gauge coaxial needle. I.01-I.04 used AngioDynamics thermistors whilst cases II.05 (Figure 2, main paper) and II.06 used Medtronic thermocouples. Pink shaded regions represent time when FUS was being applied. In patient I.04 for example, a 73·4 cm^3 prescribed target tumour volume was exposed to 2⅖ FUS exposure cycles at 140 W (7·9 MPa peak rarefactional in situ pressure) in linear mode at 6 mm/s. In this case modification of the duty cycle (DC) was used to maintain temperature at fixed power; the first cycle was at 50% DC, the second at 30% then 40% DC and the final ⅖ cycle at 50% DC. For I.06, a 51·5 cm^3 prescribed target tumour volume was exposed to three complete FUS cycles at 116 Watts (8·2 MPa peak rarefactional in situ pressure) at 43% DC. The temperature spikes above the 45-47 ºC threshold are understood to be artefactual due to direct heating of the thermocouple in the ultrasound field, and the polynomial fit is likely more representative of the mean bulk tumour volume in the proximity of the thermistor tip.

CEM43 Thermal Dose Calculation

For each Part I intervention, cumulative equivalent minutes at 43 ºC (CEM43) thermal dose was calculated from real-time thermometry traces captured during FUS exposure, using a custom MatLab script implementing the Saparato and Dewey method.9 CEM43 thermal dose was below 15 minutes, well under the ablative threshold which is widely accepted as 240 minutes.9 The raw thermometry data for case I.06 demonstrated CEM43 which exceeded 240 minutes. To minimise artefactual effects of having the thermometry device (18G metal thermocouple or thermistor) in the FUS field,10 a polynomial fit of the real-time thermometry data (using least squares fit with order of 4 in MatLab) was applied and used to estimate of the average bulk temperature in the region of the thermocouple tip. Analysis of the fitted thermometry data, thereby reducing artefact, demonstrated a CEM43 of under 15 minutes for all Part I cases (Table 3).

HPLC Analysis of Plasma Samples

Patient ID	Dose given (mg)	Infusion Duration (mins)	Minutes after infusion end when sampled		Plasma Concentration of Total Doxorubicin (µg/g)		
			Post-Drug	Post-FUS	Pre-Drug	Post-Drug	Post-FUS
I.01	91	32	3	57	0·0	N/E*	N/E*
I.02	81	30	1	81	0·0	26·2	23·5
I.03	101	31	5	88	0·0	19·9	6·0
I.04	70	35	11	92	0·0	26·2	5·4
I.05	104	30	1	90	0·0	23·7	8·0
I.06	94	32	4	82	0·0	26·8	11·8
II.01	124	28	2	96	0·0	31·9	19·3
II.02	82	36	0	114	0·0	29·8	16·0
II.03	90	30	0	83	0·0	30·2	12·9
II.04	96	30	1	90	0·0	21·7	12·9

Supplementary Table 5: Doxorubicin plasma pharmacokinetics by HPLC. *For I.01, the first attempt at HPLC analysis for plasma samples, the plasma concentrations for P2 and P3 were off the standard curve and thus they were non-evaluable (N/E). For subsequent runs, the operating procedure was modified to dilute plasma samples to a range back on the curve.

Evaluation of Encapsulated Plasma Doxorubicin by Fluorometry

Whilst the HPLC assay is preferred for accurate quantification of total circulating drug due to its ability to differentiate the specific fluorescence signature of doxorubicin from those of its metabolites, samples were analysed independently by direct fluorescence in both quenched and dequenched form, within 6 hours of sampling. This additional analysis was performed to estimate the ratio of the proportion of circulating doxorubicin that remained liposomal at the time of each venous draw.

Data for I.01-I.04 has been excluded as doxorubicin stocks, which were prepared in MPHP, were found to have degraded when stored at -20˚C, as assessed by HPLC. Stocks were remade and stored at -80 ˚C for patients I.05-06, II.01-04, and were subsequently found to be stable for at least one year.

The pharmacokinetics for total doxorubicin were again comparable to the published data (Supplementary Figure 4, Supplementary Table 5), and within standard error limits of the HPLC plasma results. The post-drug samples demonstrated a mean 5·3-fold (± 1·1 SD) increase in total concentration post-heating, whilst post-delivery samples demonstrated a mean 7·9-fold (± 2·8 SD) increase (n=6). Although not significant, this demonstrates that at the end of the intervention, whilst total circulating doxorubicin was less, a greater proportion of the dose in the blood stream was encapsulated, presumably due to diffusion of any leaked free doxorubicin out of circulation.

Direct Fluorometric Analysis of Unheated and Heated Plasma Samples

Patient ID	Plasma concentration of doxorubicin by direct fluorometry (unheated) (µg/g)			Plasma concentration of doxorubicin by direct fluorometry (heated) (µg/g)			Heated : unheated doxorubicin concentration ratio	
	Pre-Drug	Post-Drug	Post-FUS	Pre-Drug	Post-Drug	Post-FUS	Post-Drug	Post-FUS
I.05	0·0	6·2	2·6	0·0	23·6	10·1	3·8	3·8
I.06	0·0	3·7	1·0	0·0	25·2	11·0	6·8	10·5
II.01	0·0	6·1	2·5	0·0	27·8	17·7	4·6	7·1
II.02	0·0	4·6	1·3	0·0	24·9	13·1	5·4	10·3
II.03	0·0	4·3	1·2	0·0	27·3	10·9	6·3	9·3
II.04	0·0	4·2	1·8	0·0	20·9	11·5	5·0	6·3

Supplementary Table 6: Doxorubicin plasma pharmacokinetics by direct fluorometric analysis. Whilst HPLC results should be taken for accuracy, this assay serves to indicate the proportion of LTLD, which remains encapsulated. In all cases of the total circulating doxorubicin concentration, the proportion of encapsulated drug in the circulation has increased, relative to free doxorubicin, following FUS exposure compared to that immediately post infusion. The ratio of heated:unheated doxorubicin increases following the intervention, which indicates a proportionally smaller fraction of free doxorubicin in the circulation following FUS.

Plasma Pharmacokinetics by Dequenching Assay

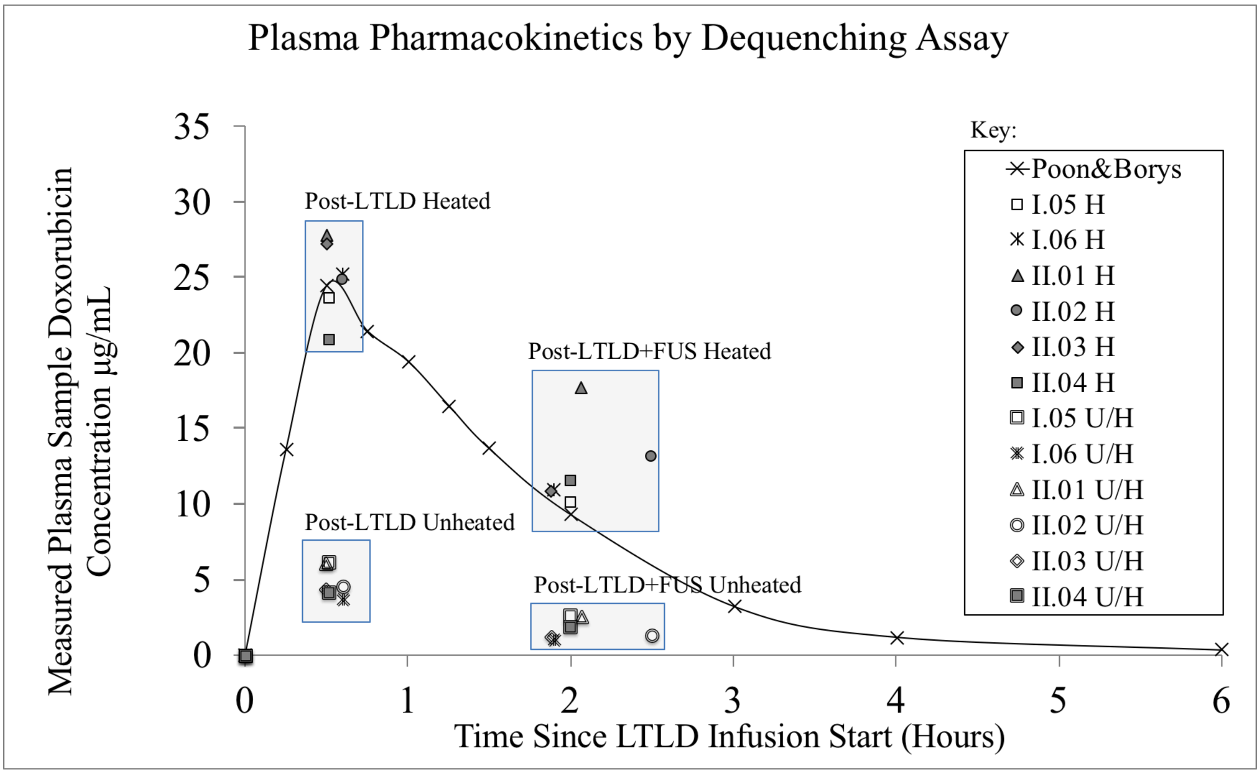

Supplementary Figure 4: Comparison of the LTLD plasma pharmacokinetic data from this study, this time measured by an independent fluorometric dequenching assay with data extracted from that published in Poon & Borys, with express permission.[11] Aliquots of each plasma sample were unheated (U/H) and heated (H) to full release. Data for I.01-I.04 has been omitted as doxorubicin stocks, which were prepared in mixed pool human plasma, were found to have degraded when stored at -20 ˚C, as assessed by HPLC. Stocks were remade and stored at -80 ˚C for I.05 onwards and were subsequently found to be stable. Tabulated data is available in Supplementary Table 6.

Microscopic Drug Delivery Analysis for I.04

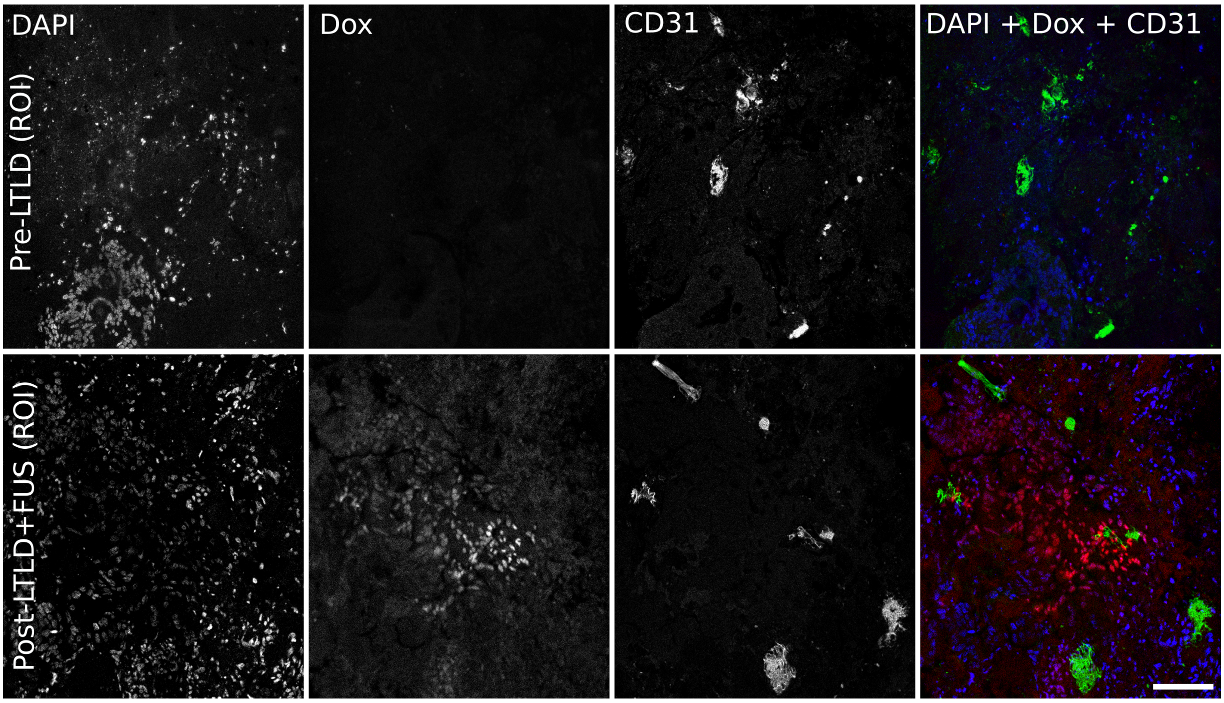

Supplementary Figure 5 (a): Illustrative drug delivery studies by fluorescence microscopy correlated with DAPI and CD31 for I.04 pre-LTLD and post-LTLD+FUS biopsy samples. Fluorescence microscopy for I.04 pre-drug and post-delivery biopsy obtained on the Zeiss 710 confocal microscope with spectral un-mixing. 100 µm Scale bar shown. Nuclei were counter-stained with DAPI (blue). No doxorubicin signal was seen prior to drug infusion (control). Following drug infusion and FUS exposure, doxorubicin (red) was seen to concentrate within viable tumour regions of interest (ROI) with nearby vasculature (green) but was not demonstrated in necrotic and avascular tumour regions.

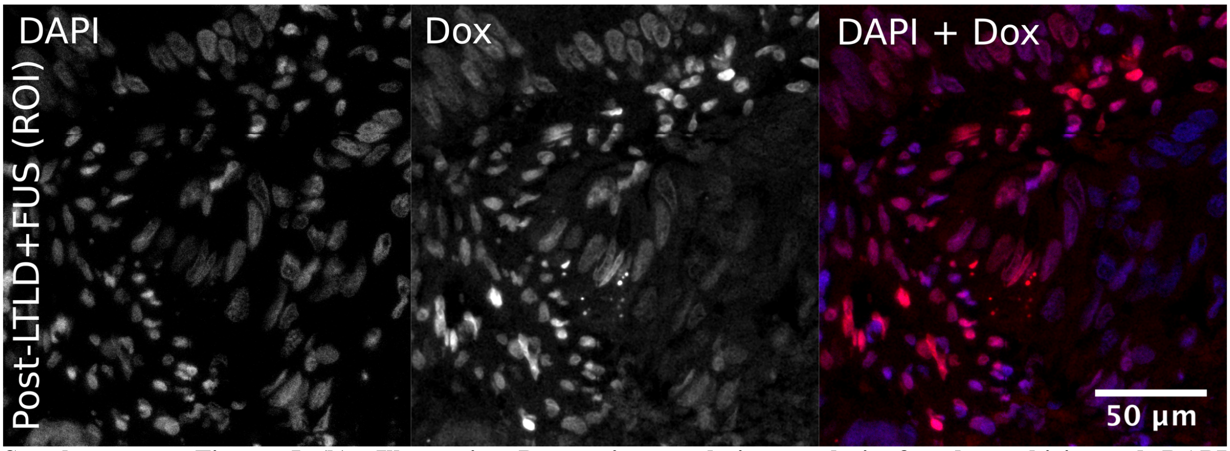

Supplementary Figure 5 (b): Illustrative Pearson's correlation analysis for doxorubicin and DAPI fluorescence microscopy. Analysis for a different ROI in the same slice of the same sample (I.04, post-LTLD+FUS) gave a rank of 0·75, demonstrating co-localization of DAPI and doxorubicin, evidencing doxorubicin's bioavailability and hence suggesting release from the liposomal carrier post-FUS.

__**Microscopic Drug Delivery Analysis for I.05**__

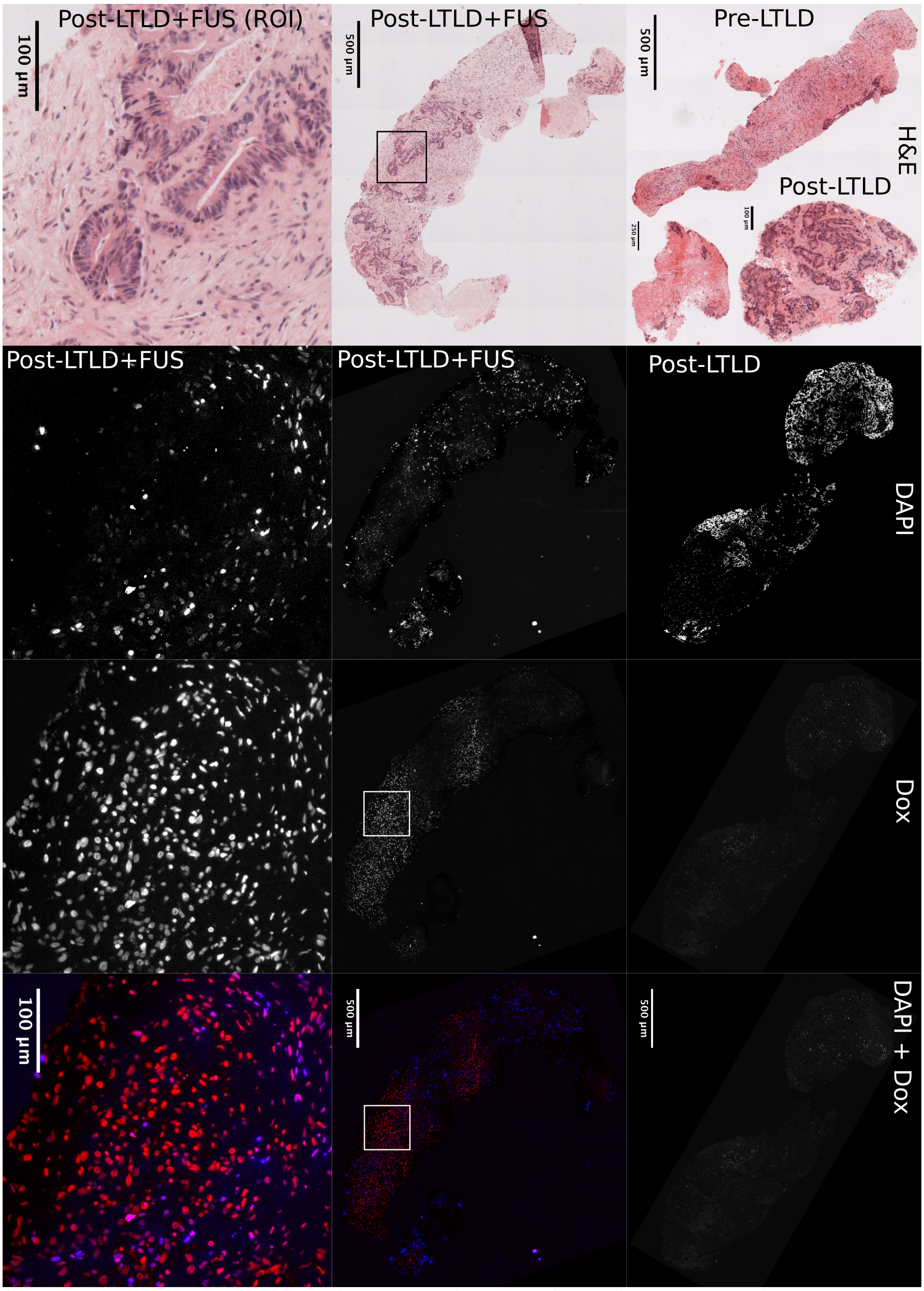

Supplementary Figure 6: Illustrative drug delivery studies by fluorescence microscopy correlated with DAPI and H&E for I.05 pre-LTLD and post-LTLD+FUS biopsy samples. H&E and fluorescence microscopy for I.05 biopsy samples, the latter obtained on the Zeiss 710 confocal microscope with spectral un-mixing. Prior to LTLD infusion, the H&E sample demonstrates mostly fibrous stroma with some tumour islands along the right edge. In the control sample after drug infusion but prior to delivery, H&E samples demonstrate both viable tumour (adenocarcinoma) and a necrotic fragment, whilst fluorescence demonstrates nuclei (DAPI) with little doxorubicin signal (Dox). Post-LTLD+FUS, H&E reveals viable tumour set within fibrous stroma, and a small amount of necrosis (~10%). Fluorescence microscopy correlates well with H&E and demonstrates widespread doxorubicin signal (Dox). Region of interest reveals doxorubicin (red) co-localizes with the DAPI nuclear stain (blue), and when the signal is strongest, doxorubicin appears to out-compete DAPI for nuclear binding sites (composite figure, far right). See Supplementary Figure 6 for cell viability studies of the same samples.

Microscopic Drug Delivery and Cell Viability Analysis for I.06

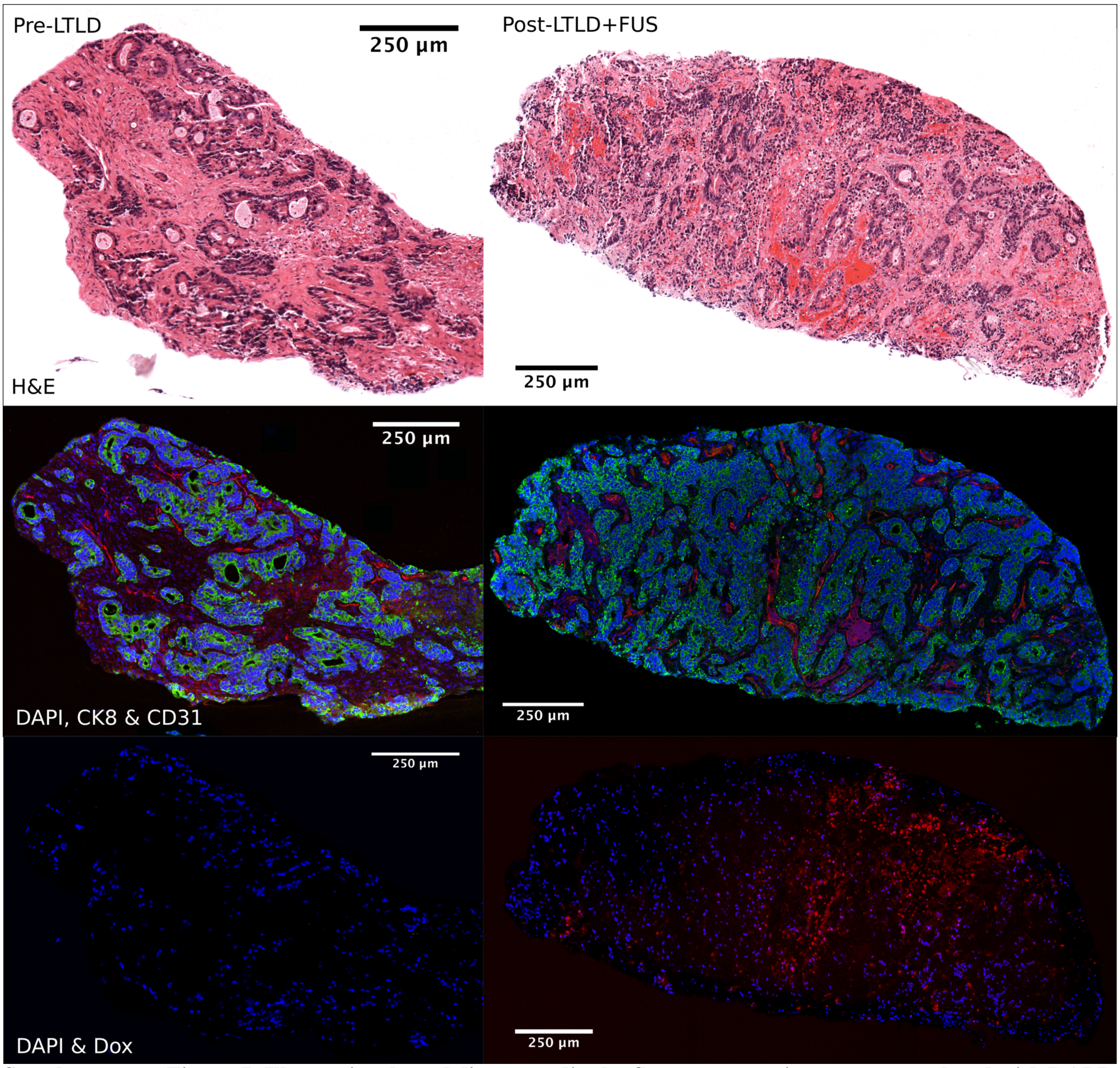

Supplementary Figure 7: Illustrative drug delivery studies by fluorescence microscopy correlated with DAPI, CK8, CD31 and H&E for I.06 pre-LTLD and post-LTLD+FUS biopsy samples.

Top Row: H&E for (regions of) biopsy samples of the target tumour (colorectal adenocarcinoma) for patient I.06 obtained before LTLD was given (left) and after LTLD and FUS exposure (right), demonstrating viable glandular tumour with stroma and focal areas of necrosis. H&E stain for post-LTLD+FUS sample demonstrates approximately 30% viable tumour, and 70% necrotic tumour, and there is a trend for increasing necrosis from right to left.
Middle Row: cell viability studies for an adjacent slice of the same tissue samples by immunofluorescence, obtained on a Zeiss 710 confocal microscope the same day with same settings. Nuclei are stained with DAPI (blue), cells are stained with cytokeratin-8 (CK8, green), a vitality marker and vessels are stained with CD31 (red). Specific CK8 staining of the viable tumour cells is seen both before and after FUS exposure suggesting no immediate thermal ablation, whilst necrotic areas stain poorly (granular staining), correlating well with H&E. Small-medium sized vessels are demonstrated by CD31 (red). Immunofluorescence images obtained using the Zeiss 710 confocal microscope on the same day with the same settings.
Bottom Row: doxorubicin distribution studies for an adjacent slice of the same tissue samples, obtained on a Zeiss 710 confocal microscope using spectral un-mixing the same day with same settings. Blue=DAPI and Red=Doxorubicin. Doxorubicin signal is absent in the sample obtained before it was given. Post LTLD+FUS, nuclear doxorubicin is demonstrated with micro-regional variation and the greatest nuclear doxorubicin signal is seen in the more vascular regions.

Microscopic Drug Delivery and Cell Viability Analysis for II.04

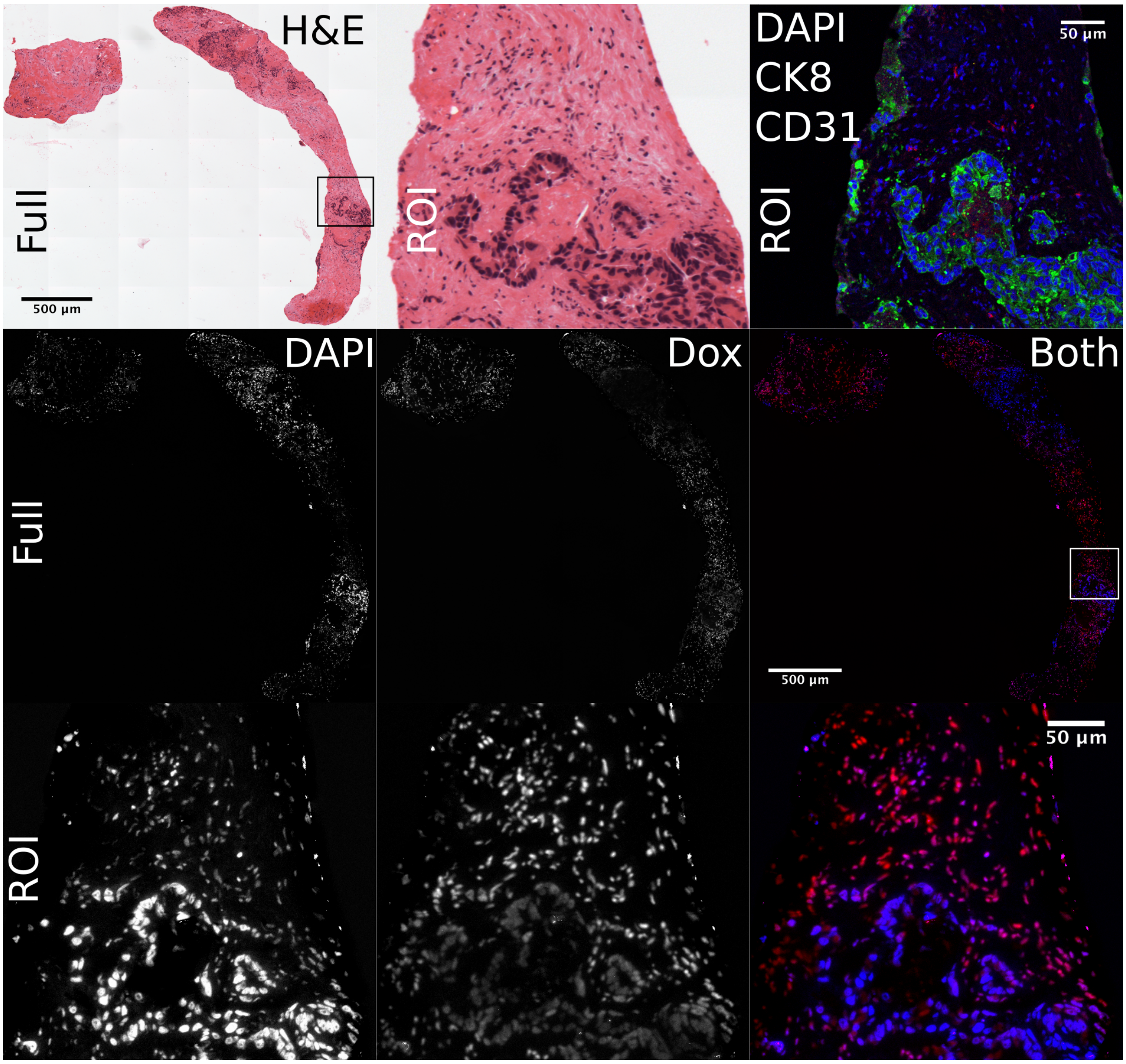

Supplementary Figure 8: Illustrative drug delivery studies by fluorescence microscopy correlated with DAPI, CK8, CD31 and H&E for II.04 post-LTLD+FUS biopsy samples. TOP ROW: H&E stain demonstrates mostly stroma with early necrotic tumour islands and approximately 10% tumour necrosis, but an absence of coagulative necrosis (left) and a region of interest (ROI) containing tumour cells is shown (middle). Immunofluorescence of the same ROI demonstrates tumour cells stain specifically for CK8 (green) and CD31 (red) reveals a few small vessels (right). MIDDLE ROW: Fluorescence microscopy shows DAPI nuclear stain and doxorubicin throughout the tumour sample, seen together on the overlay image (right) (blue=DAPI, red=Doxorubicin). BOTTOM ROW: Corresponding images for the ROI, demonstrating co-localisation of doxorubicin and DAPI in the stroma and tumour nuclei, the latter appearing less condensed. Immunofluorescent images obtained on a Zeiss 710 confocal microscope with spectral un-mixing.

Microscopic Cell Viability Analysis for I.05

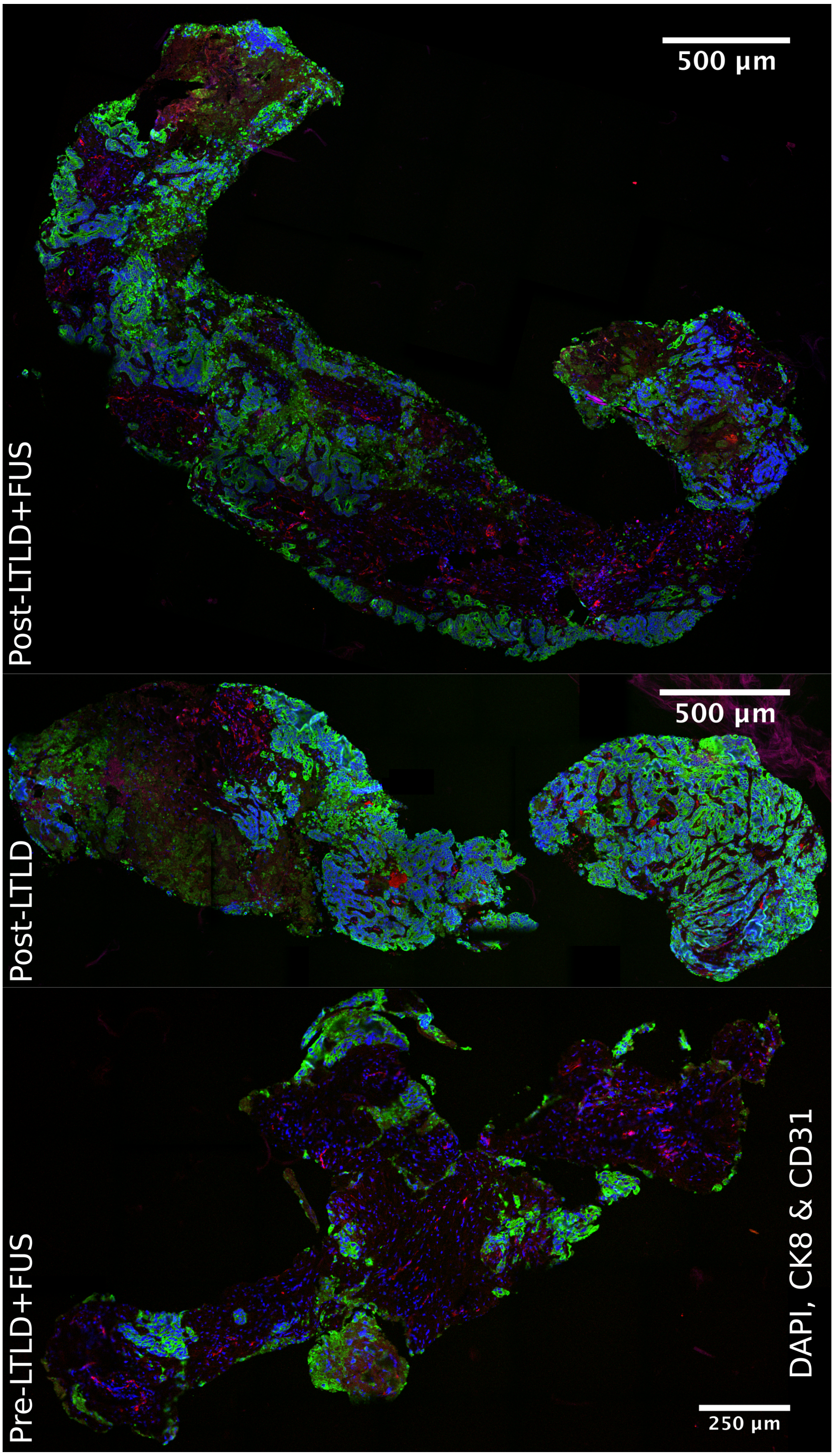

Supplementary Figure 9: Illustrative DAPI, CK8 and CD31 immunofluorescence microscopy for I.05 pre-LTLD, post-LTLD and post-LTLD+FUS biopsy samples. Obtained on a Zeiss 710 confocal microscope, with DAPI stain for nuclei (blue). Cytokeratin-8 (CK8) signal (green) persists following FUS-mediated delivery. Vascular staining (red) is relatively sparse in all samples.

Additional Clinical Results: Radiology

Radiology Figures

Figure Legend for Supplementary Figures 10-12: For each patient, the target tumour was assessed for radiological response across three different imaging modalities, shown in axial section at closest matching slices. In each case, the targeted tumour, each indicated by a red arrow on the baseline scan, was exposed to both systemic LTLD and focused ultrasound in the direction indicated by the red triangle. Control tumours, which received systemic LTLD alone, are indicated by white arrows on PET-CT imaging, when incidentally captured on the same axial slice. Baseline images were obtained one day prior to treatment (leftmost column) and follow-up images, co-localised for each modality, obtained at one day post-intervention (second column from left, MRI only), approximately two weeks (second column from right) and four weeks (right most column) following the study intervention. In patient I.06, due to coalition with an adjacent anterior located metastasis, the anterior tumour border was indistinct on ultrasound, and consequently the posterior portion of the more anterior tumour was also included in the treatment volume for hyperthermia, treated through the same intercostal space. The four-week scans for patient II.03 were not performed due to clinical progression.

A Partial Response (PR) according to CHOI modified to assess a single tumour was seen in the target tumour alone at 2 and 4 weeks in patients I.01, I.04, I.06 and II.04, the latter case also demonstrating PR according to modified PERCIST. In patients I.05 and II.02, a PR according to CHOI modified for a single tumour was seen in both the target tumour and at least one control tumour at 2 and 4 weeks. All other target (4 (40%) of 10) and control (13 (81.3%) of 16) tumours assessed ultimately demonstrated either stable or progressive disease by modified RECIST, CHOI or PERCIST by 2 or 4 weeks

Figure Legend for Supplementary Figures 10 (a+b): Baseline and follow-up ^{18}F-FDG PET-CT axial imaging of the target tumour for all patients who received the intervention. The four-week PET-CT for patient I.02 was non-diagnostic scan due to vascular access issues at time of tracer injection and has been omitted. PERCIST partial metabolic response is demonstrated in the target tumour for patient II.04, but not the control tumours, when criteria are modified to evaluate a single tumour at a time.

Figure Legend for Supplementary Figures 11 (a+b): Baseline and follow-up perfusion CT axial imaging of the target liver tumour for all patients who received the intervention. The four-week perfusion CT for patient I.02 was effectively a non-contrast scan due to vascular access issues at time of contrast injection. CHOI partial response by density criteria is demonstrated in the target tumour for patients I.01, I.04, I.06 and II.04, but not the control tumours, when criteria are modified to evaluate a single tumour at a time.

Figure Legend for Supplementary Figures 12 (a+b): Baseline and follow-up dynamic contrast enhanced MRI axial imaging of the target liver tumour for all patients who received the intervention.
The four-week MRI for patient I.02 was effectively a non-contrast scan due to vascular access issues at time of contrast injection. For patients I.05 and II.01 and II.04, the MRI images demonstrate curved artefacts through the liver due to difficulties in achieving homogenous shimming. Of note, for patients I.04 and I.06, the MRI for the baseline (day prior to treatment) demonstrates no immediate ablative change (i.e. no reduction in contrast) which would be expected in high intensity focused ultrasound ablation (HIFU) where there is direct destruction of the tumour microvasculature. However, reduced perfusion is seen subsequently at two weeks, thought to be due to the natural time course of chemo-ablation. For patient II.04 there is reduced perfusion on day 1, thought to be due to vasoconstriction. More detailed interpretation of MRI perfusion changes is provided in Supplementary Table 7.

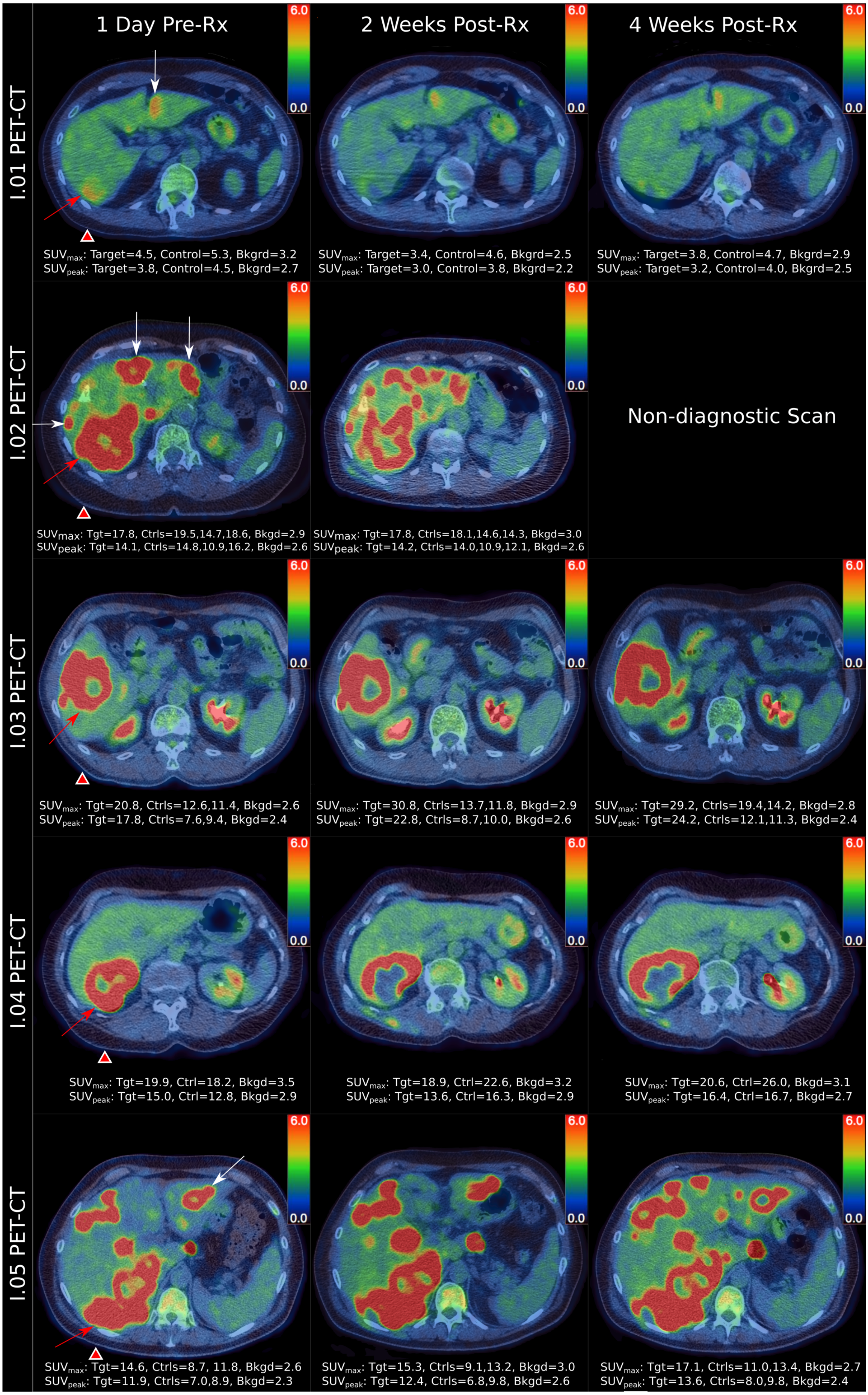

Supplementary Figure 10(a): PET-CT of target tumours for I.01-05 (see detailed figure legend above).

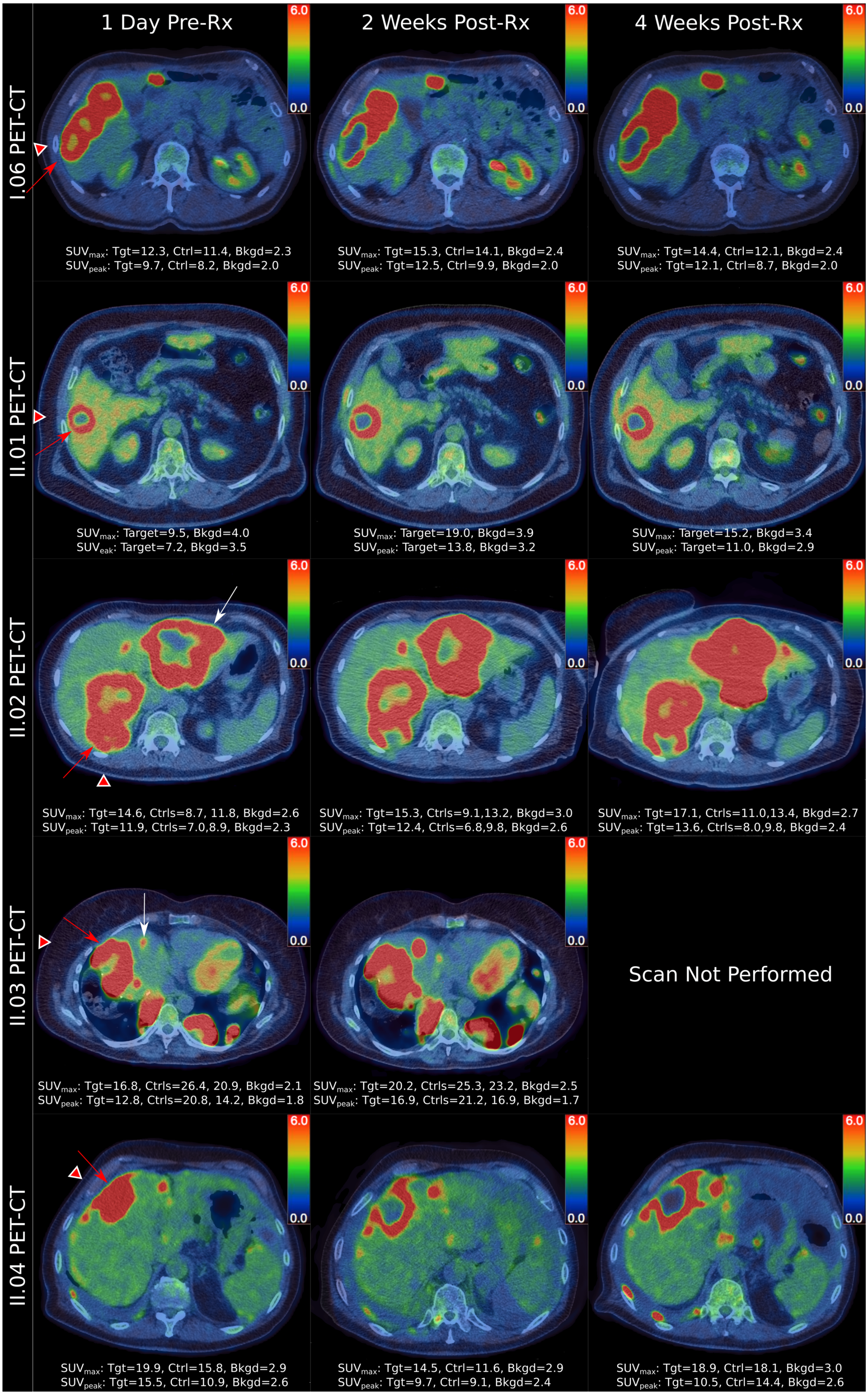

Supplementary Figure 10(b): PET-CT of target tumours for I.06, II.01-04 (see detailed figure legend above).

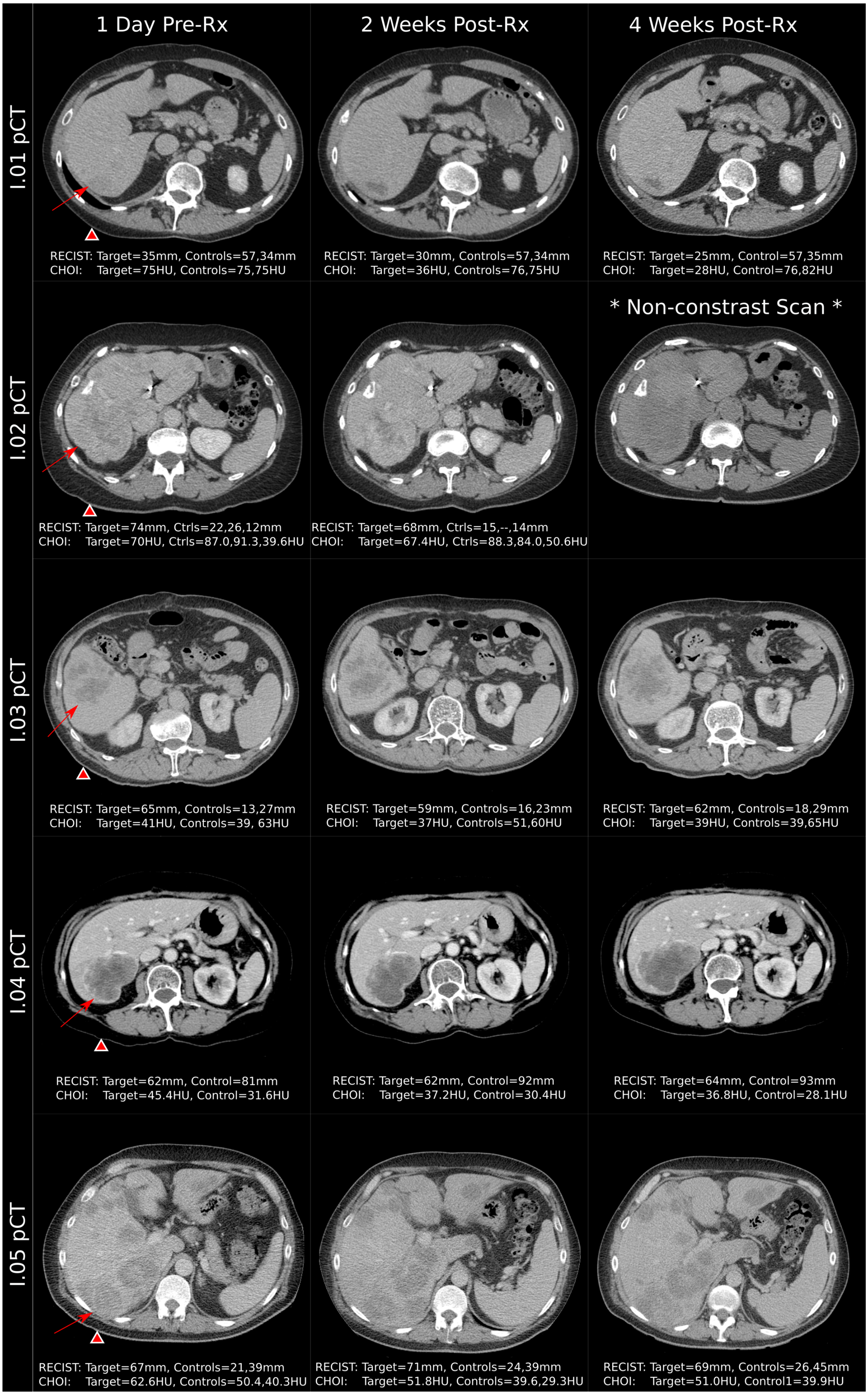

Supplementary Figure 11(a): Perfusion CT of target tumours for I.05, II.01-04 (see full figure legend above).

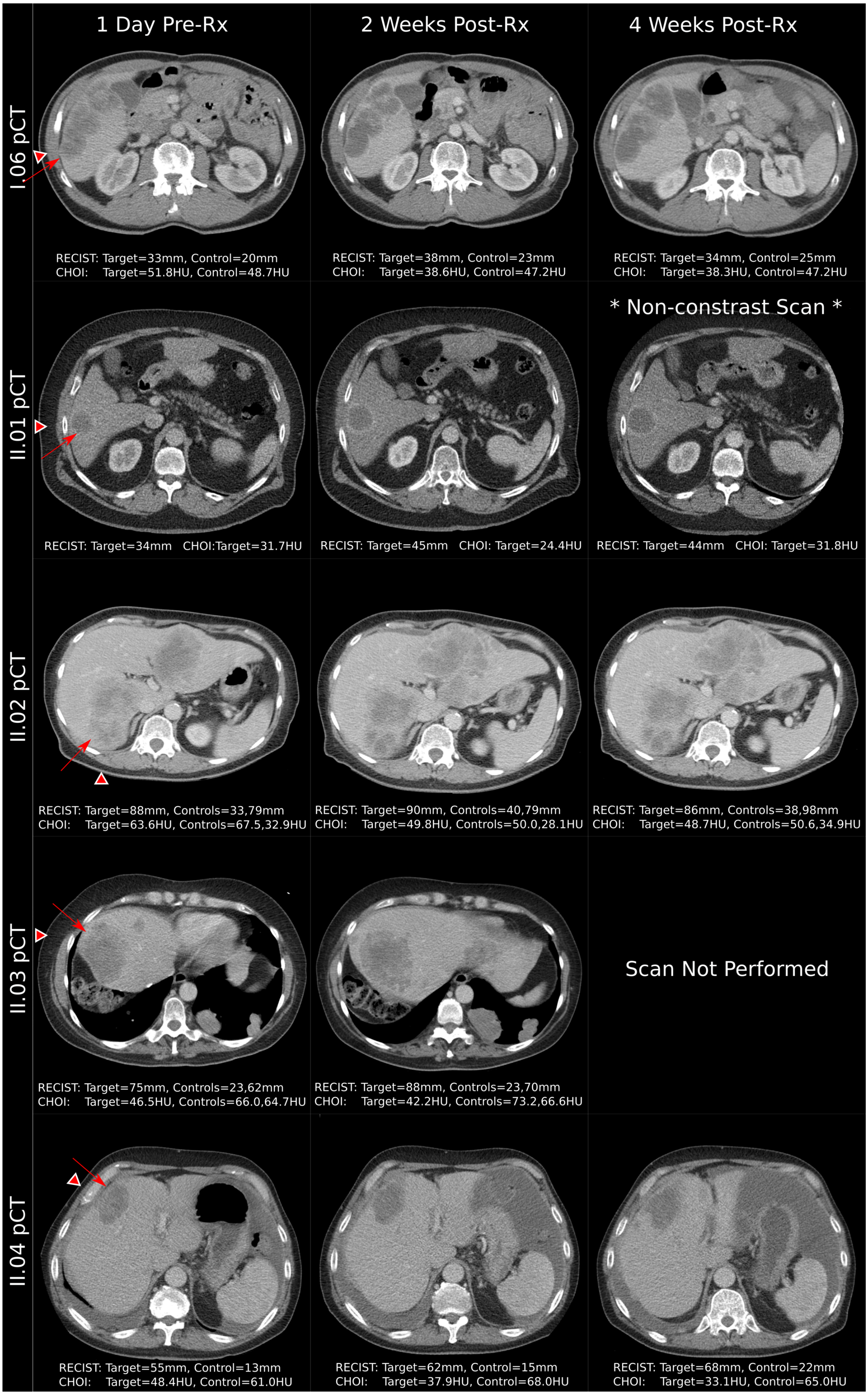

Supplementary Figure 11(b): Perfusion CT of target tumour for I.05, II.01-04 (see full figure legend above).

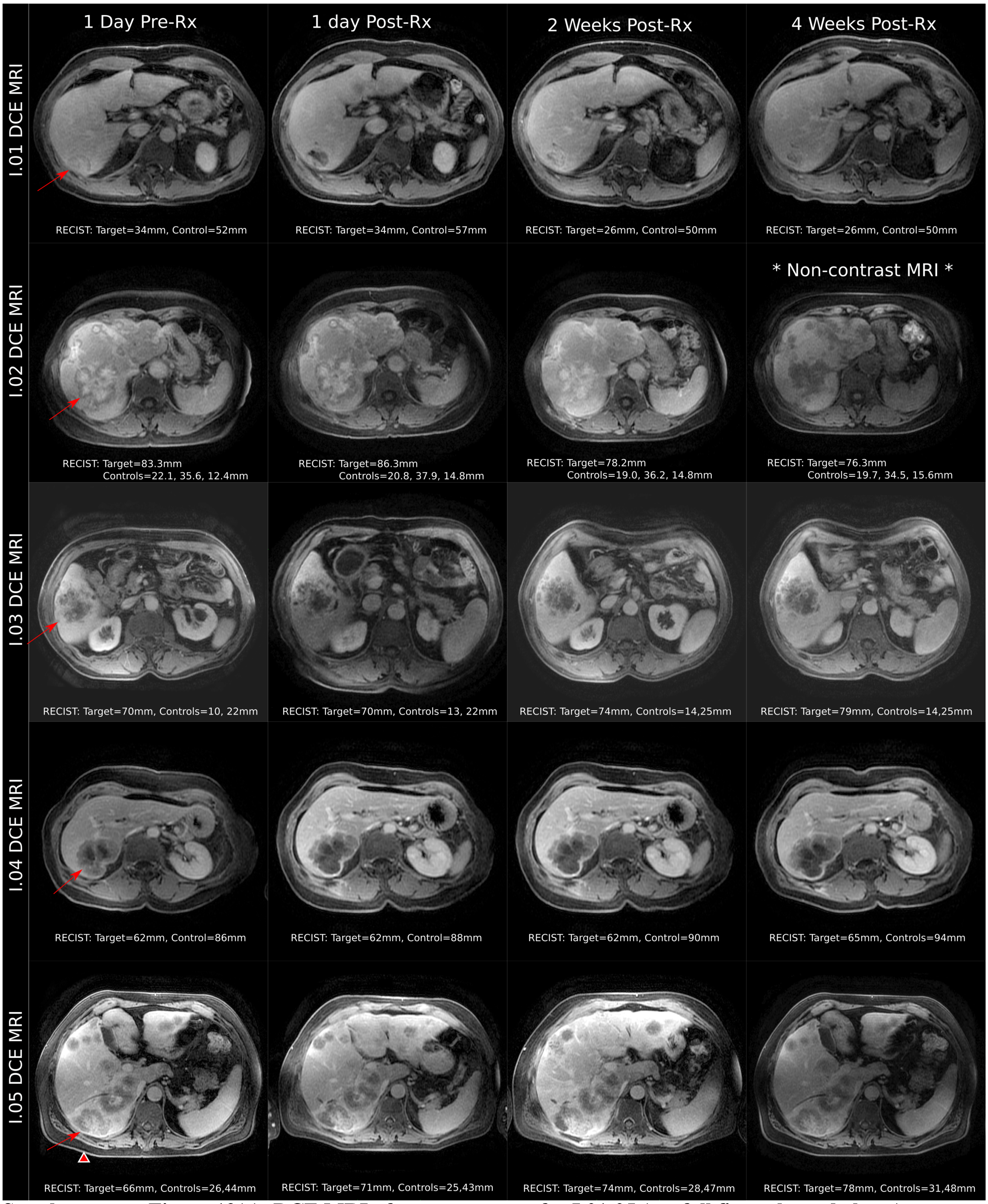

Supplementary Figure 12(a): DCE MRI of target tumours for I.01-05 (see full figure legend above).

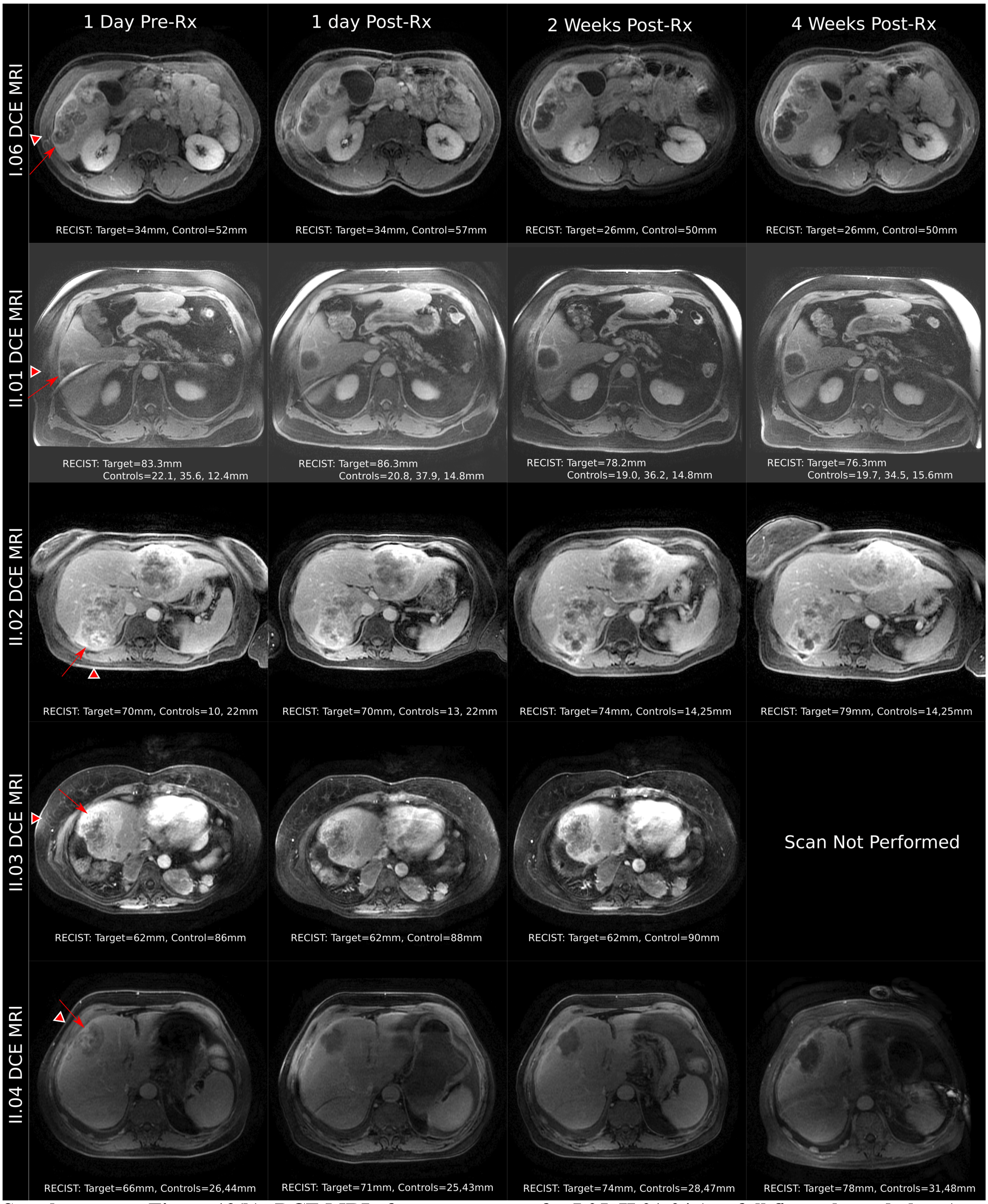

Supplementary Figure 12(b): DCE MRI of target tumours for I.05, II.01-04 (see full figure legend above).

PET-CT Glycolytic Volume Analysis

Patient ID	Percentage of SUV_{max}	MTV of Target Tumour (cm^3)	TLG of Target Tumour		Percentage Change (+/-)	Comment
			Baseline	2 weeks		
**I.01**	30	20·4	69·8	44·4	-36·4	
**I.02**						Excluded (IV access difficulties)
**I.03**						Excluded (high tumour burden)
**I.04**	30	86·8	741·3	573·4	-22·6	
**I.05**						Excluded (high tumour burden)
**I.06 (a)**	30	14·8	1047·6	1023·7	-2·3	Full coalition volume included
**I.06 (b)**	30	67·8	482·3	394·5	-18·2	Target tumour volume only
**II.01**	45	49·1	263·3	368·8	+40·0	
**II.02**	40	166·7	1218·9	1290·0	+5·8	
**II.03**						Excluded (high tumour burden)
**II.04**	35	58·1	629·6	338·4	-46·3	

Supplementary Table 7: Summary of ^{18}F-FDG PET-CT glycolytic volume analysis of target tumour volume at baseline and 2 weeks, following co-registration of serial PET-CTs. Patients with a high liver tumour burden were excluded from analysis (I.03, I.05, II.03). In the case of I.06, at baseline the PET-CT demonstrated a coalition of adjacent tumours (Supplementary Figure 10(b)), and the TLG for both (a), the full coalesced tumour volume, and (b), the targeted volume (using a restricted bounding box through the target rib space) are included.

Day 1 MRI Perfusion Analysis

Patient ID	Mean DCE Signal Intensity Ratio of Target Tumour / Normal Liver (Ratio)		
	Baseline	1 Day Post-intervention	2 Weeks Post-intervention
**I.01**	279.6 / 259.7 (1.07)	170.1 / 266.0 (0.64)	299.8 / 303.0 (0.99)
**I.02**	Excluded from analysis		
**I.03**	Excluded from analysis		
**I.04**	193.5 / 271.6 (0.71)	166.7 / 310.3 (0.54)	186.1 / 455.2 (0.41)
**I.05**	243.0 / 224.6 (1.08)	174.4 / 217.2 (0.80)	188.7 / 316.6 (0.59)
**I.06**	205.7 / 275.5 (0.75)	198.2 / 255.5 (0.78)	130.7 / 245.6 (0.53)
**II.01**	62.7 / 106.9 (0.59)	71.6 / 107.4 (0.67)	57.5 / 118.9 (0.48)
**II.02**	376.7 / 336.0 (1.12)	356.4 / 338.2 (1.05)	206.5 / 342.8 (0.60)
**II.03**	386.7 / 321.1 (1.20)	324.8 / 306.3 (1.06)	391.3 / 349.1 (1.12)
**II.04**	337.1 / 222.4 (1.52)	209.7 / 203.3 (1.03)	198.3 / 230.1 (0.86)

Supplementary Table 8: A region of interest (ROI) mean intensity analysis was explored to assess changes in the focused ultrasound exposed region of the target tumour, using a circle of area 2·5-3·0 cm^3. Due to lack of US/MRI fusion software, each ROI was defined by hand in the peripheral tumour in the FUS path, using correlation with cross-sectional imaging and B-mode images captured at time of intervention. At matched time points of dynamic contrast enhancement (DCE), mean signal intensities in the ultrasound exposed region of the target liver tumour and selected region of normal liver are given (arbitrary units) and ratio of tumour : normal liver is expressed (to two decimal places). Analysis for patients I.02 and I.03 were omitted due to vascular access issues and lack of day 1 MRI respectively.

Correlation of MRI Perfusion with PET-CT

Patient ID	Reduced perfusion on MRI on Day 1?	Reduced perfusion on MRI at 2 weeks?	Localised PET-CT Response?	Radiologist MRI Short Report on Target Tumour Perfusion	Radiologist Interpretation
**I.01**	Y	N	Y	Reduced perfusion across whole tumour on day 1 MRI, which is restored to baseline at the 2-week follow-up	Temporary vasoconstriction of the tumour vasculature or thrombus, which subsequently demonstrates chemo-ablation
**I.02**	N	N/A	N/A	Excluded: IV access issues	N/A
**I.03**	N/A	N	N	Day 1 MRI not performed, no appreciable change in perfusion on 2-week MRI, correlating with PET	Non-responding tumour
**I.04**	N	Y	Y	No appreciable change in perfusion on day 1 MRI, but much reduced perfusion at 2 weeks, correlating with PET	Chemo-ablation
**I.05**	Y	Y	Y	Reduced perfusion in treated region on day 1 MRI, which persists at 2 weeks, correlating with PET	Early tumour response (<24 hours), or temporary vasoconstriction of the tumour, which subsequently demonstrates chemo-ablation
**I.06**	N	Y	Y	No appreciable change in perfusion on day 1 MRI, but there is markedly reduced perfusion at 2 weeks, correlating with PET	Chemo-ablation
**II.01**	Y	Y	N	The well-perfused (viable) tumour rim demonstrates reduction in its thickness with increased non-perfused (necrotic) central zone on day 1 and 2-week MRI, whilst the PET demonstrates no appreciable change	There are early (<24 hours) MRI changes which persist at 2 weeks but no clear PET response consistent with non-responding tumour
**II.02**	N	Y	Y	No appreciable change in perfusion on day 1 MRI, but decreased perfusion in treated area at 2 weeks, correlating with PET	Chemo-ablation
**II.03**	N	N	N	No appreciable change in perfusion on day 1 or 2-week MRI, correlating with PET	Non-responding tumour
**II.04**	Y	Y	Y	The well-perfused (viable) tumour rim demonstrates reduction in its thickness with increased non-perfused (necrotic) central zone on day 1 and 2-week MRI, and further reduced central perfusion at 4 weeks, correlating with PET	Early tumour response (<24 hours), or temporary vasoconstriction of the tumour, which subsequently demonstrates chemo-ablation

Supplementary Table 9: Summary of changes to perfusion seen on dynamic contrast MRI at day 1 and 2 weeks, as related to ^{18}F-FDG PET-CT at 2 weeks, in the target tumour, as reported and interpreted by the same consultant radiologist. Reports for patient I.02 were excluded due to vascular access issues leading to variability of contrast uptake and thus non-comparable follow-up MRI scans at weeks 2 and 4 comparable to baseline and day 1.

References

1 Chung DYF, Tse DML, Boardman P, *et al.* High-frequency jet ventilation under general anesthesia facilitates CT-guided lung tumor thermal ablation compared with normal respiration under conscious analgesic sedation. *J Vasc Interv Radiol* 2014; **25**: 1463–9.

2 Wood BJ, Poon RT, Locklin JK, *et al.* Phase I study of heat-deployed liposomal doxorubicin during radiofrequency ablation for hepatic malignancies. *J Vasc Interv Radiol* 2012; **23**: 248–55.

3 Rothuizen J, Twedt DC. Liver biopsy techniques. Vet Clin North Am Small Anim Pr. 2009; **39**: 469–80.

4 Al-Abd AM, Kim NH, Song SC, Lee SJ, Kuh HJ. A simple HPLC method for doxorubicin in plasma and tissues of nude mice. *Arch Pharm Res* 2009; **32**: 605–11.

5 Cummings J, Stuart JF, Calman KC. Determination of adriamycin, adriamycinol and their 7-deoxyaglycones in human serum by high-performance liquid chromatography. J Chromatogr. 1984; **311**: 125–33.

6 Bi S, Sun Y, Qiao C, Zhang H, Liu C. Binding of several anti-tumor drugs to bovine serum albumin: Fluorescence study. *J Lumin* 2009; **129**: 541–7.

7 Lyon PC. DPhil Thesis, University of Oxford: Targeted Release from Lyso-thermosensitive Liposomal Doxorubicin (ThermoDox®) Using Focused Ultrasound in Patients with Liver Tumours. 2016.

8 Larson SM, Erdi Y, Akhurst T, *et al.* Tumor treatment response based on visual and quantitative changes in global tumor glycolysis using PET-FDG imaging. The visual response score and the change in total lesion glycolysis. *Clin Positron Imaging* 1999; **2**: 159–71.

9 Sapareto SA, Dewey WC. Thermal dose determination in cancer therapy. *Int J Radiat Oncol Biol Phys* 1984; **10**: 787–800.

10 Hynynen K, Martin CJ, Watmough DJ, Mallard JR. Errors in temperature measurement by thermocouple probes during ultrasound induced hyperthermia. Br J Radiol. 1983; **56**: 969–70.

11 Poon RT, Borys N. Lyso-thermosensitive liposomal doxorubicin: a novel approach to enhance efficacy of thermal ablation of liver cancer. *Expert Opin Pharmacothera* 2009; **10**: 333–43.

Full Study Protocol

The remainder of the web appendix is dedicated to the full ethically-approved study protocol.

TARDOX

Full title: A Proof of Concept Study to Investigate the Feasibility of Targeted Release of Doxorubicin from Lyso-thermosensitive Liposomal (LTSL) Doxorubicin (ThermoDox®) Using Focused Ultrasound in Patients with Primary or Secondary Liver Tumours

Short title: Targeted chemotherapy using focused ultrasound for liver tumours

**Protocol Version & date:**	Version 3.0 06Apr2016
**Sponsor Protocol Number:**	OCTO_040
**Ethics Number:**	14/NE/0124
**EudraCT Number:**	2014-000514-61
**Clintrials.gov. No.**	NCT02181075

Sponsored by the University of Oxford

Funders: **NIHR Oxford Biomedical Research Centre**
Joint Research Office, Block 60, Churchill Hospital, Old Road, Headington, Oxford OX3 7LE

Celsion Corporation
Lawrenceville, NJ, USA

Confidentiality Statement

This document contains confidential information that must not be disclosed to anyone other than the Sponsor, the trials office, the Investigator Team, host NHS Trust(s), regulatory authorities, and members of the Research Ethics Committee.

General contact information

**Trial Office (OCTO)**	TARDOX Trial Office<br>Oncology Clinical Trials Office (OCTO)<br>Department of Oncology, The University of Oxford<br>Oxford Cancer and Haematology Centre<br>Churchill Hospital<br>Oxford<br>Tel: +44 (0)1865 227190<br>Fax: +44 (0)1865 227039<br>Email: OCTO-TARDOX@oncology.ox.ac.uk<br>Website: http://www.octo-oxford.org.uk<br>Clinical Trial Coordinator: Miss Lucy Boyle
**Chief Investigator**	Professor Mark Middleton<br>Consultant Medical Oncologist<br>Department of Oncology, University of Oxford<br>Oxford Cancer and Haematology Centre Churchill Hospital Oxford OX3 7LE<br>Tel: +44 (0)1865-235315<br>Fax: +44 (0)1865-235981<br>Email: mark.middleton@oncology.ox.ac.uk
**Lead Senior Scientific Investigator**	Professor Fergus Gleeson<br>Consultant Radiologist<br>Department of Radiology Churchill Hospital Oxford OX3 7LE<br>Tel.: +44 (0) 28 95048492<br>Email: fergus.gleeson@oncology.ox.ac.uk
**Lead Senior Scientific Investigator**	Professor Constantin Coussios<br>Professor of Biomedical Engineering<br>Head of the Biomedical Ultrasonics, Biotherapy and Biopharmaceuticals Laboratory Institute of Biomedical Engineering<br>Old Road Campus Research Building University of Oxford OX3 7DQ<br>Tel: +44 (0) 1865-617726<br>Fax: +44 (0) 1865-617728<br>Email: constantin.coussios@eng.ox.ac.uk
**Sponsor**	Ms Heather House<br>University of Oxford<br>Joint Research Office, Block 60, Churchill Hospital<br>Oxford OX3 7LE<br>Tel: +44 (0) 1865-572245<br>E-mail: heather.house@admin.ox.ac.uk
**Lead NHS Trust**	Ms Heather House<br>R&D Department, Oxford University Hospitals NHS Trust<br>Joint Research Office, Block 60, Churchill Hospital Oxford OX3 7LE<br>Tel: +44 (0) 1865-572245<br>E-mail: heather.house@admin.ox.ac.uk
**Investigational Drug Supplier**<br>**Celsion Key Contacts**	Celsion Corporation, Lawrenceville, NJ, USA<br>Dr Nicholas Borys, Senior Vice President & CMO<br>Michael DeSalvo, Manufacturing & Quality<br>Lauren Musso, Project Manager, Clinical Operations

**Patient Registration**	To register a patient on the trial:<br>Please fill in the TARDOX registration form and send in to the trial office.<br>Mon-Fri 09:00-17:00 UK Time, Fax & Email 24 hours*<br>Fax: 01865 227039<br>Email: octo-TARDOX@oncology.ox.ac.uk (preferred)<br>*Faxes & emails received outside office hours will be dealt with the next working day
**Clinical Queries and Emergency Contact Details**	During office hours: Clinical Queries should be directed to the research office. The call will be passed on to the Chief Investigator or to the Clinical Fellow assigned to TARDOX.<br><br>**Out of office hours: call the Oxford University Hospital Trust Churchill and John Radcliffe switchboard on Tel: +44 (0) 1865 741 841 and ask to call the TARDOX Study Oncologist on call.**
**Trial Statistician**	Susan Dutton<br>Senior Medical Statistician and OCTRU Lead Statistician<br>Oxford Clinical Trials Research Unit<br>Centre for Statistics in Medicine<br>Nuffield Department of Orthopaedics, Rheumatology and Musculoskeletal Sciences<br>University of Oxford<br>Botnar Research Centre<br>Windmill Road<br>Oxford OX3 7LD<br>Susan.Dutton@csm.ox.ac.uk<br><br>Dr Lang'O Odondi<br>Medical Statistician<br>Oncology Clinical Trials Office (OCTO)<br>Department of Oncology & Centre for Statistics in Medicine<br>Botnar Research Centre<br>Windmill Road<br>Oxford OX3 7LD<br>Lango.Odondi@oncology.ox.ac.uk

TABLE OF CONTENTS

PROTOCOL SYNOPSIS 8
SUMMARY OF SCHEDULE EVENTS....13
KEY DEFINITIONS....15
ABBREVIATIONS....15
1 INTRODUCTION 16
1.1 BACKGROUND ON LIVER METASTASES 17
1.1.1 Epidemiology....17
1.1.2 Treatment Modalities 17
1.2 BACKGROUND ON PRIMARY LIVER CANCER....18
1.2.1 Epidemiology....18
1.2.2 Treatment Modalities 18
1.3 INVESTIGATIONAL MEDICINAL PRODUCTS USED IN THE STUDY 18
1.3.1 Lyso-Thermosensitive Liposomal Doxorubicin (LTLD, ThermoDox®) 18
1.3.2 Current ThermoDox® Trials 19
1.4 OTHER RESEARCH INTERVENTIONS 19
1.4.1 Mild Hyperthermia Using a HIFU Therapeutic Device 19
1.5 RATIONALE FOR THE STUDY 20
1.5.1 Pre-Clinical Studies....20
1.5.2 Clinical Studies....20
2 STUDY DESIGN 20
2.1 STUDY FLOW CHARTS....21
.... ***Error! Bookmark not defined.***
2.2 DURATION OF PATIENT PARTICIPATION 24
2.3 POST-TRIAL CARE AND FOLLOW-UP 24
3 OBJECTIVES AND ENDPOINTS....24
4 PATIENT SELECTION 26
4.1 ELIGIBILITY CRITERIA FOR ENTRY INTO THE MAIN STUDY 26
4.1.1 Inclusion criteria:....26
4.1.2 Exclusion criteria: 26
4.2 PROTOCOL DEVIATIONS AND WAIVERS TO ENTRY CRITERIA 27
4.3 RE-SCREENING IF PATIENT DOES NOT MEET INCLUSION/EXCLUSION CRITERIA FIRST TIME ROUND 27
4.4 PATIENT REGISTRATION PROCEDURE....27
5 TRIAL ASSESSMENTS AND PROCEDURES....28
5.1 SCREENING (D-30 TO D-2)....28
5.1.1 Optional Two Stage Screening 28
5.1.2 Informed Consent....28
5.1.3 Screening Assessments....29
5.2 EVALUATIONS DURING THE INTERVENTION (D-1 TO D2) 30
5.3 FOLLOW-UP AND END OF STUDY EVALUATIONS (WITHIN 60 DAYS)....31
EVALUATIONS ON EARLY WITHDRAWAL 32
5.4 TRIAL PROCEDURES 32
5.4.1 Preparation for Intervention 32

5.4.2 Intervention 32
5.4.3 Focused Ultrasound Procedure 35
5.4.4 Passive Acoustic Monitoring 35
5.4.5 Participant Monitoring 35

6 EARLY PATIENT WITHDRAWAL 35

6.1 PATIENT EVALUABILITY AND REPLACEMENT 36

7 SAMPLES FOR LABORATORY ANALYSIS 36

7.1 SAMPLES TO BE ANALYSED IN LOCAL TRUST'S LABORATORIES 36
7.1.1 Diagnostic Laboratories 36
7.1.2 Pathology 36
7.2 SAMPLES TO BE SENT TO AND ANALYSED IN A CENTRAL LABORATORY 36
7.3 PHARMACOKINETIC ASSAYS 36
7.4 PHARMACODYNAMIC ASSAYS 37
7.5 SUMMARY OF SAMPLES/ASSAYS TO BE TAKEN DURING THE STUDY 37
7.6 SAMPLES FOR BIOBANKING 37
7.7 LABELLING AND CONFIDENTIALITY OF SAMPLES SENT 37
7.8 CLINICAL REPORTING OF EXPLORATORY RESEARCH ASSAY RESULTS 37
7.9 TRIAL SAMPLE RETENTION AT END OF STUDY 37
7.10 WITHDRAWAL OF CONSENT FOR SAMPLE COLLECTION AND/OR RETENTION 37

8 INVESTIGATIONAL MEDICINAL PRODUCTS (IMPS) 37

8.1 NAME OF IMPS 37
8.2 TREATMENT DOSE 37
8.3 DURATION OF TREATMENT 38
8.4 MANAGEMENT OF DRUG ADMINISTRATION 38
8.5 SPECIAL PRECAUTIONS 38
8.6 ACTUAL VERSUS IDEAL BODY WEIGHT 38
8.7 DOSE MODIFICATION 38
8.8 CALCULATING AND RECALCULATING BSA/DOSES 38
8.9 DOSE CAPPING 39
8.10 DOSE-BANDING 39
8.11 COMPLIANCE 39
8.12 MANAGEMENT OF OVERDOSE 39

9 OTHER TREATMENTS (NON-IMPS) 39

9.1 FOCUSED ULTRASOUND 39
9.2 BACKGROUND SYSTEMIC THERAPY 39
9.3 SUPPORT MEDICATION 39
9.4 CONCOMITANT MEDICATION AND NON-DRUG THERAPIES 40
9.5 PROHIBITED THERAPIES 40
9.6 (POTENTIAL) DRUG INTERACTIONS 40

10 RADIOTHERAPY 40

11 DRUG MANAGEMENT 40

11.1 DRUG SUPPLIES 40
11.2 DRUG ORDERING 41

11.3 IMP RECEIPT 41
11.4 HANDLING AND STORAGE 41
11.5 LABELLING 41
11.6 DOSING DISPENSING 42
11.7 DRUG ACCOUNTABILITY 42
11.8 DRUG RETURNS FROM PATIENTS 42
11.9 DRUG DESTRUCTION 42
11.10 OCCUPATIONAL SAFETY 42

12 EVALUATION OF RESPONSE 42

12.1 MEASUREMENT OF DISEASE FOR SOLID TUMOUR 43
12.2 TUMOUR ASSESSMENT 43
12.2.1 Baseline evaluations 43
12.2.2 Evaluations during treatment and at off-study 43

13 ASSESSMENT OF SAFETY 43

13.1 ADVERSE EVENT DEFINITIONS 43
13.2 CLINICAL LABORATORY ABNORMALITIES AND OTHER ABNORMAL ASSESSMENTS AS AES AND SAES 44
13.3 DETERMINING ADVERSE EVENT CAUSALITY 44
13.4 EXPECTED ADVERSE EVENTS 45
13.5 SUSPECTED UNEXPECTED SERIOUS ADVERSE DRUG REACTIONS (SUSARS) 47
13.6 EXPEDITED REPORTING OF SAES 47
13.7 FOLLOW-UP OF SERIOUS ADVERSE EVENTS 47
13.8 ADVERSE EVENTS THAT SHOULD BE NOTIFIED TO TRIAL OFFICE 48
13.9 REPORTING ADVERSE EVENTS ON THE CRF 48
13.10 EVENTS EXEMPT FROM BEING REPORTED AS AE/ SAES 48
13.10.1 Progression of underlying disease 48
13.10.2 Death on study 49
13.10.3 Elective admissions and supportive care 49
13.11 DEVELOPMENT SAFETY UPDATE REPORTS 49
13.12 INFORMING INVESTIGATORS OF NEW SAFETY INFORMATION 49

14 PREGNANCY 49

15 DEFINING THE END OF TRIAL 49

16 STATISTICAL CONSIDERATIONS 50

16.1 SAMPLE SIZE AND POWER 50

17 STATISTICAL ANALYSIS PLAN 50

17.1 INCLUSION IN ANALYSIS 50
17.2 SUBGROUP ANALYSIS 50
17.3 INTERIM ANALYSES 50
17.4 PROCEDURES FOR REPORTING ANY DEVIATION(S) FROM THE ORIGINAL STATISTICAL PLAN 50
17.5 FINAL ANALYSIS 50

18 TRIAL COMMITTEES 51

18.1 TRIAL MANAGEMENT GROUP (TMG) 51
18.2 DATA AND SAFETY MONITORING 51
18.3 TRIAL STEERING COMMITTEE 51

DATA MANAGEMENT 51
18.4 DATABASE CONSIDERATIONS 51
18.5 CASE REPORTS FORMS (CRFS) 51
18.6 ACCOUNTING FOR MISSING, UNUSED, OR SPURIOUS DATA. 52
19 CLINICAL STUDY REPORT 52
20 STUDY SITE MANAGEMENT 52
20.1 STUDY SITE RESPONSIBILITIES 52
20.2 STUDY SITE SET UP AND ACTIVATION 52
20.3 ARRANGEMENTS FOR SITES OUTSIDE THE UK 52
20.4 STUDY DOCUMENTATION 52
21 REGULATORY AND ETHICAL CONSIDERATIONS 52
21.1 ETHICAL CONDUCT OF THE TRIAL AND ETHICS APPROVAL 52
21.2 REGULATORY AUTHORITY APPROVAL 53
21.3 NHS RESEARCH GOVERNANCE 53
21.4 PROTOCOL AMENDMENTS 53
21.5 URGENT SAFETY MEASURES 53
21.6 TEMPORARY HALT 54
21.7 SERIOUS BREACHES 54
21.8 REPORTS: PROGRESS, SAFETY AND END OF STUDY REPORTS 54
22 EXPENSES AND BENEFITS 54
23 QUALITY ASSURANCE 54
23.1 RISK ASSESSMENT 54
23.2 ON-SITE MONITORING 54
23.3 CENTRAL MONITORING 54
23.4 AUDIT AND REGULATORY INSPECTION 55
24 RECORDS RETENTION & ARCHIVING 55
25 PATIENT CONFIDENTIALITY 55
26 STUDY FUNDING 55
27 SPONSORSHIP AND INDEMNITY 55
27.1 SPONSORSHIP 55
27.2 INSURANCE 56
27.3 CONTRACTS/AGREEMENTS 56
28 PUBLICATION POLICY 56
29 REFERENCES 56
APPENDIX 1: WHO PERFORMANCE SCALE 59
APPENDIX 2: AMERICIAN SOCIETY OF ANAESTHESIOLOGISTS (ASA) CLASSIFICATION SYSTEM ...59
APPENDIX 3: CHILD-PUGH SCORE 59

PROTOCOL SYNOPSIS

**Full Title of study:**	A proof of concept study to investigate the feasibility of targeted release of doxorubicin from lyso-thermosensitive liposomal (LTSL) doxorubicin (ThermoDox®) using ultrasound-guided focused ultrasound in patients with primary or secondary liver tumours
**Short Title:**	A Phase 1 Clinical Trial of targeted chemotherapy using focused ultrasound for liver tumours
**Trial Acronym:**	TARDOX
**Study Design:**	Phase 1 prospective non-randomised safety cohort study with all patients recruited from a single UK site (Oxford). The study has an open label design with all participants receiving ThermoDox® and FUS. The study will run in two parts. Part I is required to identify optimal FUS exposure parameters using real time thermometry data from an implanted thermometry device, such as a thermistor. Part II does not require implantation of thermometry device and is designed to more accurately reflect how the therapy would be implemented in clinical practice. All evaluable participants from both Part I and Part II will be included in the endpoint analysis.<br><br><u>Part I</u><br>For each participant, a single liver tumour will receive the intervention and follow-up imaging. The ability to achieve targeted release of doxorubicin from ThermoDox® at the tumour site will be determined using three tumour biopsies with corresponding peripheral blood samples taken during intervention and/or using a radiological method (MR-Spectroscopy).<br><br><u>Part II</u><br>For each participant, a single liver tumour will receive the intervention and follow-up imaging.. The optimal FUS exposure parameters determined in Part I will be selected for Part II interventions, accounting for differences in propagation path length identified in the Ultrasound Session. The ability to achieve targeted release of doxorubicin from ThermoDox® at the tumour site will be determined using the same methods as for Part I, with the exception of requiring only two tumour biopsies.<br><br>In the 30 days following intervention, both Part I and II participants will be followed up with a maximum of two CTs, two PET-CTs and three MRI/MR-Spectroscopy scans. There is however built in flexibility for the scans to be performed at different timepoints up to 60 days post intervention should the patient provide this optional consent and not have started another treatment. If available, other non-FUS control tumour(s) in the liver exposed to ThermoDox® alone will be selected as controls against which radiological response in the FUS-targeted tumour (target tumour) will be compared.<br><br>Detailed criteria for evaluation of efficacy and safety are described in subsequent sections.
**Patient Numbers:**	A maximum of 28 evaluable participants will be recruited to the study overall.
**Target Population:**	Adult patients with incurable (unresectable, non-ablatable-for-cure) confirmed hepatic primary (hepatocellular carcinoma or cholangiocarcinoma) or secondary (metastatic) tumour. All patients will have progressed or maintained stability on conventional chemotherapy. To be suitable, patients must have liver tumour(s) in the liver of size 1cm or greater, which are amenable to ultrasound-guided biopsy.
**Objectives:**	**PRIMARY OBJECTIVE:**<br>To determine whether targeted release of doxorubicin from ThermoDox® ('drug') using mild hyperthermia generated non-invasively by focused ultrasound (FUS) is feasible in cancer patients.<br><br>**SECONDARY OBJECTIVES:**<br>• Part I only: To determine optimal FUS exposure parameters for a range of participant Body Mass Indices (BMIs) and tumour locations within the liver

	• To assess the safety of FUS-induced mild hyperthermia for drug delivery<br>• To assess the local and systemic cytotoxic effects of ThermoDox® in this setting<br><br>**TERTIARY (EXPLORATORY) OBJECTIVES:**<br>Regarding those participants for which doxorubicin has been released by optimal FUS exposure parameters:<br>• To establish the validity of other minimally-invasive and non-invasive methods of evaluating intratumoural uptake of doxorubicin as an alternative to that used in primary endpoint:<br>a) Fluorescence microscopy of biopsy<br>b) MR-Spectroscopy<br>c) Plasma pharmacokinetics<br>• To determine whether the dose of doxorubicin released has a therapeutically significant effect on the target tumour.
**Rationale:**	The development of distant spread (metastases) is a leading cause of death in patients with advanced solid tumours. The liver is one of the most common sites for metastatic disease. Studies have shown that the treatment of distant metastases improves survival. Life expectancy for patients with metastatic colorectal cancers without treatment ranges from 5 to 9 months with a median survival after diagnosis of 6 months [1-3]. Surgical resection remains the only treatment shown to have long-term curative potential [4]. However, only a minority (10-20%) of patients are surgical candidates [5]. Systemic chemotherapy is the current treatment of choice for patients with inoperable liver metastasis. However, long-term survival is rare. The majority of primary liver cancers (hepatocellular carcinoma (HCC) and cholangiocarcioma) are unresectable and survial remains dismal [6-9]. Post-hoc analysis of the Phase III HEAT study is indicative that ThermoDox® treatment improves survival in patients with HCC, where Radiofrequency Ablation (RFA) was optimal.<br><br>This proof of concept study proposes targeted delivery of broad-spectrum cytotoxic agent (doxorubicin), via a specially formulated LTSL (ThermoDox®) activated by mild hyperthermia, as both a feasible and effective solution to the drug-delivery problem for the same systemic dose. Recent animal work performed at Oxford using ThermoDox® released using FUS has shown that increased uptake at the target site is achievable. It is proposed that this method of targeted drug delivery is clinically applicable, and may achieve increased tumour uptake and local dose for the equivalent dose of doxorubicin used in systemic chemotherapy, which has a well-established and safe toxicity profile.<br><br>Clinical studies have demonstrated:<br>1. The safety profile of ThermoDox® has been shown to be consistent with that of doxorubicin (HEAT study)<br>2. ThermoDox® treatment shows promise for improved progression-free survival and overall survival in the subset HCC of patients having optimal RFA (HEAT study)<br>3. ThermoDox® has demonstrated a dose-response effect against metastatic liver tumours [10]<br><br>If this study demonstrates successful targeted drug delivery in human subjects using ThermoDox® combined with mild-hyperthermia, this could potentially transform the future of chemotherapy in clinical practice; targeted therapy using LTSLs containing other chemotherapeutic agents triggered non-invasively by mild hyperthermia could be applied to any solid tumour.
**Primary Endpoint:**	A demonstrable two-fold increase in, or value exceeding 10µg/g of, the concentration of intra-tumoural doxorubicin at the treated tumour site following FUS-induced mild hyperthermia, in at least 50% of evaluable participants.
**Secondary Endpoints:**	1. Part I only: Achievement of the desired range of mild hyperthermia in the target tissue as monitored by the implanted thermometry device during FUS exposure

	2. Persistence of cell viability stain or percentage of cell or tissue necrosis under 30% following FUS exposure, as assessed by cytological or histological methods<br>3. Significant bone marrow suppression, deranged liver function and liver toxicity.<br>4. Adverse events up to 30 days post-intervention:<br>a. Deemed related to ThermoDox®<br>b. Deemed related to FUS
**Tertiary (Exploratory) Endpoints:**	Regarding doxorubicin released by optimal FUS exposure parameters:<br>1. Combined analysis of relevant end points to establish most effective alternative analytical method(s) for quantifying drug release:<br>a. Released biopsy samples exhibiting averaged regions of nuclear or background fluorescence levels at least twice that of a suitable control, as assessed by fluorescence microscopy<br>b. Liver MRI/MR-Spectroscopy indicating targeted delivery of doxorubicin to the tumour by demonstrating a statistically significant increase in the tumour:background ratio of signal intensity following intervention compared to baseline<br>c. A significant decrease of in total doxorubicin plasma levels over time-matched clearance levels, following FUS-induced release<br>2. Radiological evidence of tumour response at timepoints up to 60 days post-intervention in the FUS-targeted tumour and control(s), as assessed by Principles of CHOI and RECIST response evaluation using MRI and CT [11, 12] and SUV_{max} using PET-CT [13]
**Inclusion Criteria**	Patients will only be eligible for inclusion in this study if all of the following criteria apply:<br>1. Pathologically confirmed advanced solid tumour with liver metastasis suitable for intervention (as assessed by ultrasound or other radiological methods). In addition confirmed primary liver tumours (hepatocellular carcinoma or cholangiocarcinoma) can be included.<br>2. Will have progressed or remained stable on conventional chemotherapy.<br>3. Male or Female, Age ≥ 18 years.<br>4. Have life expectancy of ≥ 3 months.<br>5. Left Ventricular Ejection Fraction (LVEF) ≥ 50% on echocardiogram.<br>6. Have not received radiotherapy to the target area within the preceding 12 months.<br>7. A World Health Organisation (WHO) performance status of ≤ 1 (Appendix 1).<br>8. Able and willing to give written informed consent, indicating that they are aware of the investigational nature of this study and potential risks, and able to comply with the protocol for the duration of the study, including scheduled follow-up visits and examinations.
**Exclusion criteria**	Patients will be ineligible for recruitment to the study if any of the following conditions hold:<br>1. Have surgery or other procedure requiring general anaesthesia planned to be undertaken during the period of the study.<br>2. Have serious illnesses including, but not limited to, congestive heart failure (NYHA class III or IV functional classification); life threatening cardiac arrhythmia; or myocardial infarction or cerebral vascular accident within the last 6 months.<br>3. Have on going significant infection (chest, urine, blood, intra-abdominal).<br>4. Have uncontrolled diabetes.<br>5. Have received a life-time dose of doxorubicin > $450mg/m^2$ or a life-time dose of epirubicin > $900mg/m^2$ or any dose of both.<br>6. Pregnant or breast-feeding. In women of childbearing potential, a negative

	pregnancy test (serum) is required within 30 days prior to study intervention.<br>7. Female participants of child bearing potential and male participants whose partner is of child bearing potential who are not willing to practice an acceptable form of contraception (i.e. oral contraceptive, diaphragm, cervical cap, condom, surgical sterility) during the study and for 6 months thereafter. Women whose partner has or men who have undergone a vasectomy must use a second form of birth control.<br>8. Have any known allergic reactions to any of the drugs or liposomal components or intravenous imaging agents to be used in this study.<br>9. Have portal or hepatic vein tumour invasion/thrombosis.<br>10. Inadequate haematological and biochemical function (section 4.1.2)<br>11. Have contraindications to receiving doxorubicin including prior sensitivity (rash, dyspnoea, wheezing, urticarial or other symptoms) attributed to anthracyclines or other liposomal drugs.<br>12. Use of chemotherapy or of an investigational drug within 30 days or 5 half-lives, whichever is longer, preceding the intervention.<br>13. Have medically significant active infection.<br>14. Have Child-Pugh Class C liver disease, or Class A-B with encephalopathy and/or refractory ascites (Appendix 3).<br>15. Documented HIV positive.<br>16. Documented diagnosis of haemochromatosis.<br>17. Documented history of contrast-induced nephropathy.<br>18. Have any of the following contraindications for liver biopsy:<br>a. Suspected liver haemangioma or other vascular tumour<br>b. Tense ascites<br>c. Known cystic liver disease*<br>d. Extra-hepatic biliary obstruction*<br>(* Relative contraindications only and may be non-exclusive at discretion of the study team)<br>19. Other medical or psychiatric conditions or laboratory abnormalities that the investigator considers would make the patient a poor trial candidate.
**Trial dose and administration:**	The investigative medicinal product is ThermoDox® LTSL doxorubicin. It is supplied in 20cc vials each vial containing 30mg doxorubicin in 15ml of solution. A carton containing 4 vials is normally sufficient for one treatment of one subject.<br>One single dose of ThermoDox® with a doxorubicin concentration of 50mg/m^2 will be given during the FUS exposure session as an intravenous infusion for both Part I & II.
**Duration on study:**	30 days from intervention (Day 1) with a maximum of 60 days of radiological follow-up
**Study Procedures and frequency:**	Part I<br>Part I is concerned with optimisation of the FUS acoustic parameters. The optimal FUS exposure parameters to achieve the desired range of mild hyperthermia in the target tumour (and consequent ThermoDox® release) will be determined. A single target liver tumour will have already been identified. The participant requires insertion of a co-axial needle during intervention for monitoring and biopsy; consequently Part I is minimally invasive.<br><br>Day -1: Attend for oral premedication and for baseline radiological assessment (Liver MRI/MR-Spectroscopy, Liver pCT and whole body FDG PET-CT scans)<br><br>Day 1: Target tumour exposed to a combination of FUS and ThermoDox® under General Anaesthetic. Three liver tumour biopsies and temperature readings are taken via a single co-axial needle. Three peripheral blood samples are taken for

<table>
<tr><td colspan="2"></td><td>plasma pharmacokinetics.

Day 2: Peripheral blood samples (FBC, U&E, LFT) are taken. Repeat Liver MRI/MR-Spectroscopy is performed within 36 hours of intervention. Participant is discharged after a minimum of 24 hours of observation post-intervention.

Follow-up: Clinical reviews and repeat set of blood samples (FBC, U&E, LFT) at Days 15 and 30. A maximum of two further sets of scans (Liver MRI/MR-Spectroscopy, Liver pCT and whole body FDG PET-CT) are performed within 60 days post-intervention (one at 4 week follow-up, the other being flexible).

Part II
Part II is concerned with monitoring efficacy of the intervention (ThermoDox® release by mild hyperthermia), using the optimised FUS acoustic parameters identified in Part I. In particular, the release of doxorubicin and its therapeutic effect will be investigated. A single liver tumour will have been identified for intervention.. The participant requires insertion of a co-axial needle during intervention for a biopsy of the tumour area receiving FUS exposure for HPLC and microscopy analysis, and consequently Part II is minimally invasive.

Day -1: As for Part I.

Day 1: Target tumour exposed to a combination of FUS and ThermoDox® under General Anaesthetic. Two biopsies of the tumour receiving intervention are taken via a single co-axial needle. Peripheral blood samples are taken for plasma pharmacokinetics.

Day 2: As for Part I.

Follow-up: As for Part I.</td></tr>
<tr><td colspan="2">Patient care post-trial:</td><td>Patients will be managed as per standard of care.</td></tr>
<tr><td rowspan="2">Criteria for evaluation</td><td>Safety:</td><td>Safety and toxicity will be reported using NCI CTCAE (currently version 4.03).</td></tr>
<tr><td>Efficacy:</td><td>Efficacy will be assessed by evidencing targeted ThermoDox® release, as per the primary endpoint. In addition radiological response will be assessed as a tertiary endpoint.</td></tr>
<tr><td colspan="2">Pharmacokinetic assays:</td><td>Analysis of plasma samples to capture plasma pharmacokinetics of doxorubicin.</td></tr>
<tr><td colspan="2">Histopathology:</td><td>Histological analysis of biopsy samples obtained from the targeted liver tumour may be performed by microscopy where tissue mass allows</td></tr>
<tr><td colspan="2">No. of Study Site(s)</td><td>Single Centre UK (OXFORD)</td></tr>
<tr><td colspan="2">End of study</td><td>Last Patient Last Visit</td></tr>
<tr><td colspan="2">Publication policy</td><td>The intention is to publish this research in a specialist peer reviewed scientific journal on completion of the study. The results may also be presented at scientific meetings and/or used for a thesis. The Investigators will be involved in reviewing drafts of the manuscripts, abstracts, press releases and any other publications arising from the trial and retain final editorial control.</td></tr>
</table>

SUMMARY OF SCHEDULE EVENTS

Visit Description	Ultrasound Screening[1]		Full Screening & Main Study		Intervention				2wk Follow-up	4wk Follow-up*	Flexible Radiological Follow-up
	Consent	Screening	Consent	Screening							
Visit No.	1a	1b	2a[2]	2b[3]	3				4	5	Flexible
Visit Type	Outpatient	Outpatient	Outpatient	Outpatient	Outpatient then Inpatient from Day -1 or Day 1[4]				Outpatient	Outpatient	Outpatient
Admission Duration (days)	0	0	0	0	3				0	0	0
Day		From -40 to -2		From -30 to -2	(-7 to -1) (Pre-intervention)	-1 (Pre-intervention)	1 (Intervention Day)	2 (Recovery Day)	15	30	12-60**
Visit window (days)		After 1a		After 2a	After 2b				±3	+7 not <30	Not >60
Informed Consent	X		X								
Inclusion / Exclusion Criteria				X							
Demographics, Medical & Surgical History				X							
Concomitant medication				X						X	
Physical Exam & Vital Signs				X				X			
Height				X							
Weight				X		X					
Urinalysis ± Pregnancy Test				X							
ECG				X							
Echocardiogram[5]				X							

[1] The ultrasound screening may optionally be performed in advance of screening for the main study, or during the study screening.
[2] Study consent is usually taken during the screening visit but occasionally in advance (e.g. in clinic), but must occur before study procedures are performed outside of standard care.
[3] Best efforts will be made to ensure all screening procedures & required tests occur during a single screening visit but, but according to resources and patient preference, one or more additional screening visit(s) may occasionally be required.
[4] It is not compulsory for participants to be admitted on day -1. I.e. the patient can attend to complete pre-intervention assessments and return on day 1 to be admitted for the intervention.
[5] Echocardiogram will only be repeated if clinically indicated.

WHO PS				X							
Haematology tests[6]				X				X	X	X	
Biochemistry tests [7]				X				X	X	X	
Group & Save [8]				X							
Coagulation studies[9]				X							
Pre-operative Anaesthetic Assessment (ASA Classification)				X							
Ultrasound Session[10]		X		X							
Liver MRI/MR Spectroscopy[11]					X			X[12]		X	X
Liver pCT [13]					X					X	X
Whole Body FDG PET-CT					X					X	X
Pre-medication						X					
FUS + ThermoDox®							X				
ThermoDox® Plasma Samples							X[14]				
Tumour Biopsy Samples							X[15]				
Adverse Event Review							X	X	X	X	

* The 4 week follow-up visit will mark the end of the study and the participant will be free to consider other studies from this point, however scans may be performed after this time point in the flexible scan follow-up visit if that particant is not already enrolled on another study at that time.
** The flexible radiology follow-up visit will be chosen based on patient preference, available imaging slots and emerging radiological data. Patient visits will be minimised where possible. The flexible scan visit will be performed at least a week either side the 4 week follow-up visit, and may coincide with the 2 week follow-up visit.
In Part II, the number of follow-up MRI/MR-Spectroscopy, Liver pCT and whole body FDG PET-CT scans may be reduced, if the data from Part I informs that a more efficient (in terms of reducing patients & site requirements) schedule is possible.

---

[6] Full Blood Count (FBC); including Haemoglobin (Hb), white blood cells (WBC) with differential count (neutrophils and lymphocytes) and platelets.
[7] Biochemical profile to include U&Es, (Na+, K+, Ur, Cr), phosphate, calcium, LFTs (total protein, ALP, Albumin, ALT or AST, Bili, LDH) (validity period for bloods is 3 days).
[8] Group & Save, as standard for surgical candidates, date of valid test to be recorded on CRF, not blood group. Can alternatively be performed on d-1.
[9] Coagulation studies are performed at baseline only (PT & APTT).
[10] During the Ultrasound Session feasibility of intervention is confirmed and a target tumour will be selected by ultrasound. Only one screening ultrasound is required.
[11] MRI includes the abdomen. Measurements made using the modified RECIST 1.1 criteria.
[12] Liver MRI includes the abdomen. The first Liver MRI/MR Spectroscopy following intervention can alternatively be performed on the same day as intervention, if intervention is in the morning. It should occur within 36 hours of the completion of intervention, and ideally within 24 hours where possible.
[13] Liver pCT includes the abdomen. Measurements made using CHOI and the modified RECIST 1.1 criteria.
[14] Three blood samples for pharmacokinetics in both Phases I and II (Pre-Treatment, Unreleased drug, Released drug).
[15] Three biopsies in Part I (Pre-Treatment, Unreleased drug, Released drug), two biopsies in Part II (Released drug).

KEY DEFINITIONS

"Intervention" refers to attempted targeted release of ThermoDox® by mild hyperthermia using a focused ultrasound.

The "FUS-targeted tumour" or "target tumour" refers to the liver tumour treated with FUS following ThermoDox® delivery. This is in contrast to the RECIST definition, which defines target tumours as those measurable lesions which are followed through the patient's treatment course.

A "non-FUS control tumour" refers to a liver tumour receiving ThermoDox® alone which has been selected for radiological follow-up and comparision with the target tumour.

The "desired range of mild hyperthermia" is used in the context of ThermoDox® release in this study and is defined as achieved bulk tissue temperature in the range of 41-47°C

"Optimal FUS exposure parameters" are defined as those combinations of settings on the HIFU device (power, duty cycle and transducer motion plan) which result in an adequate temperature rise to successfully release doxorubicin from the LTSLs in the target tumour, without causing direct thermal tissue damage.

An "evaluable participant" is defined as a participant who has received FUS following delivery of ThermoDox® in Part I or II, and for whom it has been possible to evaluate the outcome of the intervention based on biopsy samples, or radiologically.

An "evaluable sample" is defined as a sample for which sufficient material was available for analysis and the analytical technique has provided quantifiable data.

ABBREVIATIONS

AE	Adverse Event
ADL	Activities of Daily Living
ADR	Adverse Drug Reaction
ALT	Alanine Transaminase
ANC	Absolute Neutrophil Count
ASA	American Society of Anaesthesiologists
AST	Aspartate Transaminase
BD	Bi Daily
BMI	Body Mass Index
CCA	Cholangiocarcinoma
CI	Chief Investigator
CR	Complete Response
CRC	Colorectal cancer
CRF	Case Report Form
CT	Computerized Tomography
CTA	Clinical Trials Authorisation
CTA	Clinical Trial Agreement
CTCAE	Common Toxicity Criteria Adverse Events
CTIMP	Clinical Trial of an Investigational Medicinal Product
DLT	Dose Limiting Toxicity
DNA	Deoxyribonucleic Acid
DSUR	Development Safety Update Report
ECG	Electrocardiogram
eCRF	Electronic Case Report Form
EMA	European Medicines Agency
EP	Early Progression
FBC	Full Blood Count
FDA	Federal Drug Administration
FDG-PET	Fluorodeoxyglucose Positron Emission Tomography
FUS	Focused Ultrasound

GCP	Good Clinical Practice
GI	Gastro-Intestinal
GMP	Good Manufacturing Practices
GP	General Practitioner
Hgb	Haemoglobin
HCC	Hepatocellular Carcinoma
HIFU	High Intensity Focused Ultrasound
HIV	Human Immunodeficiency Virus
IB	Investigators Brochure
ICH	International Conference of Harmonisation
IMP	Investigational Medicinal Product
LDH	Lactate Dehydrogenase
LVEF	Left Ventricular Ejection Fraction
LTLD	Lyso-Thermosensitive Liposomal Doxorubicin
LTSL	Lyso-Thermosensitive Liposome
mCRC	Metastatic Colorectal Cancer
MedDRA	Medical Dictionary for Regulatory Activities
MHRA	Medicines and Healthcare products Regulatory Agency
MRI	Magnetic Resonance Imaging
MTD	Maximum Tolerated Dose
NCI	National Cancer Institute
NE	Not Evaluable
NHS	National Health Service
NIMP	Non-Investigational Medicinal Product
OCTO	Oncology Clinical Trials Office
OCTRU	Oxford Clinical Trials Research Unit
OD	Once Daily
OS	Overall survival
pCT	Perfusion CT
PD	Pharmacodynamic **or** Progressive Disease
PFS	Progression Free Survival
PI	Principal Investigator
PICs	Participant Identification Centres (PICs)
PIS	Patient Information Sheet
PK	Pharmacokinetic
PO	Per Os (oral)
PR	Partial Response
QT	QT interval
QTc	Corrected QT interval
REC	Research Ethics Committee
RECIST	Response Evaluation Criteria In Solid Tumours
RFA	Radio-frequency Ablation
RR	Response Rate
SAE	Serious Adverse Event
SD	Stable Disease
SOP	Standard Operating Procedure
SUSAR	Suspected Unexpected Serious Adverse Reaction
SUV_{max}	Standardised Uptake Value Maximum [13]
TSC	Trial Steering Committee
TSL	Thermosensitive Liposome
WBC	White Blood Count

1 INTRODUCTION

This proof of concept study proposes targeted delivery of a broad-spectrum cytotoxic agent (doxorubicin), via a specially formulated LTSL (ThermoDox®) activated by mild hyperthermia, to achieve enhanced intratumoural doxorubicin concentrations for the *same systemic dose*.

1.1 Background on Liver Metastases

1.1.1 Epidemiology

The development of distant spread (metastases) is a leading cause of death in patients with malignant solid tumours. Lung, breast and colorectal cancer are, in that order, the three most common cancers worldwide. These three cancer sites account for 40% of the world's total cases diagnosed, amounting to 4 million a year. They are also the leading cause of cancer death, resulting in almost 2.5 million deaths each year worldwide (International Agency for Research on Cancer: Fact Sheet, 2012).

The liver is one of the most common sites for metastatic disease. Liver metastases are diagnosed in 10–25% of patients at the time of resection of their primary colorectal tumour and, eventually, up to 70% of patients develop liver metastases [15, 16]. In patients with breast cancer, 15% of patients have liver metastasis at the time of diagnosis, and as many as 50% of patients with stage IV disease will develop metastases [17-21]. The most common sites for metastatic spread of breast cancer are the lung, bone and liver (50-71%) [22]. Approximately a third of patients with non-small cell lung cancer have liver metastases at the time of diagnosis [23].

In a significant proportion of patients, the liver is the only site of distant disease. In 30% of the patients with colorectal and breast cancer, the metastases are confined to the liver [15, 16, 24]. Studies have shown that the treatment of distant metastases improves survival. Life expectancy for patients with colorectal metastases without treatment is dismal and ranges from 5 to 9 months with a median survival after diagnosis of 6 months [1-3].

The presence of liver metastasis is an important prognostic factor in patients with lung cancer [25] with a reduction in the five-year survival rate from 36% to only 1% [25, 26]. Although recent advances in breast cancer treatment have significantly improved survival rates in recent years, metastatic breast cancer as a whole has a median survival time of two years which is largely unchanged [22]. Life expectancy of breast cancer patients with liver metastases improves from 3-6 months without treatment to 15-32 months with treatment [18, 20, 21, 27].

1.1.2 Treatment Modalities

Surgical resection remains the gold standard in the treatment of colorectal liver metastasis, as it is the only treatment shown to have long-term curative potential [4]. A large series of over one thousand patients followed up after liver resection for colorectal metastases demonstrated an operative mortality rate of 2.8% and a five-year survival of 37% for resections that involved a lobectomy or more [28]. However, only a minority (10-20%) of patients are surgical candidates due to either the cancer being too locally advanced, extra-hepatic, or being unfit for surgery due to co-morbidities [5]. Furthermore, the majority of patients undergoing liver resection develop new, non-resectable hepatic or extrahepatic metastases after hepatic resection [29, 30]. With the advent of neoadjuvant chemotherapy it is possible to convert some previously inoperable patients to the resectable group. A large trial of neoadjuvant systemic chemotherapy for colorectal liver metastasis looked at 330 patients with primarily unresectable liver disease from a cohort of 434 patients referred over a six year period. Of this group, 16% responded and went on to have liver resections with a comparable survival benefit to that of a primary liver resection (40% at 5 years). However, of the majority group with primarily unresectable tumours the vast majority (84%) remained inoperable [31].

Systemic chemotherapy remains the treatment of choice for patients with inoperable liver metastasis. Despite advances in systemic chemotherapy it remains a palliative treatment, as long-term survival is rare. In patients with liver metastasis from colorectal cancer, systemic chemotherapy with 5-fluorouracil (5-FU) has a response rate of only 20% with a median response duration of 6 months [32]. In the same patient group, combined chemotherapy regimens, such as 5-FU and leucovorin, have improved the median overall survival to approximately 12 months [33, 34] but with a trade-off of increased systemic toxicity.

Minimally invasive or non-invasive techniques in the treatment of patients with radiologically detectable and discrete metastatic liver disease are in demand, driven by surgical inoperability of the majority of liver metastases. Such non-invasive techniques are also in increasing demand among an ageing population. Ablative technologies are now becoming established and are showing encouraging outcomes for the treatment of metastatic liver disease. Such technologies are primarily being employed for palliation of patients with inoperable liver tumours who are unresponsive to chemotherapy. Modalities of ablative therapy include radiofrequency and microwave ablation, high intensity focused ultrasound (HIFU), laser and cryoablation. Overall, such techniques have the advantage over hepatic resection of shorter length of hospital stay and lower complication rates but the disadvantage of less proven survival benefits, although much clinical research activity is ongoing in this regard. Most of the current literature for ablative

therapies relates to treatment of hepatocellular carcinoma (HCC) rather than metastatic liver disease and to date there are no trials which compare outcomes between the various ablative technologies [35].

Trans-arterial techniques offer an alternative to ablative techniques. However no survival benefit has been shown in the treatment of colorectal metastases with embolization alone [36, 37] and several Phase III trials have failed to demonstrate a survival benefit in hepatic arterial chemotherapy over systemic chemotherapy [38]. Hepatic artery chemoembolization delivers a dual ischaemic and cytotoxic insult with theoretical benefits beyond either of the individual components of treatment [38]. However, multiple chemoembolization procedures are often required and reports of treatment response vary greatly among available studies. A previous study included 30 patients who received between one and three chemoembolization procedures using a combination of cytotoxic agents. Radiological response was seen in 63% of the cases but all responses were transient. Median survival was extended to 29 months after initial diagnosis of liver metastasis. Mild-to-moderate toxicity was seen and ‘post-embolization syndrome’ (fever, right upper quadrant pain, nausea and vomiting) was common [38].

Although targeted treatment of liver metastases with ablative or trans-arterial techniques allows localized, minimally invasive therapy without significant toxicity, the majority of patients with metastatic liver disease from malignant solid tumours are unsuitable for these therapies; thus palliative chemotherapy has been the mainstay of treatment. Nevertheless, the availability of these ablative modalities has stimulated interest in developing an additive or synergistic combination therapy for patients with metastatic disease using chemotherapy and ablation. The treatment of colorectal liver metastases with Radio-Frequency Ablation (RFA) provides palliation by extending life expectancy to a median survival of 36 months [39], representing a significant improvement over chemotherapy alone. However, such treatments must be radiologically amenable to accurate targeting of the tumour or tumours and thus only a limited number of metastatic tumours in the liver can be successfully treated by RFA.

1.2 Background on Primary Liver Cancer

1.2.1 Epidemiology

Primary liver cancers are the third highest cause of cancer-related death globally [40]. Although incidence is lower in the UK, it is increasing, with around 4300 new cases per year at present. In England, primary liver cancers account for around 1% of all cancer cases and have a 20% survival rate at one year (Cancer Research UK Liver Cancer: Key Facts, 2014). HCC, also known as malignant hepatoma, is the most commonly occurring primary liver cancer, accounting for 45% of cases in England (National Cancer Intelligence Network: Trends in incidence of primary liver cancer subtypes), and has risk factors including hepatitis B and C and cirrhosis. Cholangiocarcinoma (CCA), cancer of the biliary tract, accounts for 40% of primary liver cancers in England and is the most common cause of death from primary liver cancer in the UK, where it has an annual mortality rate of 1500 [41].

1.2.2 Treatment Modalities

Available treatment options for primary liver cancers largely mirror those previously discussed for metastatic liver tumours, and are dependent on staging. In HCC, again resection is the gold standard for localised disease, but is not an option in cirrhosis, often the underlying cause for HCC. Consequently there is the additional option for total hepatectomy and liver transplant in cirrhosis, should a suitable donor be found. Early diagnosis and treatment of HCC can achieve survival rates of 60-70%. However, due to a lack of alternative treatment options, prognosis in advanced disease or progressive disease following local therapy is dismal [6-8]. Sorafenib has been shown to increase median survival in those with advanced HCC by almost three months over placebo in a Phase III trial of over 600 patients [42]. In the case of CCA, again surgery offers the only chance of cure, but the majority of cases are inoperable due to presence of extra-hepatic metastasis at diagnosis. A study looking at survival rates in CCA diagnosed in European adults between 1990-1999 demonstrated age-standardised five-year relative survival rate of around 12% [9]. A recent UK study has demonstrated a six-month progression free survival of 57% using combination chemotherapy [43].

1.3 Investigational Medicinal Products used in the study

1.3.1 Lyso-Thermosensitive Liposomal Doxorubicin (LTLD, ThermoDox®)

ThermoDox® (or Lyso-Thermosensitive Liposomal Doxorubicin – LTLD) is being developed within an Investigational New Drug programme in the USA by Celsion Corporation (Celsion). Celsion will provide ThermoDox® as the Investigational Medicinal Product (IMP) for this study. ThermoDox® is a liposomal drug delivery system that releases a broad-spectrum cytotoxic drug (doxorubicin) when exposed to mild hyperthermia as the trigger. Drug release occurs

in the range of 39-45°C and maximal activation of the LTSLs occurs around 42°C. In this study, using FUS, we aim to achieve the desired range of mild hyperthermia for ThermoDox® release.

1.3.2 Current ThermoDox® Trials

The ongoing clinical program is currently investigating ThermoDox in two indications: hepatocellur carcinoma and recurrent chest wall breast cancer.

The lead clinical study is *A Phase III, Randomized, Double Blind, Dummy-Controlled Study of ThermoDox® (Lyso-Thermosensitive Liposomal Doxorubicin-LTLD) in Hepatocellular Carcinoma (HCC) using standardized Radiofrequency Ablation (sRFA) treatment time ≥ 45 minutes for intermediate size solitary lesions ≥ 3 cm to ≤ 7 cm.* (Celsion Protocol 104-13-302, The OPTIMA Study). This trial is designed to evaluate an optimized RFA dwell time ≥ 45 minutes based on results from the prior clinical program in HCC. The OPTIMA study was initiated in June 2014 and is designed to enroll about 550 patients. The primary endpoint is overall survival with progression free survival as a secondary endpoint.

The HEAT Study, a pivotal Phase III trial enrolled 701 newly diagnosed HCC subjects with unresectable hepatocellular carcinoma (HCC) lesions, with at least one ≥ 3.0 cm and none > 7.0 cm. The study demonstrated that ThermoDox can be safely administered in this patient population but failed to meet the primary endpoint of Progression Free Survival and Overall Survival. A *Post hoc* analysis of the data from the HEAT Study was undertaken in order to better understand the results and to identify any subgroups that may have benefited from the treatment. The analysis suggests that ThermoDox efficacy for the treatment of single lesions is greatly improved when RFA dwell time is ≥ 45 minutes which has formed the basis of the hypothesis driving the current prospective pivotal study OPTIMA.

A Phase II open label study in subjects with unresectable loco-regional recurrent breast cancer of the chest wall (RCW) administered ThermoDox in conjuction with mild local hyperthermia using microwave or ultrasound heating devices is on-going. The study has completed enrollment of 17 subjects to evaluate bioequivalence, safety and efficacy of ThermoDox manufactured at two manufacturing sites. Additionally, a Phase II open label study in subjects with RCW administering ThermoDox in conjuction with mild local hyperthermia and radiation therapy will enroll patients throughout Europe and Israel.

The most frequent adverse events in patients treated with ThermoDox® at a 50 mg/m^2 dose are myelosuppression (60%), haematological adverse event (related or unrelated) (45%) and alopecia (40%). Other toxicities reported in greater than 5% of patients include pyrexia, fatigue, nausea and decreased appetite. To date, no significant hypersensitivity or anaphylactoid reactions have been reported in clinical studies (refer to the ThermoDox® Investigator's Brochure [44], referred to as the 'ThermoDox® IB' from hereon).

Of particular relevance to this study, the safety profile of ThermoDox® in the HEAT study has been shown to be consistent with that of doxorubicin, with neutropenia being the most important adverse event. ThermoDox® was well tolerated with no unexpected serious adverse events.

1.4 Other research interventions

1.4.1 Mild Hyperthermia Using a HIFU Therapeutic Device

HIFU therapeutic devices employ focused ultrasound, usually through the medium of water, to provide a thermal effect, typically ablation, at a precise anatomical location (section 5.4.3). Although less commonly performed for hepatic metastatic disease than either RFA or microwave ablation, ablative HIFU has the advantage of being completely non-invasive. The University of Oxford and the Oxford University Hospitals NHS Trust have extensive clinical HIFU experience. The first extracorporeal HIFU device in Europe was used for a study performed at the Churchill Hospital between 2002 and 2004 [45]. The study used an ultrasound-guided HIFU system (Model-JC Tumour Therapy System, HAIFU Technology Company Ltd, Chongqing, China) to treat 30 patients with tumour deposits in the kidney or liver, and demonstrated both safety and efficacy. This was followed by the first clinical trial of HIFU ablation of tumours in the Western world carried out in Oxford [46]. This non-randomised Phase II clinical trial compared radiological response against histological findings and again demonstrated safety and feasibility for non-invasive treatment of solid tumour deposits. These studies have led to CE-marking of the JC and JC-200 treatment devices for non-invasive treatment of solid tumours such as liver cancer, kidney tumours, breast cancer, bone tumours, pancreatic tumours and uterine fibroids.

Experiments have been performed in our laboratory using gel based tissue-mimicking model phantoms, demonstrating that the temperature rises required for release of the drug from the LTSLs can be achieved using a known clinical ultrasound therapy system at a fraction of the power used for thermal ablation. The resultant bulk heating effect on the model is well below the established thresholds for thermal cell death. It follows that drug release from LTSLs under mild hyperthermia is theoretically achievable without any significant thermal damage to the tissue.

In this study, using focused ultrasound (FUS) to induce mild hyperthermia in participants under controlled limits with real-time temperature monitoring, we aim to remain well below thresholds for ablation or cell death of the targeted tumour cells by thermal damage. The aim of using FUS in this study is to investigate the potential for triggered drug-release by mild hyperthermia at the targeted tumour site and not its direct ablative effect as a cancer treatment itself.

1.5 Rationale for the study

To date, purely pharmacological approaches have failed to address what is essentially a three-fold challenge: (i) to deliver therapeutically significant concentrations of active agents to the tumour vasculature while minimizing off-target effects; (ii) to release the therapeutic agent 'on-demand' at the target site; and, (iii) to improve the distribution and spread of the therapeutic agent against the intra-tumoural pressure gradient in order to achieve a therapeutically relevant concentration throughout the tumour.

1.5.1 Pre-Clinical Studies

Recent animal work performed at Oxford using ThermoDox® released using FUS has shown that increased uptake at the target site is achievable. It is proposed that this method of targeted drug delivery is clinically applicable, and may achieve increased tumour uptake and local dose for the equivalent dose of doxorubicin used in systemic chemotherapy, which has a well-established and safe toxicity profile.

1.5.2 Clinical Studies

Clinical studies have demonstrated:

1. The safety profile of ThermoDox® has been shown to be consistent with that of doxorubicin (HEAT study)
2. ThermoDox® treatment shows promise for improved progression-free survival and overall survival in the subset of HCC patients having optimal RFA (HEAT study)
3. ThermoDox® has demonstrated a dose-response effect against metastatic liver tumours [10]

If this study demonstrates successful targeted drug delivery in human subjects using ThermoDox® combined with mild-hyperthermia, this could potentially transform the future of chemotherapy in clinical practice; targeted therapy using LTSLs containing other chemotherapeutic agents triggered non-invasively by mild hyperthermia could be applied to any solid tumour.

2 STUDY DESIGN

Phase 1 prospective non-randomised open label single site study to determine whether targeted release of doxorubicin from ThermoDox® ('drug') using mild hyperthermia generated non-invasively by FUS is possible in cancer patients. All participants will receive ThermoDox® and FUS.

The study will run in two parts. Part I will identify optimal FUS exposure parameters for a range of patient BMIs and tumour locations within the liver using real time thermometry data from an implanted thermometry device, such as a thermistor or thermocouple. After at least 5 participants have had the intervention using real-time thermometry, data will be reviewed by the Trial Management Group (TMG) to confirm readiness to proceed without real-time thermometry (Part II). Part II, which does not require implantation of a thermometry device, is designed to reflect how the therapy would be implemented in clinical practice. All evaluable participants from both Part I and Part II will be included in the endpoint analysis.

2.1 Study Flow Charts

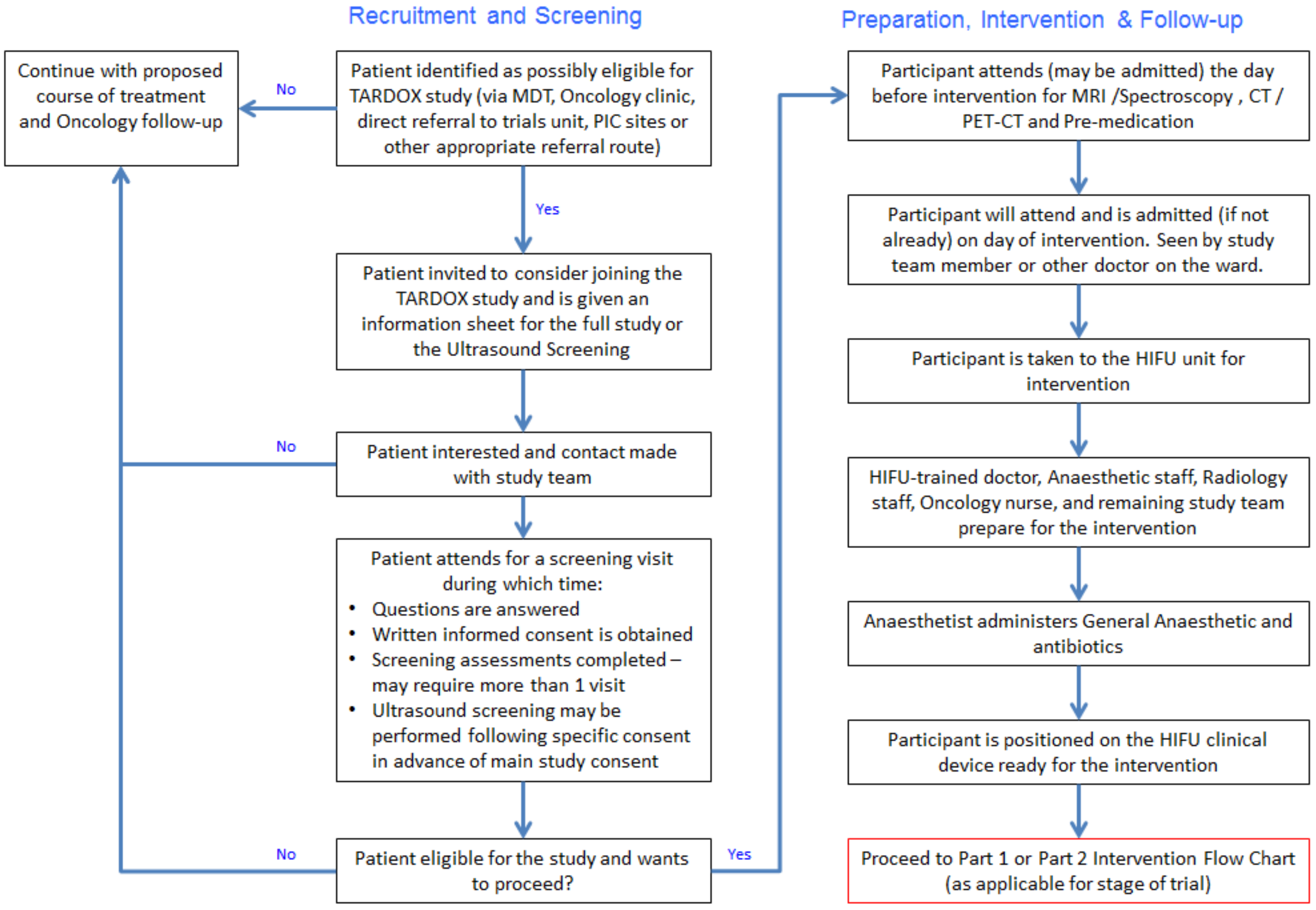

Figure 1: Recruitment, Screening and Intervention Preparation flow chart. Note that the ultrasound screening session can optionally be consented for in advance of the main study consent.

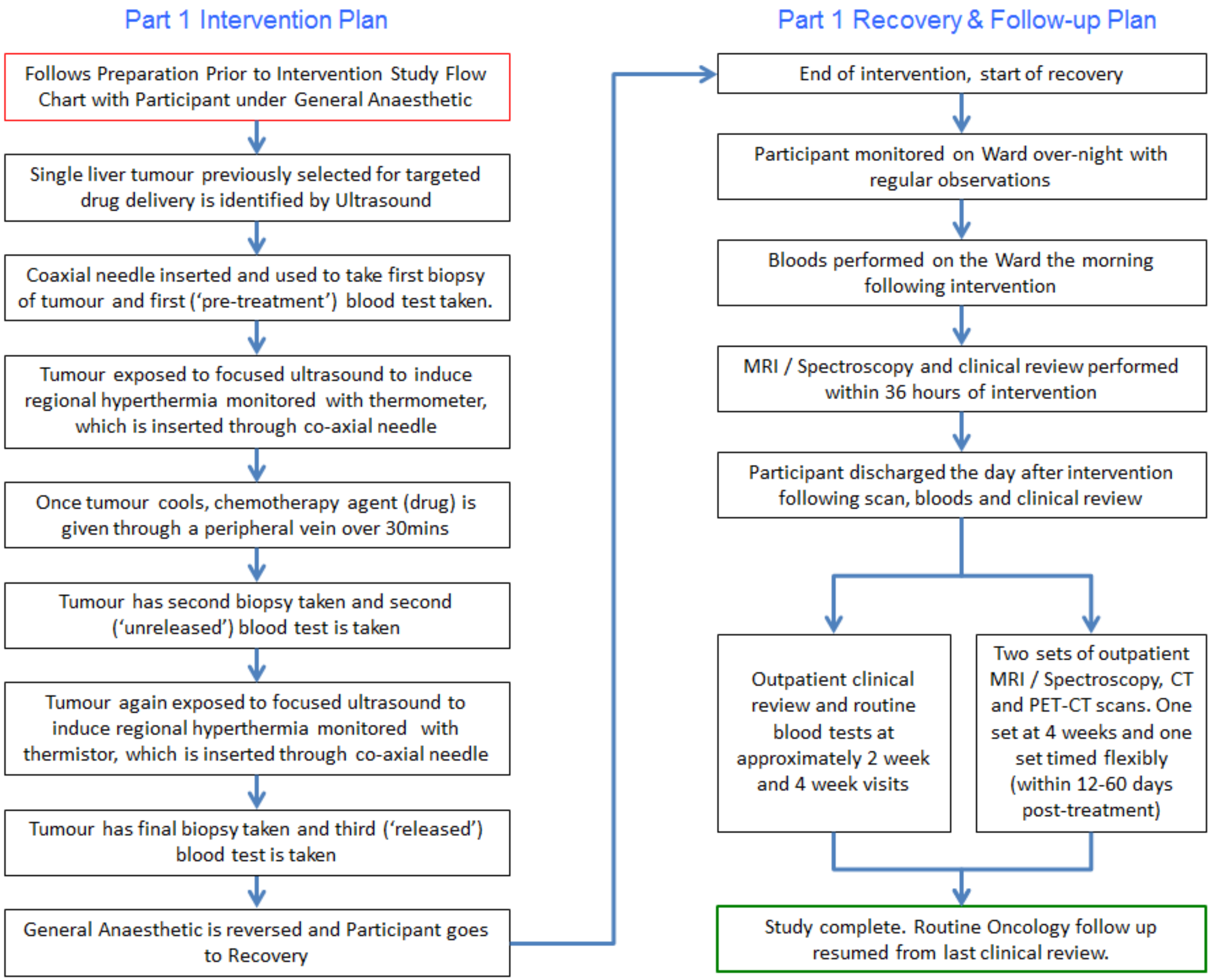

Figure 2: Part I Intervention Plan, Recovery & Follow-up flow chart

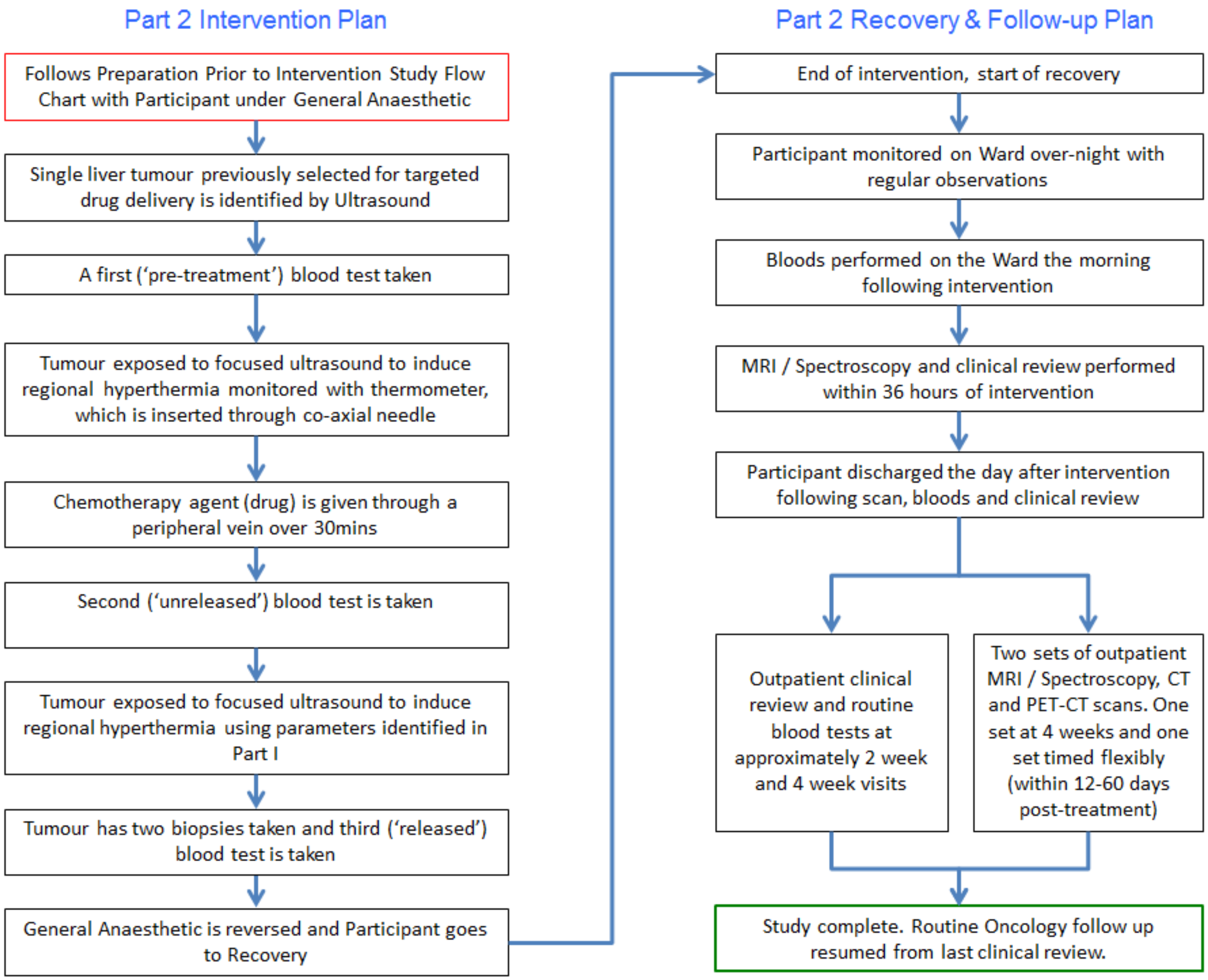

Figure 3: Part II Intervention Plan, Recovery & Follow-up flow chart

2.2 Duration of patient participation

Participants will be in the study for less than three months following entry on study and the participant will be followed up clinically for a maximum of 37 days and radiologically for a maximum of 60 days after intervention (Day 1).

2.3 Post-trial care and follow-up

Following the completion of the study, patients will receive standard care until disease progression or death.

3 OBJECTIVES AND ENDPOINTS

The objectives and endpoints for the study are detailed in the following tables. Only evaluable samples will be included in endpoint analysis. All endpoints will be evaluated at the end of Phase II.

The following qualifiers (*,†, ‡ and §) apply to Table 1, Table 2 and Table 3:

* In Part II the Unreleased biopsy is not performed and therefore the average value for all evaluable tumours receiving intervention in Part I is used as a comparison for the two-fold increase from Unreleased to Released biopsy.

† Duration refers to the completion of sample processing but not necessarily the sample analysis. The sample may be stored in a fixed or frozen state and analysed at some time period beyond the specified duration, for example to facilitate batch processing.

‡ Analytical method will only be performed where tissue mass allows. It is expected at most one technique can be performed in addition to the primary endpoint for a given sample. Where biopsy mass falls short, the primary endpoint will be prioritised.

§ Must be shown in at least 50% of evaluable participants.

Primary Objective	Primary Endpoint	How and when data is captured
To determine whether targeted release of doxorubicin from ThermoDox® ('drug') using mild hyperthermia generated non-invasively by focused ultrasound (FUS) is feasible in cancer patients	A demonstrable two-fold increase in*, or value exceeding 10µg/g of, the concentration of intra-tumoural doxorubicin at the treated tumour site following FUS-induced mild hyperthermia §<br><br>Quantification of intra-tumoural drug release will be achieved by direct analysis of the intra-tumoural biopsy samples using standardised curve(s)	Analytical chemistry performed on a recorded mass of biopsy sample processed within 12 hours of intervention†

Table 1: Primary Objective and Endpoints for the TARDOX Study

Secondary Objectives	Secondary Endpoints	How and when data is captured
Part I only: To determine optimal FUS exposure parameters for a range of participant Body Mass Indices (BMIs) and tumour locations within the liver	Achievement of the desired range of mild hyperthermia in the target tissue as monitored by the implanted thermometry device during FUS exposure	Real-time thermometry monitoring during intervention
To assess the safety of FUS-induced mild hyperthermia for drug delivery	1. Persistence of cell viability stain or percentage of cell or tissue necrosis under 30% following FUS exposure, as assessed by cytological or histological	1. Cytological or histological analysis of biopsy samples processed within 96hr of intervention†

	methods[16] ‡,§<br><br>2. Adverse events deemed related to FUS up to 30 days post-intervention	2. Adverse event recording for 30 days post-intervention
To assess the local and systemic cytotoxic effects of ThermoDox® in this setting	1. Significant bone marrow suppression, deranged liver function and liver toxicity<br><br>2. Adverse events deemed related to ThermoDox® up to 30 days post-intervention	1. Grade 3 and 4 laboratory results from blood tests at Day 1 and Day 15 post intervention<br><br>2. Adverse event recording for 30 days post-intervention

Table 2: Secondary Objectives and Endpoints for Parts I & II of the TARDOX Study

Regarding participants for which doxorubicin has been released by optimal FUS exposure parameters:

Tertiary (Exploratory) Objectives	Tertiary (Exploratory) Endpoints	How and when data is captured
1. To establish the validity of other minimally-invasive and non-invasive methods of evaluating intratumoural uptake of doxorubicin as an alternative to that used in primary endpoint	Combined analysis of relevant end points to establish most effective alternative analytical method(s) for quantifying drug release	See below
a. Fluorescence microscopy of biopsy	Released biopsy samples exhibiting regions of nuclear or background fluorescence levels at least twice that of a suitable control, as assessed by fluorescence microscopy ‡,§	Microscopic analysis of biopsy samples processed within 96hr of intervention†
b. MR-Spectroscopy	Liver MRI/MR-Spectroscopy indicating[17] targeted delivery of doxorubicin to the tumour by demonstrating a statistically significant increase in the tumour:background ratio of signal intensity following intervention compared to baseline	MRI/MR-Spectroscopy signal within 36 hours of intervention
c. Plasma pharmacokinetics	A statistically significant decrease in total doxorubicin plasma levels over time-matched clearance levels, following HIFU-induced release	Analytical chemistry of plasma samples processed within 12hr of intervention†
2. To determine whether the dose of doxorubicin released has a therapeutically significant effect on the target tumour	Radiological evidence of tumour response at timepoints over 30 up to 60 days in the FUS-targeted tumour and control(s), as assessed by principles of CHOI and RECIST response evaluation using MRI and CT [11, 12] and SUV_{max} using PET-CT [13] §	Scans to be performed within 60 days of intervention.

Table 3: Tertiary (Exploratory) Objectives and Endpoints for Parts I & II of the TARDOX Study

[16] In some necrotic tumours, base line necrosis may be high (e.g. >30%) even prior to treatment. Consequently an increase in necrosis by no more than 25% over that of a suitable control can be used as an alternative endpoint.
[17] The spectroscopy signal analysed may not be doxorubicin itself; metabolites or breakdown products of ThermoDox® may be utilized.

4 PATIENT SELECTION

Written informed consent must be obtained before any study specific procedures are performed.

4.1 Eligibility criteria for entry into the main study

The Investigator will determine patient eligibility based on the following inclusion/exclusion criteria.

4.1.1 Inclusion criteria:

Patients will only be eligible for inclusion in this study if all of the following criteria apply:

1. Pathologically confirmed advanced solid tumour with liver metastasis suitable for intervention (as assessed by ultrasound or other radiological methods). In addition confirmed primary liver tumours (hepatocellular carcinoma or cholangiocarcinoma) can be included.
2. Will have progressed or remained stable on conventional chemotherapy.
3. Male or Female, Age ≥ 18 years.
4. Have life expectancy of ≥ 3 months.
5. Left Ventricular Ejection Fraction (LVEF) ≥ 50% on echocardiogram.
6. Have not received radiotherapy to the target area within the preceding 12 months.
7. A World Health Organisation (WHO) performance status of ≤ 1 (Appendix 1).
8. Able and willing to give written informed consent, indicating that they are aware of the investigational nature of this study and potential risks, and able to comply with the protocol for the duration of the study, including scheduled follow-up visits and examinations.

4.1.2 Exclusion criteria:

Patients will be ineligible for recruitment to the study if any of the following conditions hold:

1. Have surgery or other procedure requiring general anaesthesia planned to be undertaken during the period of the study.
2. Have serious illnesses including, but not limited to, congestive heart failure (NYHA class III or IV functional classification); life threatening cardiac arrhythmia; or myocardial infarction or cerebral vascular accident within the last 6 months.
3. Have on going significant infection (chest, urine, blood, intra-abdominal).
4. Have uncontrolled diabetes.
5. Have received a life-time dose of doxorubicin > 450 mg/m^2 or a life-time dose of epirubicin > 900 mg/m^2 or any dose of both.
6. Pregnant or breast-feeding. In women of childbearing potential, a negative pregnancy test (serum) is required within 30 days prior to study intervention.
7. Female participants of child bearing potential and male participants whose partner is of child bearing potential who are not willing to practice an acceptable form of contraception (i.e. oral contraceptive, diaphragm, cervical cap, condom, surgical sterility) during the study and for 6 months thereafter. Women whose partner has or men who have undergone a vasectomy must use a second form of birth control.
8. Have any known allergic reactions to any of the drugs or liposomal components or intravenous imaging agents to be used in this study.
9. Have portal or hepatic vein tumour invasion/thrombosis.
10. Inadequate Haematological and Biochemical indices:

Lab Test	Exclude if:
INR	> 1.5 times the institution's upper normal limit (UNL)*
Absolute neutrophil count	<1,500/mm^3 (or < 1.5 (10^9/L))
Platelet count	< 60,000 /mm^3 (or <60 (10^9/L))
Hgb	< 9.0 g/dL**
Serum creatinine, or<br>Calculated creatinine clearance (CrCl)	≥ 2.5 mg/dL (or ≥ 221 µmol/L)<br>≤ 25.0 mL/min.
Serum bilirubin	> 3.0 mg/dL (or >51 µmol/L)

Serum albumin	< 2.8 g/dL (or <28 g/L)

* Except in patients who are therapeutically anticoagulated for medical conditions unrelated to cancer such as atrial fibrillation. Subjects may be re-screened after the condition is treated or anticoagulant is withheld

** Unless the haemoglobin value has been stable, the patient is cardiovascularly stable, asymptomatic, and judged to withstand the FUS procedure

11. Have contraindications to receiving doxorubicin including prior sensitivity (rash, dyspnoea, wheezing, urticarial or other symptoms) attributed to anthracyclines or other liposomal drugs.
12. Use of chemotherapy or of an investigational drug within 30 days or 5 half-lives, whichever is longer, preceding the intervention.
13. Have medically significant active infection.
14. Have Child-Pugh Class C liver disease, or Class A-B with encephalopathy and/or refractory ascites (Appendix 3).
15. Documented HIV positive.
16. Documented diagnosis of haemochromatosis.
17. Documented history of contrast-induced nephropathy.
18. Have any of the following contraindications for liver biopsy:
    a. Suspected liver haemangioma or other vascular tumour
    b. Tense ascites
    c. Known cystic liver disease*
    d. Extra-hepatic biliary obstruction*

    (* Relative contraindications only and may be non-exclusive at discretion of the study team)
19. Other medical or psychiatric conditions or laboratory abnormalities that the investigator considers would make the patient a poor trial candidate.

4.2 Protocol deviations and waivers to entry criteria

Protocol adherence is a fundamental part of the conduct of a clinical study. Changes to the approved protocol need prior approval unless for urgent safety reasons.

Investigators must contact the TARDOX Trial Office to obtain guidance and/or clarification as necessary if unsure whether the patient satisfies all the entry criteria and to clarify matters of clinical discretion. The trial office will contact the chief investigator or clinical coordinators as necessary. Requests for protocol waivers to enter a patient that is clearly ineligible will not be granted.

Investigators should not deviate from the protocol for the management of enrolled subjects deliberately unless essential to protect the rights or safety of the individual. Examples might include the addition or deletion of tests, dosing, duration of treatment etc. It may be necessary to withdraw the patient from further study. All deviations should be fully documented/ justified and reported to the trial office without delay. Deviations should be discussed with the trial office and/or the Chief Investigator prior to being actioned where possible.

4.3 Re-screening if patient does not meet inclusion/exclusion criteria first time round

If a patient does not meet the inclusion/exclusion criteria first time round, he/she can be re-screened. Patients who fail at re-screening are ineligible for the TARDOX study and may not be re-screened again.

4.4 Patient registration procedure

Participants will be recruited from patients referred to oncology services for the management of metastatic cancer from solid tumour primaries. A screening log must be kept of all patients considered for the study including any that are subsequently excluded; the reason for exclusion must be recorded on this form. A copy of the screening log should be sent to the trial office on request, but without patient identifiers. The original must be retained on site. Before entering a patient onto the study the Principal Investigator or designee will confirm eligibility. If in any doubt the Chief Investigator must be consulted before entering the patient.

A completed patient registration / eligibility checklist along must be sent to the TARDOX Trial Office. Each patient must be registered with the TARDOX Trial Office using this Registration Form and receive a unique trial number by the TARDOX Trial Team.

Patients should be registered by the site by faxing the registration form to the TARDOX Trial Office using fax number **01865 227039**. Alternatively the scanned Registration Form can be emailed to: octo-TARDOX@oncology.ox.ac.uk. The Fax/Email service is available 24 hours a day (Faxes/emails received outside office hours, 09:00 – 17:00 Monday to Friday, will be dealt with the next working day). Registration Forms will be provided in the Investigator Site File. Following registration, the site will be notified by fax or email that a patient has been successfully entered into the trial and of the patient's unique trial number.

The original Registration Form should be passed to the TARDOX Trial Office once complete.

5 TRIAL ASSESSMENTS AND PROCEDURES

Please refer to the Summary Schedule of Events given at the front of this protocol. Details of all protocol evaluations and investigations must be recorded in the patient's medical record for extraction onto the CRF.

5.1 Screening (d-30 to d-2)

The following must be performed within days -30 to -2, where day -1 is the day of admission for intervention. The exception is the Ultrasound Session (section 5.1.3.1) which can take place as early as day -40 if separate consent is obtained. Tests performed as part of routine standard of care need only be repeated if outside the time window.

Consenting patients will be enrolled into the study once all eligibility has been confirmed. In the patient's interests, best efforts will be made to ensure that consent and screening procedures are performed in the least number of visits possible (sections 5.1.2 and 5.1.3).

5.1.1 Optional Two Stage Screening

Informed consent by patients to screen for the trial may be sought in one process using the main Consent Form (CF) and the main PIS*. Or, optionally, informed consent can be separated into two stages; ultrasound screening followed by, if suitable, informed consent for the full study and remaining screening assessments.

The two stage process allows the ultrasound screening session to be performed and the feasibility of intervention decided in advance of commencing other screening assessments. This can avoid the patient having to complete informed consent for the full study and potentially undergoing other screening tests before ultrasound screening. A separate Ultrasound Screening CF and Patient Information Sheet (PIS) are available for this purpose. Potential participants who consent for ultrasound screening who are successfully screened will then be provided with the main PIS and, after informed consent, be offered to complete consent for the other screening assessments. If eligible the patient can then be enrolled on the study.

**The main PIS and main CF encompasses the ultrasound session screening assessment, therefore it is not necessary to complete the separate ultrasound screening CF and PIS if the one stage process is planned and the patient has provided informed consent on the main CF.*

5.1.2 Informed Consent

Potential participants will be given a current, approved version of the PIS. They will also receive clear verbal information about the study detailing no less than: the nature of the study; the implications and constraints of the protocol; the known side effects and any risks involved in taking part. It will be explained that they will be free to withdraw from the study at any time, for any reason, without prejudice to future care, and with no obligation to give a reason for withdrawal. They will have at least 24 hours to consider the information provided and the opportunity to question the Investigator, their GP or other independent parties before deciding whether to participate. The patient must personally sign and date the latest approved version of the relevant CF before any study procedures are performed, outside of standard care.

The Investigator (or delegate) who obtains consent must be suitably qualified and experienced. All delegates working on behalf of the investigator must be authorised by the PI. The Investigator is responsible for ensuring that the trial consent procedures comply with current applicable GCP Regulatory and ethical requirements. Informed consent discussions and outcomes must be well documented in the medical record. The Investigator (or delegate) must be satisfied that the patient has made an informed decision before taking consent. The patient and the Investigator (or delegate) must personally sign and date the current approved version of the informed consent form in each other's presence. A copy of the information and signed consent form will be given to the participant. The original signed form will be retained in the medical record and a copy is to be filed in the Investigator Site File.

Contraceptive/ Pregnancy counselling
Doxorubicin, the active chemotherapy agent of the study drug (ThermoDox®) can cause foetal harm when administered to a pregnant woman. Animal studies have demonstrated teratogenic and embryotoxic effects at low doses. There are no adequate and well-controlled studies in pregnant women.

All participants must be advised on the need to use reliable methods of contraception during the study and for six months after intervention. The advice should include:

(1) The acceptable methods, including: male or female sterilization, implants, injectables, combined oral contraceptives, some intrauterine devices (IUDs), and abstinence.
(2) The recommendation that a barrier method should be used in addition to another form of contraception.
(3) Males should continue to take these precautions for a minimum six months after the intervention.
(4) Females should continue to take these precautions a minimum of six months after the intervention.
(5) That any pregnancy (also applies to females partners of male trial subjects) occurring within six months of the trial intervention will be followed up and the outcome of all pregnancies (spontaneous miscarriage, elective termination, normal birth or congenital abnormality) will be reported and followed up even if participant is discontinued from the study.

Breast-feeding counselling
There is the potential for serious adverse effects in breast-feeding infants from doxorubicin administered to nursing mothers and consequentially, breast-feeding women are excluded from this study (see exclusion criteria).

5.1.3 Screening Assessments

Inclusion / Exclusion Criteria
Sections 4.1.1 & 4.1.2.

Medical History
Medical History including prior diagnosis, prior oncological disease, prior treatment (including chemotherapy & surgery) and concomitant diseases will be recorded.

Demographics
The date of birth and gender will be recorded.

The WHO performance status (Appendix 1).

Concomitant Medication
These will be recorded in CRFs as per section 9.4.

Physical Examination
An abdominal exam and cardiorespiratory exam will be performed.

Vital Signs
Temperature will be recorded. Resting pulse and blood pressure (BP) measurements will be taken after the participant has been in a sitting position for at least five minutes.

Height & Weight
Body Surface Area (BSA) and Body Mass Index (BMI) will be calculated

Haematology & Biochemistry Tests

- Full Blood Count (FBC); including Haemoglobin (Hb), white blood cells (WBC) with differential count (neutrophils and lymphocytes), and platelets
- Biochemical profile; including sodium, potassium, calcium, phosphate, urea, creatinine
- Liver profile; including total protein, albumin, bilirubin, ALP, AST and/or ALT and LDH
- Group & Save (as standard for surgical candidates, date of valid test to be recorded on CRF, not blood group)
- Clotting Studies; including PT and APPT

Urinary Tests +/- Pregnancy Test

Tests to be performed will be:

- Urinalysis by dipstick.
- In women of childbearing age, a serum or urine Human Chorionic Gonadotropin (HCG) pregnancy test

ECG Test

A 12-lead ECG will be taken for each participant. The eligibility of participants with abnormal AND clinically significant results must be confirmed.

Echocardiogram

An echocardiogram must be performed at baseline to assess LVEF. The test will only be repeated if clinically indicated.

Pre-operative Anaesthetic Assessment (ASA Classification)

A pre-operative assessment will be performed by a suitably trained doctor or nurse, usually in consultant anaesthetist-lead clinic. During this time the previous investigations (as per standard departmental practice) will be reviewed and a decision made if the participant can proceed with a General Anaesthetic. The date and outcome of the assessment (ASA classification) will be recorded.

5.1.3.1 Ultrasound Screening Assessment

A focused diagnostic ultrasound scan will be performed in order to choose suitable tumour area(s), which are easily identified on ultrasound and amenable for biopsy. For participants in both Parts I and II, a single liver tumour amenable to intervention will be targeted. The tumour selected for intervention will be treated with a combination of ThermoDox® and FUS. Refer to section 5.4.3 for more information on the FUS intervention.

As assessed at the Ultrasound session, in order to proceed with the study, the participant must:

1. Have one or more incurable (unresectable, non-ablatable-for cure) liver tumours of size 1cm or greater, which is amenable to ultrasound-guided biopsy
2. Have the total volume to be treated no larger than either removal of 3 hepatic segments or removal of more than 30% of total liver volume (as per maximum surgical limit)
3. Be an appropriate candidate for FUS with consideration given to the following factors:
    a. Overall health of liver
    b. Absence of significant cirrhosis
    c. Absence of significant ascites
    d. Ultrasound path free of previous radiotherapy or scar tissue
    e. Ultrasound path free of overlying skin ulceration

In some circumstances, if a participant is found to be ineligible for Part I of the study due to anatomical tumour location, they may still be eligible for Part II. This is because real-time temperature monitoring is not required in Part II and thus the anatomical position of a given tumour area may be more amenable to delivery of the intervention.

5.2 Evaluations during the intervention (d-1 to d2)

Evaluations on day -1 (pre-Intervention day)

- Dose of oral dexamethasone given as pre-medication (section 5.5.2 & 9.3)
- Baseline scans (Usually on d-1 but may be performed from day -7 to day 1 provided before intervention):
    - Liver MRI/MR -Spectroscopy
    - Liver pCT

- Whole body FDG PET-CT

Evaluations on day 1 (intervention day)

- Confirm d-1 pre-medications were given.
- Day 1 pre-medications given
- Confirmatory portable ultrasound to confirm tumour location before patient anaesthetised
- General anaesthetic start and finish time
- Dose of ThermoDox® administered, start and finish time
- Start and finish time of final FUS exposure and FUS parameters
- Thermometry data (Part I only)
- Bloods samples for pharmacokinetics (Pre-Treatment, Unreleased and Released plasma samples)
- Biopsy samples for assay quantification ± microscopy:
  - Part I: Pre-Treatment, Unreleased, Released
  - Part II: Released
- Adverse event monitoring

Evaluations on day 2 (recovery day)

- Physical exam
- Vital signs: Temperature, resting pulse and blood pressure (BP) after participant has been in a sitting position for at least five minutes
- Full Blood Count (FBC); including Haemoglobin (Hb), white blood cells (WBC) with differential count (neutrophils and lymphocytes), and platelets
- Biochemical profile; including sodium, potassium, calcium, phosphate, urea, creatinine
- Liver profile; including total protein, albumin, bilirubin, ALP, AST and/or ALT and LDH
- Post-treatment Liver MRI/MR-Spectroscopy (within 36 hours, and ideally within 24 hours, of intervention[18])
- Adverse event monitoring

5.3 Follow-up and end of study evaluations (within 60 days)

Follow-up 1: Day 15 (±3 day tolerance)

Parts I & II:

- Full Blood Count (FBC); including Haemoglobin (Hb), white blood cells (WBC) with differential count (neutrophils and lymphocytes), and platelets
- Biochemical profile; including sodium, potassium, calcium, phosphate, urea, creatinine
- Liver profile; including total protein, albumin, bilirubin, ALP, AST and/or ALT and LDH
- Adverse event monitoring

Follow-up 2: Day 30 (+7 not <30)

Parts I & II:

- Adverse event monitoring
- Concomitant medication recording
- Full Blood Count (FBC); including Haemoglobin (Hb), white blood cells (WBC) with differential count (neutrophils and lymphocytes), and platelets
- Biochemical profile; including sodium, potassium, calcium, phosphate, urea, creatinine
- Liver profile; including total protein, albumin, bilirubin, ALP, AST and/or ALT and LDH

Follow-up Scans: From Day 12 - Day 60 post-intervention as defined in the summary of schedule events (one set of scans at 4 weeks follow-up, the other being flexibly timed in accordance with emerging radiological data from previous study participants)

Parts I & II:

- Maximum of two follow-up Liver MRI/MR-Spectroscopy scans
- Maximum of two follow-up Liver pCT scans
- Maximum of two follow-up whole body FDG PET-CT scans

---

[18] The time of intervention is defined as the time at which the final FUS exposure is complete

If a participant is withdrawn from the study following the intervention, adverse event recording should continue for 30 days post-intervention. No other follow-up evaluations will be required.

5.4 Trial Procedures

5.4.1 Preparation for Intervention

Part I and Part II

Day -1 (pre-Intervention):

- The participant attends the day before intervention and is given the first dose of oral premedication within 24 hours of the procedure. If required, the patient may be admitted for the pre-medication and stay in hospital until completion of evaluations on Day 2 (see section 5.2 & 5.3 for evaluations).

Day -1 (pre-Intervention) or Day 1 (Intervention):

- The liver tumour identified for intervention in the Ultrasound Session is correlated with both MRI and CT images and may be re-examined by ultrasound in advance of anaethetising for the intervention.

5.4.2 Intervention

During the intervention the following people are usually present: the Clinical Investigator and/or Co-investigators, anaesthetist, anaesthetic Operating Department Practitioner (ODP), specialist oncology nurse, trained HIFU specialist, Radiologist for biopsy, Radiology nurse and, in Part I, research engineer for real-time data acquisition.

The participant is intubated and anaesthetised. Antibiotic prophylaxis is given on induction. The participant is positioned over the HIFU machine optimally to allow access via the HIFU transducer across the water-bath and access to insert the coaxial needle for taking biopsy or monitoring temperature. At the time of coaxial needle insertion the skin over the puncture area is shaved if necessary, prepared appropriately and a sterile drape is applied.

A detailed Ultrasound Session will have taken place previously (section 5.1.3.1). Further ultrasound planning occurs immediately prior to intervention. This includes three-dimensional tumour evaluation using the diagnostic ultrasound component of the device that will be used to administer FUS exposure. The non-mobile HIFU therapeutic device is located in a purpose-built unit on the Churchill Hospital site and consequently all intervention will take place in this unit.

The HIFU therapeutic device is used to generate hyperthermia in the contoured region of the target tumour using optimised levels of FUS. More information on the FUS procedure can be found in section 5.4.3.

The participants' liver tumours are treated with a combination of FUS and/or ThermoDox® as determined in the Ultrasound Session (section 5.1.3.1). Intervention is performed under General Anaesthetic and the participant is kept in overnight for monitoring following the procedure.

Part I

For each participant in Part I, the optimal FUS exposure parameters to achieve the desired range of mild hyperthermia (and consequent ThermoDox® release) will be determined. The power (watts), duty cycle (or continuous) and duration of exposure will be varied to optimise the temperature response at the tumour site and with a view to achieving targeted doxorubicin delivery.

Day 1 (intervention):

- A co-axial biopsy needle is positioned through the sterile field into the centre of tumour targeted for intervention, under ultrasound guidance.
- A Pre-Treatment Biopsy of the tumour is taken via the co-axial needle, and used as a baseline prior to any intervention, along with the Pre-Treatment blood sample.
- A clinically approved thermometry device is then positioned through the co-axial needle to the target area and is used to obtain real-time thermometry measurements during the FUS exposure.
- The HIFU therapeutic device is aligned to the thermometry device using short test exposures of FUS.

- The tumour area is then exposed to varied and considered FUS parameters (section 5.4.3) until the thermometry confirms that the target range of mild hyperthermia has been achieved within the tumour(*).
- In addition to the FUS exposure parameters (power (watts), duty cycle, duration of treatment) and associated thermometry data, the location of tumour (liver segment) and patient BMI will be recorded for each intervention.
- The thermometry device is then replaced and a cool-down period allows the tumour to return to body temperature.
- Once the tumour has returned to normothermia, the prescribed dose of ThermoDox® is infused over a duration of 30 minutes.
- A second Unreleased Drug Biopsy of the target area is then taken via the coaxial needle (Note: ThermoDox® is circulating at this point, but not yet activated). The corresponding Unreleased Drug blood sample is also taken at this stage for pharmacokinetic analysis within 15 minutes of infusion end.
- The tumour area is then re-exposed to FUS using the exposure parameters identified earlier as being suitable to generate mild hyperthermia within the tumour. During this second FUS exposure, ThermoDox® is circulating between therapeutic and peak levels.
- After completion of the final FUS exposure, a final Released Drug biopsy is taken via the co-axial needle with the corresponding Released Drug blood sample.
- Before removing the coaxial needle, a Gelfoam® (or equivalent) plug embolization of the needle track is applied through the needle to minimise bleeding risk [47].

*Note that with increasing experience, the study team may elect to omit the step of pre-heating the tumour prior to drug delivery (1st FUS exposure) and drug infusion may be commenced shortly after alignment.

The steps of intervention of the liver tumour in Part I are summarised in Figure 4.

The paired Pre-treatment, Unreleased and Released Drug samples (bloods and biopsies) are sent for analysis in an appropriate laboratory. Please refer to section 7 for more information on sample analysis.

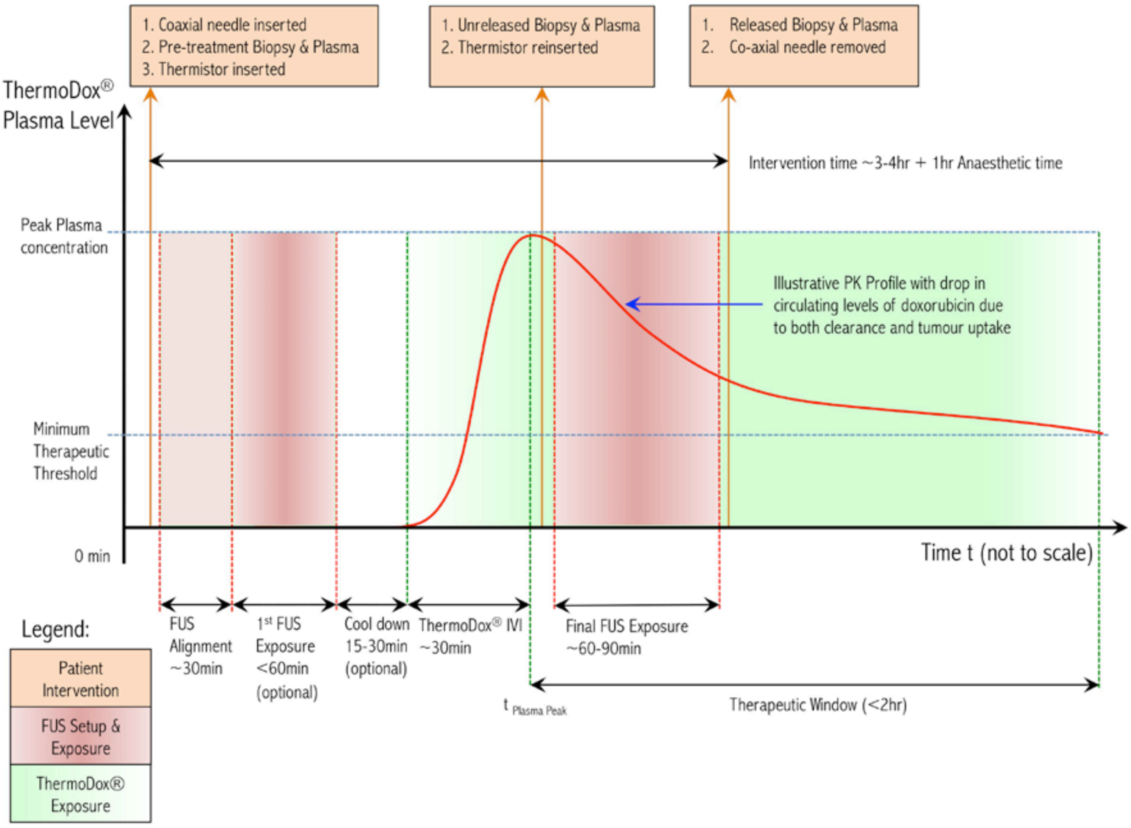

Figure 4: Timeline for combination exposure of the target liver tumour in Part I (not to scale, total plasma doxorubicin PK curve is for illustrative purposes to demonstrate anticipated PK trend and is not based on actual PK data)

Part II

The optimised FUS acoustic parameters for Part I will be used in Part II. The release of doxorubicin and its therapeutic effect will be investigated. The PI or delegate will have already identified a suitable solitary target liver tumour for intervention at the Ultrasound Session (section 5.1.3.1).

Day 1 (Intervention):

- Preparation as for Part I with the exception that no real-time thermometry data acquisition or pre-intervention biopsies are required in Part II, thus the co-axial needle for biopsy can be inserted after the FUS exposure. Three peripheral blood samples for plasma analysis are taken at the corresponding stages of the intervention (Pre-treatment, Unreleased and Released) as for Part I. The time the blood and biopsy samples are taken must be noted.
- ThermoDox® is infused as per prescribed dose for a duration of 30 minutes.
- After the infusion, the lesion identified for intervention (ThermoDox® release by mild hyperthermia) is exposed to the planned FUS exposure dose, as optimised in Part I, taking into consideration the patient BMI and tumour location (section 5.4.3).
- The non-FUS control tumour, if available, is exposed to ThermoDox® alone without FUS.
- As for Part I, the FUS exposure parameters, location of tumour (liver segment) and patient BMI will be recorded for each evaluation.
- The co-axial needle is inserted and up to two ultrasound-guided Released Drug Biopsies of the tumour receiving intervention are taken, whilst the participant is still under General Anaesthetic. A biopsy is not taken of the control tumour exposed to ThermoDox® only. A corresponding Released Drug blood sample is also taken.
- Before removing the coaxial needle, a Gelfoam® (or equivalent) plug embolization of the needle track is applied through the needle to minimise bleeding risk [47].

The steps of intervention for the liver tumour in Part II are summarised in Figure 5.

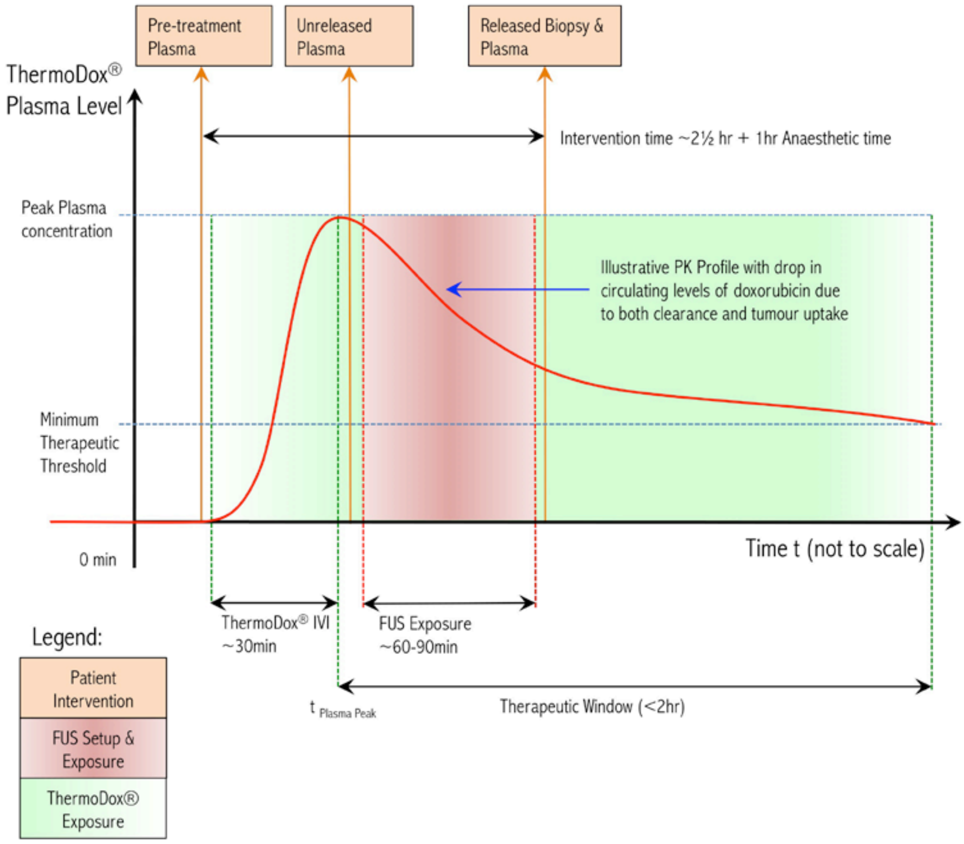

Figure 5: Timeline for combination exposure of the target liver tumour in Part II (not to scale, total plasma doxorubicin PK curve is for illustrative purposes to demonstrate anticipated PK trend and is not based on actual PK data)

5.4.3 Focused Ultrasound Procedure

The focused ultrasound exposure will be performed using the Haifu® Model-JC200 Focused Ultrasound Tumour Therapeutic System at the Churchill Hospital site.

The acoustic window identified in the Ultrasound Session (section 5.1.3.1) is recreated with the participant in position and under anaesthetic. Slice by slice therapy then follows: the tumour volume consists of several two-dimensional cross-sectional 'slices', and the shape and size of the tumour within each slice will naturally vary. In this way, the radiologically accessible tumour volume is divided into sequential slices, at 2-10 mm intervals depending on the therapeutic plan as shown in Figure 6. In therapy mode, the tumour volume is treated with focused ultrasound to attempt to attain regional hyperthermia in the tumour for duration of at least 30 minutes and ideally 60-90 minutes.

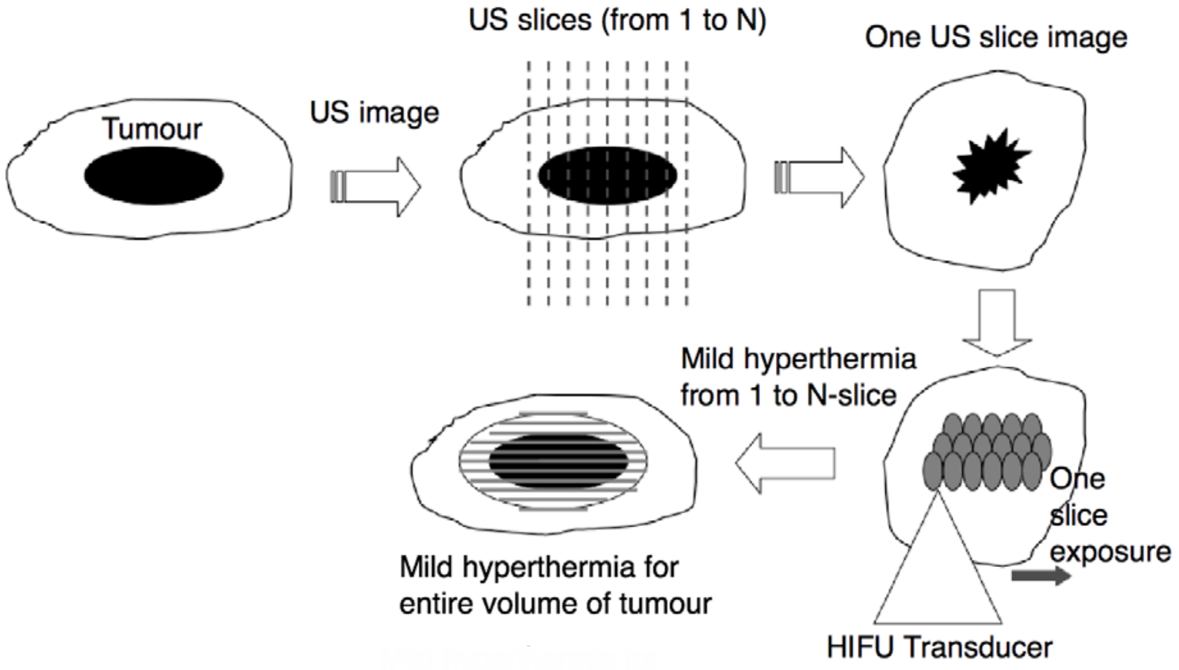

Figure 6: Schematic representation of the row-by-row, slice-by-slice approach used to induce mild hyperthermia into the target tumour volume using a stop/start single-shot mode treatment plan. Moving beam (linear mode) is also under investigation for hyperthermia using the same approach.

5.4.4 Passive Acoustic Monitoring

FUS exposure will be monitored using B-mode diagnostic Ultrasound that is incorporated into the HIFU therapeutic device. During exposure, a prototype Passive Cavitation Detector will optionally be used to simultaneously passively monitor ultrasonic emissions from the targeted tumour area. This prototype has previously been demonstrated during a previous study, to not interfere with FUS exposures using the JC200.

5.4.5 Participant Monitoring

All participants will be monitored continuously while under General Anaesthetic and throughout the intervention. The anaesthetic team (anaesthetist and ODP) will perform this monitoring according to standard protocols for HIFU.

6 EARLY PATIENT WITHDRAWAL

Withdrawal

During the course of the trial, a patient may withdraw early from completing the trial. This may happen for a number of reasons, including:

- Unacceptable toxicity
- AE/SAEs requiring discontinuation
- Clinical decision
- Patient decision

Possible reasons for a clinical decision to remove a participant from the study include, but are not limited to:

- New exclusion criteria including new pregnancy
- Significant protocol deviation or non-compliance with the protocol

Patient withdrawals must be documented in the eCRF. SAEs must be followed until resolution. The reason for withdrawing from treatment early should be clearly documented in the medical records.

Consent Withdrawal

A patient may withdraw consent from study participation (Consent withdrawal) at any time for any reason. Every effort should be made to ascertain the reason for withdrawal and this should be documented in the medical records. The trial office must be informed via completion of the Consent Withdrawal eCRF. Under these conditions, investigators are still responsible to follow up any SAEs till resolution.

For data collection purposes, subjects are considered as completing the study if they have died during the study, are lost to follow-up, or withdraw consent.

6.1 Patient evaluability and replacement

Patients who are not evaluable for the primary endpoint will be replaced.

7 SAMPLES FOR LABORATORY ANALYSIS

7.1 Samples to be analysed in local Trust's laboratories

7.1.1 Diagnostic Laboratories

Samples for haematology, biochemistry and coagulation analysis will be labelled with unique study patient identifiers and sent to the local hospital diagnostic laboratory. Results will be processed in the standard way and entered into the routine hospital reporting system. Samples will be stored, held, reported and subsequently destroyed in accordance with standard local laboratory practice.

7.1.2 Pathology

No pathology samples are required for pathological analysis in the Trust during any part of the study period, although in the evaluation of histological sections of biopsy tissue, the opinon of an NHS pathologist may be sought.

7.2 Samples to be sent to and analysed in a Central Laboratory

The following samples will be analysed at the Oxford Cancer Research Centre:

- Pharmacokinetic blood samples to measure Unreleased and Released plasma levels of doxorubicin, using Pre-Treatment Plasma as the control
- Biopsy samples to quantify intra-tumoural uptake of doxorubicin (three in Part I, up to two in Part II)

Details of sample preparation, labelling and despatch are provided in the TARDOX Sample Handling Manual.

7.3 Pharmacokinetic assays

Plasma samples will be used to quantify the levels of doxorubicin as per the time points in section 5.5.3. The infusion start & stop times, duration of FUS exposure and sample collection times during intervention will be recorded.

7.4 Pharmacodynamic assays

Not applicable to this study.

7.5 Summary of samples/assays to be taken during the study

Assay/sample handling and storage will be managed according to separate written instructions. The following summarises the arrangements for collection, close to patient handling, timings, and analytical laboratories responsible. Research assays will be performed according to separate laboratory SOPs.

7.6 Samples for Biobanking

Participants in this trial will be invited to permit the long term retention of samples left over after protocol-specified analyses for use in possible other future research linked to trial data. Consent to participate in this aspect of the trial is optional and not a requirement of participation in the main study.

7.7 Labelling and confidentiality of samples sent

All samples sent to analytical Laboratories will be labelled with the trial code, trial patient number, and the date & time taken. Should a laboratory receive any samples carrying patient identifiable information the recipient must immediately obliterate this information and re-label. The study site will be informed of their error.

7.8 Clinical reporting of exploratory research assay results

The results of the TARDOX trial research assays are exploratory and are not intended to influence the individual participant's medical care. Findings will not be reported routinely to the responsible clinician except in the unlikely event that the result might be beneficial to the participant's clinical management.

7.9 Trial sample retention at end of study

The Chief Investigator has overall responsibility for custodianship of the trial samples. Laboratories are instructed to retain any surplus samples pending instruction from the Chief Investigator on use, storage or destruction. It is possible that new or alternative assays may be of future scientific interest. At the end of the research study any surplus samples may be retained for use in other projects that have received ethical approval. Hence, any surplus study samples may be transferred to a licensed tissue bank where they will be managed in accordance with applicable host institution policies and the Human Tissue Act requirements.

7.10 Withdrawal of consent for sample collection and/or retention

A patient may withdraw consent to provide samples for research at any time without giving a reason. The Investigator must ensure that their wishes are recorded in the medical record and will inform the trials office accordingly. The investigator should discuss with patients the valuable use of samples that have already been provided and under circumstances where these samples have already been processed and anonymised, it would not be possible to destroy such samples.

8 INVESTIGATIONAL MEDICINAL PRODUCTS (IMPS)

All participants will receive the IMP (ThermoDox® drug) for a liver tumour targeted with mild hyperthermia. Following the required pre-medication of steroids and anti-histamines (Table 5), participants will receive a single therapeutic dose of ThermoDox®.

For information on the packaging, supply, handling, storage and accountability of ThermoDox® please refer to section 11.

8.1 Name of IMPs

ThermoDox®, manufactured under authority of Celsion, USA.

8.2 Treatment dose

50mg/m^2 iv infusion of ThermoDox® in 250ml 5% dextrose over 30 minutes immediately before final FUS exposure.

8.3 Duration of treatment

One day only; participants should receive a single iv infusion of ThermoDox® at a dose of 50mg/m^2 on the day of the intervention in accordance with specific timing as dictated by the team administering the intervention. No further doses are required or should be given as part of this study in either Part I or II. A participant having a dose of ThermoDox® in Part I is not eligible for re-entry into Part II for any further dose.

8.4 Management of drug administration

In order to maximise peak ThermoDox® concentration, FUS exposure of the target tumour area will be initiated at peak therapeutic levels, i.e. soon after the infusion has completed. All FUS exposures started after commencing the ThermoDox® infusion should be completed within 4 hours of the infusion start time. Please refer to section 11.6 for information regarding drug preparation and dosing dispensing.
Steroids are given as a pre-medication (section 9.3) to avoid immediate-type hypersensitivity reactions to liposomes contained within the ThermoDox® product. All participants must be adequately pre-medicated before commencing the ThermoDox® infusion. In the case of hypersensitivity reaction, the participant would be treated according to local hospital policy. In the case of an immediate hypersensitivity reaction manifesting as a localised skin reaction only, the infusion will be immediately stopped until the reaction subsides. Depending on severity and clinical judgement, in some cases the infusion may be restarted.

Extravasation Injury
Administration of ThermoDox® should be closely monitored for local site reactions and at the first sign of extravasation, the infusion must be immediately stopped. Local protocols should be used to treat the affected area.

8.5 Special precautions

Special precautions for doxorubicin, the active agent in ThermoDox®, are detailed in Appendix 1 of the ThermoDox® IB [44], The Doxorubicin Package Insert.

8.6 Actual versus ideal body weight

The dose of ThermoDox® will be calculated for each patient based on actual body weight.

8.7 Dose modification

Dose modification is not applicable to the TARDOX study, which has one single dose exposure.

8.8 Calculating and recalculating BSA/doses

The patient's height and weight at baseline will be used to estimate the body surface area (BSA) using a standard formula such as DuBois or Boyd's (Table 4), which will determine the dose of ThermoDox® to be given during the intervention. Weight will be rechecked the day before intervention (d-1). If a patient's weight increases or decreases by >=10% before intervention, the dose of ThermoDox® should be recalculated.

Height ________________ (cm)		
Weight ________________ (grams)		
Dose Assignment (50mg/m^2) Dose Adjustment __________ mg/m^2		
Equation 1: LOG Weight (grams)		
Equation 2: (0.0188 x LOG Weight (grams))		
Equation 3: (0.7285 - (0.0188 x LOG Weight (grams))		
Equation 4: [Weight (grams)]$^{(0.7285 - (0.0188 \times \text{LOG Weight(grams)})}$		
Equation 5: 0.0003207 x Height (cm)$^{0.3}$		

**BSA (m^2)** = 0.0003207 x Height (cm)$^{0.3}$ x Weight (grams)$^{(0.7285 - (0.0188 \times LOG\ Weight\ (grams))}$		

Table 4: Stepwise equations for calculation of Boyd's formula for calculation of body surface area (BSA)

8.9 Dose capping

No doses are to be capped.

8.10 Dose-banding

No doses are to be dose-banded.

8.11 Compliance

The IMP will be given in a single dose during the study and thus compliance is not applicable.

8.12 Management of overdose

Acute overdose of ThermoDox® and thus doxorubicin may lead to acute cardiac alterations within twenty-four hours and myelosuppression usually 10-15 days following overdose. Urgent medical attention must be sought for which treatment will vary depending on the circumstances, but may involve reverse barrier nursing, intravenous antibiotics, transfusion of blood products, symptomatic treatment of mucositis and cardiology input. More information can be found in Appendix 1 of the ThermoDox® IB [44].

9 OTHER TREATMENTS (NON-IMPS)

9.1 Focused Ultrasound

Focused ultrasound (FUS) by a clinically approved HIFU therapeutic device will be used in this study to deliver acoustic energy capable of inducing mild hyperthermia in the target region.

9.2 Background systemic therapy

Not applicable to the TARDOX study.

9.3 Support medication

Table 5 below details the pre-medications required for ThermoDox®, which start on day minus one, i.e. the day before intervention & the day of admission

Timing and Steps	Premedication Regime
24 hours prior to study drug treatment (day -1)	Subjects take oral premedication; dexamethasone (8-10mg each) every 12 hours x 3 doses; the third dose should be taken the morning of treatment.
Study Intervention day (day 1):<br>Step 1	Confirm that the participant has taken the oral premedication the previous day.
Study Intervention day (day 1):<br>Step 2	Thirty to 60 minutes prior to study drug infusion, participant is administered premedication by IV infusion:<br>a) dexamethasone 20 mg IV<br>b) chlorpheniramine 10 mg IV<br>c) ranitidine 50 mg IV

Table 5: Premedication and Study Treatment Infusion Instructions for ThermoDox®

9.4 Concomitant medication and non-drug therapies

Concomitant medication may be given as medically indicated. All patients will be asked to provide a complete list of prescription and over-the-counter medications, vitamins, and/or herbal supplements that have been taken within the previous 4 weeks prior to screening. They must also inform the Investigator about any new medication started while in the trial. Details (including indication, doses, frequency and start / stop dates) of concomitant medication taken during the trial until the completion of the off-study visit must be recorded in the medical record and the appropriate CRF.

Investigators should be aware of potential drug-drug interactions particularly inhibitors of the Cytochrome P450 (CYP3A4) drug metabolizing enzyme system. Thus when co-administering ThermoDox® with drugs classified as 'substrates', there is a potential for ThermoDox® to produce higher plasma concentrations than normal of the 'substrate' drug. Conversely, when co-administering ThermoDox® with drugs classified as 'inhibitors', there is a potential for the 'inhibitor' to produce higher plasma concentrations of ThermoDox® than normal. In general, any drug's toxicity correlates with its plasma concentration, particularly for IV administered cytotoxic drugs. Please refer to sections 9.5 and 9.6 for a list of prohibited and interacting medications.

9.5 Prohibited therapies

Patients should not be prescribed any other anti-cancer or investigational therapies while participating in this study.

9.6 (Potential) Drug Interactions

Investigators should also be aware of potential drug-drug interactions.

Doxorubicin is extensively metabolized by the liver. Changes in hepatic function induced by concomitant therapies may affect doxorubicin metabolism, pharmacokinetics, therapeutic efficacy, and/or toxicity.

10 RADIOTHERAPY

In the ThermoDox® study FUS will be used to induce mild hyperthermia in the target region (section 5.4.3). There will be no radiotherapy per se and no ablative radiotherapy will be performed.

11 DRUG MANAGEMENT

11.1 Drug supplies

ThermoDox® will be provided as commercial stock by Celsion free of charge. It will be shipped from the USA to the UK in a frozen form. It will be manufactured according to the specifications of an existing IMPD (Investigational Medicinal Product Dossier) under strict GMP conditions. A Qualified Person (QP) will release the IMP before sending to the study site. ThermoDox® will be supplied in 20cc vials each containing 30mg doxorubicin in 15ml of solution for intravenous infusion. 30mg vials will come in a carton containing 4 vials. Giving sets, infusion bags, other equipment and all supportive medication is to be sourced and funded locally.

It will be the responsibility of the investigator for ensuring that the study drug is stored at the site pharmacy in accordance with the instructions on the labels. At study initiation, and as needed thereafter, ThermoDox® will be shipped to a responsible person (e.g. pharmacist) at the Investigator's institution, who will check the amount and the condition of the drug and enter these data onto the Drug Accountability Log. Initial supply and re-supply of ThermoDox® will be managed by the trial office. The ThermoDox® accountability logs will be kept and will contain dates and quantities received from Celsion, lot numbers (batch numbers), date and quantity of drug dispensed, dose preparation records, etc. ThermoDox® expiry dates will be routinely checked.

Drugs will be prescribed, tracked, and dispensed according to the local policy for investigator-initiated studies. Only qualified personnel who are familiar with procedures that minimise undue exposure to themselves and to the environment should undertake the preparation, handling and safe disposal of the trial medications.

11.2 Drug ordering

Initial supplies of ThermoDox® are sent out by Celsion after they have been informed by the trial office that all approvals are in place. If required, re-supply of ThermoDox® will be managed by the trial office who will monitor stocks and reorder where required. If a vial of ThermoDox® is accidentally destroyed, e.g. by dropping the vial, accidentally thawing, or through contamination, the pharmacist should contact the trials office for replacement patient supplies.

11.3 IMP Receipt

Acknowledgement of receipt of ThermoDox® supplies will be logged.

Damaged supplies should be destroyed on site and a drug destruction form completed.

11.4 Handling and storage

All investigational study medication will be stored frozen at below -60°C in a secure area with access limited to the Investigator and authorized site staff. All investigational study medication will be administered according to the procedures described herein and in the pharmacy manual. Only participants enrolled in the study may receive the investigational study medication (ThermoDox®), in accordance with all applicable regulatory requirements.

Prior to administration, an appropriate number of frozen ThermoDox® vials must be carefully thawed at room temperature for a minimum of 1 hour. ThermoDox® vials MUST NOT be heated to speed up the thawing process. After removing from the freezer and prior to dilution, the vials are stable for no more than 4 hours at room temperature (15°C to 30°C).

The required amount of ThermoDox® is then drawn into a 50 or 60 ml syringe and is diluted to 250 cc 5% dextrose (D5W). Once reconstituted, it is stable at room temperature for no more than 8 hours. The total volume is infused over a 30 minute period through a free flowing IV line. ThermoDox® should be infused at room temperature. ThermoDox® solutions must not be heated above room temperature during administration.

For detailed storage and preparation instructions, refer to the pharmacy manual that is provided to the pharmacist.

11.5 Labelling

The responsible Pharmacy will ensure that IMP supplies dispensed for trial use are appropriately labelled in accordance with all applicable regulatory requirements.

As provided from Celsion, the manufacturer, each carton label will include a minimum of the following specific identifiers that will uniquely identify the product to the production batch used for this study:

- Name of Product (ThermoDox®)
- Name of the study Sponsor
- Sponsor Protocol Number
- EudraCT Number
- Lot number
- Manufacture Date
- Retest Date
- Chief Investigator

Each vial label will include a minimum of the following specific identifiers that will uniquely identify the product to the production batch used for this study:

- Name of Product (ThermoDox®)
- Name of the study Sponsor
- Sponsor Protocol Number

The ThermoDox® product is provided in a frozen preparation containing 30mg of doxorubicin in 15ml of solution, within a 20cc amber vial. A typical patient will require the use of 4 vials or less, for a 50 mg/m^2 dose. The product is provided in four vial cartons, so typically one carton per patient is required.

11.6 Dosing dispensing

ThermoDox® requires dilution in a 5% dextrose solution in water (D5W) before administration.

11.7 Drug accountability

Celsion will supply the study medication to Churchill Hospital pharmacy site via their approved manufacturer and usage will be logged. Any unused medication will be destroyed at the end of the study or returned to the manufacturer. All movements of study medication between Celsion and pharmacy will be documented.

Full drug accountability logs must be maintained for ThermoDox® using the Logs provided. Hospitals may amend the Drug accountability Logs provided or use their own documentation if prior approved with the trial office.

The drug dispensing and inventory logs should be kept up to date and have to contain the following information; patient identifier, date and quantity received at site, date and quantity dispensed, date and quantity returned/destroyed at site. The inventory must be available for inspection by the monitor at every monitoring visit.

At the conclusion of the study the overall numbers of drug shipped to the centre, the number dispensed and the number destroyed will be provided by the pharmacy. An account must be given of any discrepancy.

11.8 Drug returns from patients

Not applicable.

11.9 Drug destruction

ThermoDox® is the IMP for the TARDOX study. Once it has been defrosted for use it must never be refrozen for reuse. Therefore any thawed ThermoDox® must be disposed of according to local hospital policy. Please refer to Table 6 below, which details when drug should be disposed of.

Used / partially used vials	Disposal at site according to local hospital policy.
Drug left unused / expired drug	Disposal at site according to local hospital policy. A dated certificate of disposal should be completed and retained in the Pharmacy File.
Drug left unused	At the end of the study, once authorised to do so, any unused drug should be disposal of at site according to local hospital policy. A dated certificate of disposal should be completed. The original should be placed in the Pharmacy File and a copy faxed to the Trials Unit.
Patient returns	N/A

Table 6: Disposal information for the ThermoDox® IMP used in the TARDOX study.

11.10 Occupational safety

As per IB and site procedure.

12 EVALUATION OF RESPONSE

Response to intervention will be assessed both in terms of intra-tumoural uptake of doxorubicin following intervention on the same day and the radiological response of the target tumour during the follow-up stage of the study. The response to intervention for each participant will be assessed using a combination of the collected data in both Part I and Part II:

1. Biopsy analysis
2. Blood results

3. Follow-up scans (Liver MRI/MR-Spectroscopy, Liver pCT and whole body FDG PET-CT)

Biopsy analysis
Statistical analysis will be performed to establish if there is significant improvement in dose recovery between the Unreleased Drug Biopsy and the Released Drug Biopsy.

Follow-up Imaging
Although not essential for the success of this study, whose primary objective is to demonstrate targeted release of doxorubicin, response to intervention is a tertiary (exploratory) objective in Part II (section 12.1).

12.1 Measurement of disease for solid tumour

Objective disease response in the target tumour and the non-FUS control tumours, if any, will be independently measured according to the principles of CHOI and RECIST response evaluation using MRI and CT [11, 12] and SUV_{max} using PET-CT [13]. In both Part I and Part II, radiological tumour response with CT and MRI scans will be evaluated prior to study entry and following intervention as per the follow-up schedule (section 2.3). Response classification will be used in the analysis of trial data.

12.2 Tumour assessment

A clinical and liver-focused radiological evaluation of malignancy, as judged appropriate by the Investigator, and in line with the protocol, must be performed before starting the study treatment where applicable. In addition to ultrasound examination for screening purposes (section 5.1.3.1), the same methods that detect liver tumours at baseline(Liver MRI/MR-Spectroscopy, Liver pCT and whole body FDG PET-CT) will be used to follow these tumours post-intervention (section 5.3). To ensure compatibility, the radiological assessments used to assess response must be performed using identical techniques. Imaging based evaluation is preferred to evaluation by clinical examination when both methods have been used to assess the anti-tumour effect of a treatment.

12.2.1 Baseline evaluations

These will include radiological measurements of the FUS-targeted tumour and, where available, suitable control liver tumour(s) by Liver MRI/MR-Spectroscopy, Liver pCT and whole body FDG PET-CT. Only these selected liver tumours will be evaluated and followed-up for response. Control tumours will be selected based on their measurability at baseline and ideally be size-matched to the target tumour where possible. In some cases there may be no appropriate control tumours, in this case the only the target tumour will be evaluated.

12.2.2 Evaluations during treatment and at off-study

Tumour assessment will be repeated as per the schedule of events and measured in accordance with the principles of CHOI and RECIST [11, 12].

13 ASSESSMENT OF SAFETY

Adverse event monitoring starts at intervention (Day 1) until Day 30 follow-up visit (which may be scheduled 30 days +7 but not <30 post intervention).. The Investigator will monitor each patient for clinical and laboratory evidence of adverse events on a routine basis throughout the study. Should an Investigator become aware of any study drug or study procedure related SAEs following this period these must also be reported as stated below. All reportable AEs will be followed to a satisfactory conclusion. Any reportable AEs that are unresolved at the patient's last visit in the study are followed up by the Investigator for as long as medically indicated, but without further recording in the CRF.

All AEs reported to the trial office will be processed according to internal SOPs, this includes the reporting procedure and assessment of causality and expectedness for SAEs. The TARDOX trial office may request additional information for any AE as judged necessary.

13.1 Adverse Event Definitions

An Adverse Event or experience (AE) is any untoward medical occurrence in a study subject temporally associated with the administration of an investigational medicinal product (IMP) or a comparator product, whether or not considered related to the IMP or a comparator product. An AE can therefore be any unfavourable and unintended sign, symptom, disease (new or exacerbated) and /or significant abnormal laboratory or physiological observation

temporally associated with the use of a medicinal product. For marketed medicinal products, this also includes failure to produce expected benefits (i.e. lack of efficacy), abuse or misuse. **A Serious Adverse Event (SAE)** is any AE, regardless of dose, causality or expectedness, that:

• **Results in death**	
• **Is life-threatening**	This refers to an event in which the subject was at risk of death at the time of the event. It does not refer to an event, which hypothetically might have caused death, if it were more severe.
• **Requires in-patient hospitalisation or prolongs existing inpatient hospitalisation**	In general, hospitalisation signifies that the subject has been admitted (usually involving at least an overnight stay) at the hospital or emergency ward for observation and/or treatment that would not have been appropriate in the physician's office or out-patient setting. Complications that occur during hospitalisation are AEs. If a complication prolongs hospitalisation or fulfils any other serious criteria, the event is serious. When in doubt as to whether hospitalisation occurred or was necessary, the AE should be considered serious.
• **Results in persistent or significant incapacity or disability**	This means a substantial disruption of a person's ability to conduct normal life functions. It does not include experiences of relatively minor medical significance or accidental trauma (e.g. sprained ankle), which do not constitute a substantial disruption.
• **Is a congenital anomaly or birth defect**	
• **Is any other medically other medically important event**	Defined as an event that may jeopardise the patient or may require intervention to prevent one of the outcomes listed above. Any new primary cancer must be reported as an SAE.

This includes any newly occurring event, or a previous condition that has increased in severity or frequency since the administration of study drug.

An Adverse Drug Reaction (ADR) A response to a medicinal product, which is noxious and unintended.
Response in this context means that a causal relationship between a medicinal product and an adverse event is at least a reasonable possibility.

An Unexpected Drug Reaction is an adverse drug reaction, the nature or severity of which, is not consistent with applicable reference safety information section (referring to information in SPC or IB).

A Suspected Unexpected Serious Adverse Drug Reaction (SUSAR) is a serious adverse drug reaction, the nature or severity of which is not consistent with the applicable product information (e.g. Investigator's Brochure for an unapproved investigational product or SPC for an approved product).

13.2 Clinical laboratory abnormalities and other abnormal assessments as AEs and SAEs

Abnormal laboratory findings (e.g., clinical chemistry, haematology, urinalysis) or other abnormal assessments (e.g., ECGs, X-rays and scans) that are judged by the investigator as clinically significant will be recorded as AEs or SAEs if they meet the definitions given above.
The investigator will exercise his or her medical and scientific judgment in deciding whether an abnormal laboratory finding or other abnormal assessment is clinically significant. By definition, **all Grade 4 laboratory abnormalities should be reported as SAEs**.

13.3 Determining adverse event causality

The Investigator will assess and classify the relationship of an AE to the trial IMP (ThermoDox®) as follows:

Classification	Relationship	Definition
**Drug-related**	**Definitely related**	• Starts within a time related to the study drug administration *and*<br>• No obvious alternative medical explanation.
	**Probably related**	• Starts within a time related to the study drug administration *and*<br>• Cannot be reasonably explained by known characteristics of the patient's clinical state.
	**Possibly related**	• Starts within a time related to the study drug administration *and*<br>• A causal relationship between the study drug and the adverse event is at least a reasonable possibility.
**Not drug related**	**Probably not related**	• The time association or the patient's clinical state is such that the study drug is not likely to have had an association with the observed effect.
	**Definitely not related**	• The AE is definitely not associated with the study drug administered.

The Investigator must endeavour to obtain sufficient information to confirm the causality of the adverse event (i.e. relation to the study drug, focused ultrasound exposure, liver biopsy, other illness, progressive malignancy or other cause) and give their opinion of the causal relationship between each AE and study drug, FUS and/or biopsy procedure. This may require instituting supplementary investigations of significant AEs based on their clinical judgement of the likely causative factors and/or include seeking a further specialist opinion.

13.4 Expected adverse events

Appendix 2 of the current approved ThermoDox® IB [44] lists all the adverse events associated with the use of ThermoDox® in clinical studies. ThermoDox® contains Adriamycin® (doxorubicin) as its active chemotherapy agent and ThermoDox® has a similar toxicity profile to doxorubicin. Appendix 1 of the current approved ThermoDox® IB [44] lists all the adverse events known to be associated with the use of doxorubicin. A copy of this document must be held in the Site File for reference.

Please note much of the adverse event data for ThermoDox® has been obtained from a global, randomized Phase III study (HEAT) which involves combining ThermoDox® with RFA in the treatment of hepatic tumours, in patients with inoperable hepatocellular carcinoma (HCC). Adding 50mg/m^2 of ThermoDox® to RFA was found to be safe with an adverse event profile similar to that of the single agent doxorubicin. However given the difference in patient population, it is possible that in this study population other side effects may occur, or the patient might suffer a more severe reaction.

As per ThermoDox® IB amendment version date 11th October 2013 (the current approved ThermoDox® IB at the time of the initial trial approval), grade 3+ neutropenia and leucopenia were the only[19] SAEs occurring in over 5% of HEAT Study patients and were not seen in the arm receiving RFA without ThermoDox® (Table 7). The detailed and current list of SAEs and AEs in 701 patients for this study, can be found in Appendix 2 of the current approved ThermoDox® IB

[19] The only other grade 3+ adverse events affecting at least 5% of subjects were elevations in liver enzymes which is an expected occurrence in the patient group used for the HEAT study (hepatocellular carcinoma). The RFA alone vs RFA+ThermoDox® study arms did not differ significantly in this respect.

[44]. Further details regarding the frequency of AEs and SAEs in patients treated with ThermoDox® using targeted application of heat by RFA or microwave have been included in a recent publication [10].

**Adverse Event**	**RFA Alone N=347**		**RFA+ThermoDox® N=354**		**p-value**
	N	%	N	%	
Neutropenia	0	0	65	18.9	<0.0001
Leukopenia	0	0.0	21	6.1	<0.0001

Table 7: SAEs affecting at least 5% of patients enrolled in the Phase III, randomized, double-blinded, dummy-controlled study of the efficacy and safety of ThermoDox® in combination with RFA compared to RFA-alone in the treatment of non-resectable HCC. Data obtained from the ThermoDox® Investigator's Brochure (Amendment Version October 11, 2013).

This study involves the use of FUS to induce mild hyperthermia. A recent study performed at Oxford involving 30 patients in four trial protocols has shown *ablative* FUS or high-intensity FUS (HIFU) to be both safe and feasible for the treatment of liver and kidney tumours [45]. Adverse events are summarised in Table 8. It should be noted that thresholds of acoustic power required for mild hyperthermia, as required this study, are envisaged to be well below those required for ablative HIFU and thermal cell death.

	**CTC grade**			
**Event**	**0**	**1**	**2**	**3**
Discomfort at treatment site	6	16	7	1
Skin toxicity at treatment site	22	7	1	0
Oedema at treatment site	22	3	3	2
Fever	26	3	1	0
Other	27	3	0	0

HIFU = high-intensity focused ultrasound; CTC = common toxicity criteria. [a]Number of patients who experienced each adverse event by grade.

Table 8: Adverse events possibly or probably related to HIFU treatment[a]. Reproduced from Illing et al., 2005 [45].

Evidence for the primary endpoint for this study requires laboratory analysis of tumour tissue obtained by image guided liver biopsy through a co-axial biopsy needle as per current guidelines. The complications of performing image guided liver biopsy using guidelines have been reviewed following a recent UK-wide audit [48] and are summarised in Table 9. Out of 3496 cases, 376 patients had minor complication alone, 22 patients had at least one major complication and there were 4 deaths, all attributable to post-biopsy haemorrhage.

A: Minor Complications

Response	Minor Pain	Severe Pain	Hypotension
Yes	338 (9.94)	36 (1.06)	26 (0.75)
No	3062 (90.06)	3365 (98.94)	3426 (99.25)
Total	3400	3401	3452

B: Major Complications

Response	Hemorrhage	Hemobilia	Punctured Kidney	Punctured Bowel	Punctured Gallbladder	Punctured Lung	Puncture of Other Organ	Death
Yes	14 (0.40)	2 (0.06)	1 (0.03)	1 (0.03)	0 (0.00)	1 (0.03)	0 (0.00)	4 (0.11)
No	3462 (99.60)	3453 (99.94)	3457 (99.9)	3457 (99.97)	3455 (100.00)	3454 (99.97)	3471 (100.00)	3482 (99.89)
Total	3476	3455	3458	3458	3455	3455	3471	3486

C: Postbiopsy Procedure

Response	Blood Transfusion	Percutaneous Drainage	Laparotomy	Embolization
Yes	12 (0.34)	4 (0.12)	1 (0.03)	2 (0.06)
No	3470 (99.66)	3464 (99.88)	3467 (99.97)	3469 (99.94)
Total	3482	3468	3468	3471

Table 9: Post- liver biopsy complications, mortality and subsequent treatment and interventions. Data is represented as numbers of cases. Numbers in parentheses are percentages. Reproduced from Howlett et al., 2013 [48].

13.5 Suspected Unexpected Serious Adverse Drug Reactions (SUSARs)

All SUSARS must be reported to the responsible Authority and main REC within 7 or 15 days, depending on the seriousness criteria, by the Sponsor or their delegate within the required timelines. The Sponsor or their delegate will ensure that all SUSARs are also reported to the MHRA. It is therefore important that the SAE form is faxed as detailed below as soon as, and no later than, 24 hours after the site are aware of an SAE or significant updated information. In addition, other safety issues qualify for expedited reporting where they might materially alter the current risk assessment of an IMP or be sufficient to change IMP administration or the overall conduct of the trial. Responsibility for expedited safety reporting will be assigned by the Sponsor.

The TARDOX trial office shall notify Celsion of all SUSAR’s within one working day of becoming aware of them.

13.6 Expedited reporting of SAEs

The following SAE reporting requirements apply regardless of the Investigator’s assessment of the causality or expectedness of the SAE (see section 13.10 for exceptions). All SAEs should be reported on the trial SAE report form (see SAE report form and instructions for completion). Please note that the SAE report form is a paper form and these data cannot be entered into the eCRF system (OpenClinica).

SAE forms must be completed and faxed to the trial office on:

TARDOX Trial Office SAE Fax: 01865 227038

Or emailed (email preferred) to:

TARDOX Trial Office SAE Email: octo-safety@oncology.ox.ac.uk

SAE forms must be completed with all critical elements as soon as possible and submitted within 24 hours of becoming aware of the event all the vital elements being completed. If the SAE has not been reported within the specified timeframe, a reason for lateness must be provided when sending the SAE Report Form.

Investigators should also adhere to their local Trust policy for incident and SAE reporting in research.

The TARDOX trial office shall notify Celsion with a line listing report of all Serious Adverse Reactions (SARs) on a monthly basis.

13.7 Follow-up of Serious Adverse Events

If new or amended information on a reported SAE becomes available, the Investigator should report this on a new SAE form or update the existing form, without obscuring any initial information. All new data must be initialled and dated so that all changes are clearly identified.

Follow up will continue until all the necessary safety data for the event has been gathered. Any SAE that is on-going when a subject completes his/her participation in the trial must be followed until any of the following occurs:

- The event resolves or stabilizes;
- The event returns to baseline condition or value (if a baseline value is available);
- The event is attributed to other agent(s) or to factors unrelated to study conduct.

SAEs that are considered to be definitely unrelated to the trial intervention will be followed up as per related SAEs.

13.8 Adverse events that should be notified to trial office

AEs that are serious must be reported to trial office up to and including 30 days after the intervention date. Any SAE that occurs at any time after completion of ThermoDox® treatment or after the designated follow-up period that the investigator considers to be related to the study drug must be reported

13.9 Reporting Adverse Events on the CRF

All AEs, including Serious AEs must be recorded on the case report forms (CRF) for that patient. The information provided will include date of onset, event diagnosis (if known) or sign/symptom, severity, time course, duration and outcome and relationship of the AE to study drug. Any concomitant medications or other any therapy used to treat the event must be listed. The Investigator will provide an "other" cause for serious AEs considered to be unrelated to the study drug. The study site should ensure data entered into the CRF is consistent with the SAE report information where applicable. SAE forms should be sent to the trials office within 24 hours of the incident occurring.

Each separate AE episode must be recorded. For example, if an AE resolves completely or resolves to baseline and then recurs or worsens again, this must be recorded as a separate AE. For AEs to be considered intermittent, the events must be of similar nature and severity.

<u>Serious Events reporting requirements</u>
Subjects having adverse events will be monitored with relevant clinical assessments and laboratory tests as determined by the Investigator. All adverse events must be followed to satisfactory resolution or stabilization of the event(s) including the investigation for the likely causative factor. Any actions taken and follow-up results must be recorded in the subject's source documentation. Follow-up laboratory results should be filed with the subject's source documentation. For all adverse events which require the subject to be discontinued from the study, relevant clinical assessments and laboratory tests will be repeated, on at least a weekly basis, until final resolution or stabilisation of the event(s). All SAEs will be included in analysis and must be reported to Celsion, regardless of cause, and will be classified as per section 13.3.

<u>Non Serious Events reporting requirements</u>
All Adverse Events (AE) including Adverse Drug Reactions (ADR) occurring up to 30 days following the intervention must be recorded on the CRF. The investigator will decide if those events are related to the medicinal product (i.e. unrelated, unlikely, possible, probable, definitely and not assessable) and the decision will be recorded on the CRF. AE definitely not drug related (i.e. reported as unrelated) will not be considered as adverse drug reactions in toxicity analyses, but reported separately.

<u>Terms and Grading of Events</u>
MedDRA dictionary should be used to code SAES
The NCI CTCAE Version 4.0 (currently up to Version 4.03) should be used to grade AEs and where possible use the Lowest Level Terms provided. Where indicated on the form, provide the severity grade for each AE, and the worst grade recorded.

13.10 Events exempt from being reported as AE/ SAEs

13.10.1 Progression of underlying disease

In this Phase I study, which can only target a single tumour for dosing, progression is a likely outcome in patients with disseminated disease. During the follow-up period, target and control tumours in the liver will be monitored for

progression and these data will be captured on the CRF. Progression of disease by clinical means or of other tumours by radiological means will not be recorded on CRFs, however resultant death during the study period will be captured on the CRF (section 13.10.2). Adverse events including hospitalisation that are clearly consistent with disease progression will not be reported as individual AE/SAEs. Clinical symptoms of progression will only be reported as adverse events if the symptom cannot be determined as exclusively due to the progression of the underlying malignancy, or does not fit the expected pattern of progression for the disease under study. Where disease progression is so evident, in the best interests of the patient, the investigator may elect not to perform further disease assessments.

13.10.2 Death on study

Death due to disease under study is to be recorded on the Notification of Death CRF, and does not require reporting as a SAE, providing the death is not unexpected nor a causal relationship to the IMP or any other trial procedure suspected. Death due to all other reasons must be reported as a SAE.

13.10.3 Elective admissions and supportive care

Elective admissions to hospital for patient convenience or for planned procedures or investigations or treatment as specified in this protocol and standard supportive care are not SAEs, and do not require SAE reporting. Hospital admission for the following supportive care procedures are considered standard for this patient group and should not be reported as SAEs:

- Insertion of stent
- Insertion of Port-A-Cath, PICC line, etc.
- Admission for transfusion

13.11 Development Safety Update Reports

DSURs will be co-ordinated by OCTO and prepared in collaboration with the CI and submitted in parallel to the regulatory authority and responsible Research Ethics Committee within 60 days of the anniversary of the date of regulatory approval. A copy of each report will be provided to Celsion and the sponsor

13.12 Informing Investigators of new safety information

The trial office or the Chief Investigator will ensure that all investigators are kept informed in a timely manner, as new safety profile information becomes available. Investigators are responsible for briefing their study team and onward transmission to R&D office as appropriate.

14 PREGNANCY

Pregnancies (in a participant or partner) occurring whilst taking part in the trial or within 6 months of the intervention require expedited reporting.

A pregnancy form should be completed and faxed to the trial office within the same timelines as an SAE. All reported pregnancies should be followed and the outcome reported using the same form. If the outcome of the pregnancy meets any of the criteria for seriousness, it must also be reported as an SAE. Examples of pregnancy outcomes that are SAEs include reports of:
• congenital anomalies or developmental delay, in the foetus or the child.
• foetal death and spontaneous abortion.
• suspected adverse reactions in the neonate that are classified as serious

Women who become pregnant should be withdrawn from trial treatment immediately

The Trial Office will report all pregnancies and any pregnancy–related SAEs to Celsion.

15 DEFINING THE END OF TRIAL

For this study the end of the trial is defined as:

The last follow-up with the last patient

The sponsor and the Chief Investigator reserve the right to terminate the study earlier at any time. In terminating the study, they must ensure that adequate consideration is given to the protection of the participants' best interests.

16 STATISTICAL CONSIDERATIONS

16.1 Sample size and power

Evaluable participants will be recruited to either Part I or Part II of the study, up to a maximum of 28 evaluable participants overall. Non-evaluable participants will be replaced (section 6.1).

17 STATISTICAL ANALYSIS PLAN

A statistical analysis plan written adhering to the current OCTRU standard operating procedures will be signed off for this trial prior to analysis of primary or secondary aims.

17.1 Inclusion in analysis

All evaluable participants in both Part I and Part II will be included in the final analyses. All patients enrolled in the study, will be accounted for and included in the analyses. The number of patients who were not evaluable, who died or withdrew before treatment began will be recorded.

Variables will be analysed to determine whether the criteria for the study conduct are met. This will include a description of patients who did not meet all the eligibility criteria, an assessment of protocol violations and other data that impact on the general conduct of the study.

Baseline characteristics will be summarised for all enrolled patients. Patients who died or withdrew before treatment started or do not complete the required safety observations will be described and evaluated separately.

Treatment related toxicity will be tabulated by type, grade of toxicity, relatedness to trial treatment and the worst grade toxcitiy experienced

Evaluable for toxicity: All patients will be evaluable for toxicity from the time of their intervention using NCI CTCAE v 4.0 scoring.

Adverse events will be summarised by the number of patients experiencing each type of event. The grades and causality will be reported.

17.2 Subgroup analysis

No subgroup analyses are planned for the study.

17.3 Interim Analyses

As per the study design (section 2) the TMG will review thermometry data to ascertain the point at which Part II of the study will be opened (section 18.2). Following this, there will be an interim analysis of the Part I only secondary objective, which concerns optimal FUS exposure parameters. This analysis and all other analysis will be completed at the end of the study, i.e. once recruitment is closed in Part I and Part II. Once Part II is opened, Part I of the study will also remain open and can be recruited to in parallel. During the screening process the study clinicians will consider patients for the most appropriate Part based on the availability of Part II, the tumour locations, and the present level of experience with FUS hyperthermia for tumours at similar anatomical depths and locations.

17.4 Procedures for reporting any deviation(s) from the original statistical plan

Any deviations / violations must be reported to the TARDOX Trial Office according to the procedure outlined during site initiation. These will be noted in the statistical report.

17.5 Final analysis

Details of the final analysis are described in the Statistical Analysis Plan.

Based upon projected accrual rates, this trial is expected to complete recruitment within 35 months of opening to recruitment. Final analysis will be after all Part I and Part II participants have been followed up for at least one month at the following projected time points:

- Endpoint analysis - Last Patient Last Visit.

18 TRIAL COMMITTEES

18.1 Trial Management Group (TMG)

The Chief Investigator will chair a TMG responsible for overseeing the successful conduct and publication of the trial. The TMG will provide progress reports to the Independent Early Phase Research Trial Steering Committee (IEPTOC) as required. The TMG will make the decision to open Part II of the TARDOX trial. The TMG will also review cumulative reports of all AEs & SAEs. TMG meetings will be scheduled as often as required. Meetings may be by teleconference.

18.2 Data and Safety Monitoring

There is no Data and Safety Monitoring Committee (DSMC). SAEs upon receipt are reviewed by an independent nominated clinician as part of the trial office SAE Standard Operating Procedure. Cumulative reports of all AEs & SAEs are reviewed by the TMG and an Independent Trial Steering Committee (ITSC) will be in place to monitor the safety of the trial.

18.3 Trial Steering Committee

Independent oversight for this trial will be provided by the Independent Early Phase Trial Oversight Committee (IEPTOC). As per the IEPTOC Charter, the majority of members of IEPTOC, including the chair, are independent of the trial. The role of IEPTOC is to provide oversight for the trials to protect the integrity of the trials. It also provides advice through its independent Chairman to the Trial Management Group (TMG), Sponsor, Funder and OCTRU on all aspects of the trial. IEPTOC will meet at least yearly. Any major trial issue may be dealt with between meetings, by phone or by email.

DATA MANAGEMENT

18.4 Database considerations

Data management will be performed via a web-based, bespoke trial database (OpenClinica). OpenClinica is a dedicated and validated clinical trials database designed for electronic data capture. See: http://www.openclinica.org.

The Chief Investigator will act as Data Custodian for the trial. A guide explaining how to use OpenClinica will be provided to every site. The trial office will provide the site with instructions and a video link for training purposes. Relevant trial office staff will have overview of all entered data.

18.5 Case reports forms (CRFs)

The Investigator and study site staff will ensure that data collected on each subject is recorded in the CRF as accurately and completely as possible. The CRFs will not contain any source data. All appropriate laboratory data, summary reports and Investigator observations will be transcribed into the CRFs from the relevant medical record(s). Please ensure that:

- the relevant CRFs are completed.
- all CRF data are verifiable in the source documentation or the discrepancies must be explained.
- CRF sections are completed in a timely fashion, as close to the visit or event being recorded as possible.
- data queries are resolved and documented by authorised study staff, giving a reason for the change or correction where appropriate.

The above considerations also apply to patients who are withdrawn early. If a patient withdraws from the study, the reason must be noted on the appropriate form and the patient must be followed-up as per protocol.

18.6 Accounting for missing, unused, or spurious data.

Missing data found will be chased up and supplemented where possible after consultation with the investigator. The control of the correctness of the data is performed with ranking tests, validity tests and consistency checks. Unused data will be retained as for used data.

19 CLINICAL STUDY REPORT

All clinical data will be presented at the end of the study as data listings. These will be checked to confirm the lists accurately represents the data collected during the course of the study. The trial data will then be locked and a final data listing produced. The clinical study report will be based on the final data listings. The locked trial data may then be used for analysis and publication.

20 STUDY SITE MANAGEMENT

20.1 Study site responsibilities

The Principal Investigator (the PI or lead clinician for the study site) has overall responsibility for conduct of the study, but may delegate responsibility where appropriate to suitably experienced and trained members of the study site team. All members of the study site team must complete a delegation log prior to undertaking any study duties. The PI must counter sign and date each entry in a timely manner, authorising staff to take on the delegated responsibilities.

20.2 Study site set up and activation

A Principal Investigator should lead the study at the Oxford site, providing the local study office with all core documentation and attend a 'Site Training Call' organized by the trial office before the site becomes activated (the training call will usually be carried out as a telephone conference call but may be face to face meeting). The trial office will ensure the site has all the required study information/documentation and is ready to recruit. The site will then be notified once they are activated on the TARDOX database and able to recruit.

20.3 Arrangements for sites outside the UK

Not applicable to the TARDOX study.

20.4 Study documentation

The trial office will provide an Investigator File and Pharmacy File to the investigational site containing the documents needed to initiate and conduct the study. The trial office must review and approve any local changes made to any study documentation including patient information and consent forms prior to use. Additional documentation generated during the course of the trial, including relevant communications must be retained in the site files as necessary to reconstruct the conduct of the trial.

21 REGULATORY AND ETHICAL CONSIDERATIONS

The Sponsor and Investigators will ensure that this protocol will be conducted in compliance with the UK Clinical Trials Regulations[20], the principles of Good Clinical Practice (GCP) and the applicable policies of the sponsoring organisation. Together, these implement the ethical principles of the Declaration of Helsinki (1996) and the regulatory requirements for clinical trials of an investigational medicinal product under the European Union Clinical Trials Directive.

21.1 Ethical conduct of the trial and ethics approval

The protocol, patient information sheet, consent form and any other information that will be presented to potential trial patients (e.g. advertisements or information that supports or supplements the informed consent) will be

20 The Medicines for Human Use (Clinical Trials) Regulations (S.I. 2004/1031) and any subsequent amendments to it.

reviewed and approved by an appropriately constituted, independent Research Ethics Committee (REC). Principal Investigators will be approved by the REC.

21.2 Regulatory Authority approval

Approval to conduct the study will be obtained from the relevant Competent Authority in each participating country prior to initiating the study. In the UK the study will be conducted under a Medicines and Healthcare products Regulatory Agency (MHRA) Clinical Trial Authorisation (CTA).

21.3 NHS Research Governance

Investigators are responsible for ensuring they obtain local Trust management agreement to conduct the trial in accordance with local arrangements and policies.

21.4 Protocol amendments

Amendments are changes made to the research following initial approval. A 'substantial amendment' is an amendment to the terms of the Responsible Authority application (if applicable), the REC application, or to the protocol or any other supporting documentation, that is likely to affect to a significant degree:

- the safety or physical or mental integrity of the subjects of the trial;
- the scientific value of the trial;
- the conduct or management of the trial; or
- the quality or safety of the investigational medicinal product(s) used in the trial.

Non-substantial amendments are those where the change(s) involve only minor logistical or administrative aspects of the study.

All amendments will be generated and managed according to the trial office standard operating procedures to ensure compliance with applicable regulation and other requirements. Written confirmation of all applicable REC, regulatory and local approvals must be in place prior to implementation by Investigators. The only exceptions are for changes necessary to eliminate an immediate hazard to study patients (see below).

It is the Investigator's responsibility to update patients (or their authorised representatives, if applicable) whenever new information (in nature or severity) becomes available that might affect the patient's willingness to continue in the trial. The Investigator must ensure this is documented in the patient's medical notes and the patient is re-consented if appropriate.

21.5 Urgent safety measures

The sponsor or Investigator may take appropriate urgent safety measures to protect trial participants from any immediate hazard to their health or safety. Urgent safety measures may be taken without prior authorisation. The trial may continue with the urgent safety measures in place. **The Investigator must inform the trial office IMMEDIATELY if the study site initiates an urgent safety measure:**

Tel: 01865 227190
Fax: 01865 227039
Email: OCTO-TARDOX@oncology.ox.ac.uk

The notification must include:

- Date of the urgent safety measure;
- Who took the decision; and
- Why the action was taken.

The Investigator will provide any other information that may be required to enable the trial office to report and manage the urgent safety measure in accordance with the current regulatory and ethical requirements for expedited reporting and close out.

The trials office will follow written procedures to implement the changes accordingly.

21.6 Temporary halt

The sponsor and Investigators reserve the right to place recruitment to this protocol on hold for short periods for administrative reasons **or** to declare a temporary halt. A temporary halt is defined a formal decision to:

- interrupt the treatment of subjects already in the trial for safety reasons;
- stop recruitment on safety grounds; or
- stop recruitment for any other reason(s) considered to meet the substantial amendment criteria, including possible impact on the feasibility of completing the trial in a timely manner.

The trial office will report the temporary halt via an expedited substantial amendment procedure. The trial may not restart after a temporary halt until a further substantial amendment to re-open is in place. If it is decided not to restart the trial this will be reported as an early termination.

21.7 Serious Breaches

The Medicines for Human Use (Clinical Trials) Regulations require the Sponsor to notify any "serious breaches" to the MHRA within 7 days of the sponsor becoming aware of the breach. A serious breach is defined as “A breach of GCP or the trial protocol which is likely to effect to a significant degree:

- the safety or physical or mental integrity of the subjects of the trial; or
- the scientific value of the trial”

Investigators must notify the trials office at once if any serious breach of GCP is suspected. The TARDOX Trial Office will report all Serious Breaches to Clinical Trial Research Governance, University of Oxford.

21.8 REPORTS: Progress, Safety and End of Study Reports

This protocol will comply with all current applicable Regulatory Authority, Research Ethics Committee and Sponsor requirements for the provision of periodic study safety and progress reports. Any additional reports will be provided on request. Reporting will be managed by the trials office according to internal SOPs. The site will be urged to return as much data as possible before each database lock point.

The trial office will determine which reports need to be circulated Principal Investigators and other interested parties according to internal SOPs. The study site is responsible for forwarding trial reports they receive to their local Trust as required.

22 EXPENSES AND BENEFITS

Reasonable travel expenses for any visits additional to normal care will be reimbursed on production of receipts or a mileage allowance provided as appropriate.

23 QUALITY ASSURANCE

23.1 Risk assessment

A risk assessment and a monitoring plan will be prepared before the study opens. The risk assessment will be repeated if necessary in the light of changes while the study is on-going or in response to monitoring reports. Monitoring plans will be amended as appropriate.

23.2 On-site Monitoring

Data will be evaluated for compliance with the protocol and accuracy in relation to source documents. Monitoring visits reports will be sent to the site in a timely fashion.

23.3 Central Monitoring

The study site will be monitored centrally by both programmed validation within the data collection database and manual checking of incoming data for compliance with the protocol, data consistency, missing data and timing. All changes to data that could influence the outcome will be queried with and approved by the study site in a timely manner. For all other data, where there is no doubt about the source of any errors, clear changes to data will be made internally by TARDOX Trial Office staff without referring back to the study site. Study staff will be in regular contact

with site personnel to check on progress and deal with any queries that they may have including those arising from queries raised by the TARDOX Trial Office.

23.4 Audit and Regulatory Inspection

All aspects of the study conduct may be subject to internal or external quality assurance audit to ensure compliance with the protocol, GCP requirements and other applicable regulation or standards. It may also be subject to a regulatory inspection. Such audits or inspections may occur at any time during or after the completion of the study. Investigators and their host Institution(s) should understand that it is necessary to allow auditors/inspectors direct access to all relevant documents, study facilities and to allocate their time and the time of their staff to facilitate the audit or inspection visit. Anyone receiving notification of a Regulatory Inspection that will (or is likely to) involve this trial must inform the trial office without delay.

24 RECORDS RETENTION & ARCHIVING

During the clinical trial and after trial closure the Investigator must maintain adequate and accurate records to enable the conduct of a clinical trial and the quality of the research data to be evaluated and verified. All essential documents must be stored in such a way that ensures that they are readily available, upon request for the minimum period required by national legislation or for longer if needed. The medical files of trial subjects shall be retained in accordance with national legislation and in accordance with the host institution policy.

Retention and storage of laboratory records for clinical trial samples must also follow these guidelines:

- Retention and storage of central laboratory records supporting PK or PD endpoints and the disposition of samples donated via the trial must also comply with applicable legislation and Sponsor requirements.
- It is the University of Oxford's policy to store data for a minimum of 5 years. Investigators may not archive or destroy study essential documents or samples without written instruction from the trial office.

25 PATIENT CONFIDENTIALITY

Personal data recorded on all documents will be regarded as confidential, and to preserve each patient's anonymity, only their patient study number, initials and date of birth (or other identified as appropriate to country regulations and agreed with the Sponsor) will be recorded on the CRFs.

The Investigator site must maintain the patient's anonymity in all communications and reports related to the research. The Investigator site team must keep a separate log of enrolled patients' personal identification details as necessary to enable them to be tracked. These documents must be retained securely, in strict confidence. They form part of the Investigator Site File and are not to be released externally.

26 STUDY FUNDING

This trial is being organised by the Oncology Clinical Trials Office (OCTO) at the University of Oxford. The trial is part of the NIHR portfolio, and any additional NHS clinical service support costs of patient care while on study should be met by the host study site. Celsion is funding manufacture and supply of the study drug (ThermoDox®).

27 SPONSORSHIP AND INDEMNITY

27.1 Sponsorship

The Sponsor will provide written confirmation of Sponsorship and authorise the trial commencement once satisfied that all arrangements and approvals for the proper conduct of the trial are in place. A separate study delegation agreement, setting out the responsibilities of the Chief Investigator and Sponsor will be put in place between the parties.

27.2 Insurance

The University has a specialist insurance policy in place: - Newline Underwriting Management Ltd, at Lloyd's of London - which would operate in the event of any participant suffering harm as a result of their involvement in the research.

27.3 Contracts/Agreements

This trial is subject to the Sponsor's policy requiring that written contracts/agreements are agreed formally by the participating bodies as appropriate. A Clinical Trial Agreement (CTA) will be placed between the Sponsor and participating NHS Trust(s) prior to site activation.

The Sponsor will also set up written agreements with any other external third parties involved in the conduct of the trial as appropriate.

28 PUBLICATION POLICY

The intention is to publish this research in a specialist peer reviewed scientific journal on completion of the study. The results may also be presented at scientific meetings and/or used for a thesis. The Investigators will be involved in reviewing drafts of the manuscripts, abstracts, press releases and any other publications arising from the trial and retain final editorial control.

29 REFERENCES

1. Bengmark, S. and L. Hafstrom, *The natural history of primary and secondary malignant tumors of the liver. I. The prognosis for patients with hepatic metastases from colonic and rectal carcinoma by laparotomy.* Cancer, 1969. **23**(1): p. 198-202.
2. Jaffe, B.M., et al., *Factors influencing survival in patients with untreated hepatic metastases.* Surg Gynecol Obstet, 1968. **127**(1): p. 1-11.
3. Stangl, R., et al., *Factors influencing the natural history of colorectal liver metastases.* Lancet, 1994. **343**(8910): p. 1405-10.
4. Rothbarth, J. and C.J. van de Velde, *Treatment of liver metastases of colorectal cancer.* Ann Oncol, 2005. **16 Suppl 2**: p. ii144-9.
5. Berber, E., R. Pelley, and A.E. Siperstein, *Predictors of survival after radiofrequency thermal ablation of colorectal cancer metastases to the liver: a prospective study.* J Clin Oncol, 2005. **23**(7): p. 1358-64.
6. Bruix, J., M. Sherman, and A.A.f.t.S.o.L.D. Practice Guidelines Committee, *Management of hepatocellular carcinoma.* Hepatology, 2005. **42**(5): p. 1208-36.
7. Llovet, J.M., A. Burroughs, and J. Bruix, *Hepatocellular carcinoma.* Lancet, 2003. **362**(9399): p. 1907-17.
8. Bruix, J., et al., *Clinical management of hepatocellular carcinoma. Conclusions of the Barcelona-2000 EASL conference. European Association for the Study of the Liver.* J Hepatol, 2001. **35**(3): p. 421-30.
9. Coleman, M.P., et al., *EUROCARE-3 summary: cancer survival in Europe at the end of the 20th century.* Ann Oncol, 2003. **14 Suppl 5**: p. v128-49.
10. Poon, R.T. and N. Borys, *Lyso-thermosensitive liposomal doxorubicin: a novel approach to enhance efficacy of thermal ablation of liver cancer.* Expert Opin Pharmacother, 2009. **10**(2): p. 333-43.
11. Choi, H., et al., *Correlation of computed tomography and positron emission tomography in patients with metastatic gastrointestinal stromal tumor treated at a single institution with imatinib mesylate: proposal of new computed tomography response criteria.* J Clin Oncol, 2007. **25**(13): p. 1753-9.
12. Eisenhauer, E.A., et al., *New response evaluation criteria in solid tumours: revised RECIST guideline (version 1.1).* Eur J Cancer, 2009. **45**(2): p. 228-47.
13. Kinahan, P.E. and J.W. Fletcher, *Positron emission tomography-computed tomography standardized uptake values in clinical practice and assessing response to therapy.* Semin Ultrasound CT MR, 2010. **31**(6): p. 496-505.
14. Wanebo, H.J., et al., *Surgical management of patients with primary operable colorectal cancer and synchronous liver metastases.* Am J Surg, 1978. **135**(1): p. 81-5.
16. Welch, J.P. and G.A. Donaldson, *The clinical correlation of an autopsy study of recurrent colorectal cancer.* Ann Surg, 1979. **189**(4): p. 496-502.
17. Clark, G.M., et al., *Survival from first recurrence: relative importance of prognostic factors in 1,015 breast cancer patients.* J Clin Oncol, 1987. **5**(1): p. 55-61.

18. Goldhirsch, A., R.D. Gelber, and M. Castiglione, *Relapse of breast cancer after adjuvant treatment in premenopausal and perimenopausal women: patterns and prognoses.* J Clin Oncol, 1988. **6**(1): p. 89-97.
19. Insa, A., et al., *Prognostic factors predicting survival from first recurrence in patients with metastatic breast cancer: analysis of 439 patients.* Breast Cancer Res Treat, 1999. **56**(1): p. 67-78.
20. O'Reilly, S.M., M.A. Richards, and R.D. Rubens, *Liver metastases from breast cancer: the relationship between clinical, biochemical and pathological features and survival.* Eur J Cancer, 1990. **26**(5): p. 574-7.
21. Wyld, L., et al., *Prognostic factors for patients with hepatic metastases from breast cancer.* Br J Cancer, 2003. **89**(2): p. 284-90.
22. Bathe, O.F., et al., *Metastasectomy as a cytoreductive strategy for treatment of isolated pulmonary and hepatic metastases from breast cancer.* Surg Oncol, 1999. **8**(1): p. 35-42.
23. Quint, L.E., et al., *Distribution of distant metastases from newly diagnosed non-small cell lung cancer.* Ann Thorac Surg, 1996. **62**(1): p. 246-50.
24. Weiss, L., et al., *Haematogenous metastatic patterns in colonic carcinoma: an analysis of 1541 necropsies.* The Journal of pathology, 1986. **150**(3): p. 195-203.
25. Hilsenbeck, S.G., W.A. Raub, Jr., and K.S. Sridhar, *Prognostic factors in lung cancer based on multivariate analysis.* Am J Clin Oncol, 1993. **16**(4): p. 301-9.
26. Ries, L.A., *Influence of extent of disease, histology, and demographic factors on lung cancer survival in the SEER population-based data.* Seminars in surgical oncology, 1994. **10**(1): p. 21-30.
27. Adam, R., et al., *Is liver resection justified for patients with hepatic metastases from breast cancer?* Ann Surg, 2006. **244**(6): p. 897-907; discussion 907-8.
28. Fong, Y., et al., *Liver resection for colorectal metastases.* J Clin Oncol, 1997. **15**(3): p. 938-46.
29. Fong, Y., et al., *Clinical score for predicting recurrence after hepatic resection for metastatic colorectal cancer: analysis of 1001 consecutive cases.* Ann Surg, 1999. **230**(3): p. 309-18; discussion 318-21.
30. Hughes, K.S., et al., *Resection of the liver for colorectal carcinoma metastases: a multi-institutional study of patterns of recurrence.* Surgery, 1986. **100**(2): p. 278-84.
31. Bismuth, H., et al., *Resection of nonresectable liver metastases from colorectal cancer after neoadjuvant chemotherapy.* Ann Surg, 1996. **224**(4): p. 509-20; discussion 520-2.
32. Kemeny, N. and A. Schneider, *Regional treatment of hepatic metastases and hepatocellular carcinoma.* Curr Probl Cancer, 1989. **13**(4): p. 197-283.
33. Doroshow, N.P., *Modulation of fluorouracil by leucovorin in patients with advanced colorectal cancer: evidence in terms of response rate. Advanced Colorectal Cancer Meta-Analysis Project.* J Clin Oncol, 1992. **10**(6): p. 896-903.
34. Piedbois, P., Rougier, P., Buyse, M., Pignon, J., Ryan, L., Hansen, R., Zee, B., Weinerman, B., Pater, J., Leichman, C., *Efficacy of intravenous continuous infusion of fluorouracil compared with bolus administration in advanced colorectal cancer. Meta-analysis Group In Cancer.* J Clin Oncol, 1998. **16**(1): p. 301-8.
35. Flanders, V.L. and D.A. Gervais, *Ablation of liver metastases: current status.* J Vasc Interv Radiol, 2010. **21**(8 Suppl): p. S214-22.
36. Chuang, V.P. and S. Wallace, *Hepatic artery embolization in the treatment of hepatic neoplasms.* Radiology, 1981. **140**(1): p. 51-8.
37. Clouse, M.E., et al., *Peripheral hepatic artery embolization for primary and secondary hepatic neoplasms.* Radiology, 1983. **147**(2): p. 407-11.
38. Tellez, C., et al., *Phase II trial of chemoembolization for the treatment of metastatic colorectal carcinoma to the liver and review of the literature.* Cancer, 1998. **82**(7): p. 1250-9.
39. Solbiati, L., et al., *Percutaneous radio-frequency ablation of hepatic metastases from colorectal cancer: long-term results in 117 patients.* Radiology, 2001. **221**(1): p. 159-66.
40. Parkin, D.M., et al., *Global cancer statistics, 2002.* CA Cancer J Clin, 2005. **55**(2): p. 74-108.
41. Khan, S.A., et al., *Rising trends in cholangiocarcinoma: is the ICD classification system misleading us?* J Hepatol, 2012. **56**(4): p. 848-54.
42. Llovet, J.M., et al., *Sorafenib in advanced hepatocellular carcinoma.* N Engl J Med, 2008. **359**(4): p. 378-90.
43. Valle, J.W., et al., *Gemcitabine alone or in combination with cisplatin in patients with advanced or metastatic cholangiocarcinomas or other biliary tract tumours: a multicentre randomised phase II study - The UK ABC-01 Study.* Br J Cancer, 2009. **101**(4): p. 621-7.
44. Celsion, *ThermoDox® (Lyso-Thermosensitive Liposomal Doxorubicin, LTLD) Investigator's Brochure (Unpublished)*.
45. Illing, R.O., et al., *The safety and feasibility of extracorporeal high-intensity focused ultrasound (HIFU) for the treatment of liver and kidney tumours in a Western population.* Br J Cancer, 2005. **93**(8): p. 890-5.
46. Leslie, T.A., et al., *High-intensity focused ultrasound ablation of liver tumours: can radiological assessment predict the histological response?* Br J Radiol, 2008. **81**(967): p. 564-71.

47. Fandrich, C.A., R.P. Davies, and P.M. Hall, *Small gauge gelfoam plug liver biopsy in high risk patients: safety and diagnostic value.* Australas Radiol, 1996. **40**(3): p. 230-4.
48. Howlett, D.C., et al., *Findings of the UK national audit evaluating image-guided or image-assisted liver biopsy. Part II. Minor and major complications and procedure-related mortality.* Radiology, 2013. **266**(1): p. 226-35.
49. Child, C.G. and J.G. Turcotte, *Surgery and portal hypertension.* Major Probl Clin Surg, 1964. **1**: p. 1-85.
50. Pugh, R.N., et al., *Transection of the oesophagus for bleeding oesophageal varices.* Br J Surg, 1973. **60**(8): p. 646-9.

APPENDIX 1: WHO PERFORMANCE SCALE

Activity Performance Description	Score
Fully active, able to carry out all on all pre-disease performance without restriction.	0
Restricted in physically strenuous activity but ambulatory and able to carry out work of a light or sedentary nature, e.g. light housework, office work.	1
Ambulatory and capable of all self-care, but unable to carry out any work activities. Up and about more than 50% of waking hours.	2
Capable of only limited self-care. Confined to bed or chair more than 50% of waking hours.	3
Completely disabled. Cannot carry out any self-care. Totally confined to bed or chair.	4

APPENDIX 2: AMERICIAN SOCIETY OF ANAESTHESIOLOGISTS (ASA) CLASSIFICATION SYSTEM

Activity Performance Description	Score
Healthy Person.	ASA 1
Mild systemic disease.	ASA 2
Severe systemic disease.	ASA 3
Severe systemic disease that is a constant threat to life.	ASA 4
A moribund person who is not expected to survive without the operation	ASA 5
A declared brain-dead person whose organs are being removed for donor purposes	ASA 6

APPENDIX 3: CHILD-PUGH SCORE

The Child-Pugh Score [49, 50] is used to assess chronic liver disease. It is calculated by adding the points for the five criteria assessed:

Points	1	2	3
Encephalopathy	None	Minimal	Advanced (coma)
Ascites	Absent	Controlled	Refractory
Bilirubin (µmol/L) (mg/dl)	<34<br><2	34-50<br>2-3	>50<br>>3
Albumin (g/dL)	>3.5	2.8-3.5	<2.8
INR	<1.7	1.71-2.30	>2.30

The Child-Pugh Score is interpreted as follows:

Points	Class	One year survival	Two year survival
5-6	A	100%	85%
7-9	B	81%	57%
10-15	C	45%	35%
